# Chemical Constituents from the Fruits of *Amomum kravanh* and Their Role in Activating Alcohol Dehydrogenase

**DOI:** 10.3390/molecules28124878

**Published:** 2023-06-20

**Authors:** Hao-Ming Xiong, Hui-Ying Li, Zhi-Rong Lin, Xiao-Mei Liu, Li-Ping Bai, Wei Zhang, Zhi-Hong Jiang, Guo-Yuan Zhu

**Affiliations:** State Key Laboratory of Quality Research in Chinese Medicine, Guangdong-Hong Kong-Macao Joint Laboratory of Respiratory Infectious Disease, Macau Institute for Applied Research in Medicine and Health, Macau University of Science and Technology, Macau 999078, China; 2009853vct20001@student.must.edu.mo (H.-M.X.); 2220010732@student.must.edu.mo (H.-Y.L.); 2009853gct20004@student.must.edu.mo (Z.-R.L.); 2109853gct30002@student.must.edu.mo (X.-M.L.); lpbai@must.edu.mo (L.-P.B.); wzhang@must.edu.mo (W.Z.)

**Keywords:** *Amomum kravanh*, amomumols A-J, sesquiterpenoids, monoterpenes, alcohol dehydrogenase

## Abstract

Alcoholism is a worldwide health problem, and diseases caused by alcoholism are killing people every year. *Amomum kravanh* is a traditional Chinese medicine used to relieve hangovers. However, whether its bioactive components improve alcohol metabolism is not clear. In this study, ten new (amomumols A-J, **1**–**10**) and thirty-five known (**11**–**45**) compounds were isolated from the fruits of *Amomum kravanh* by an activity-guided separation. Ten novel compounds were identified as four sesquiterpenoids (**1**–**4**), three monoterpene derivatives (**5**–**7**), two neolignans (**8**, **9**), and a novel norsesquiterpenoid (**10**) with a new C_14_ nor-bisabolane skeleton. Their structures were determined by the comprehensive analysis of high-resolution electrospray ionization mass spectrometry (HRESIMS), nuclear magnetic resonance (NMR), and electronic circular dichroism (ECD) calculation. The effects of all isolated compounds on the activity of alcohol dehydrogenase were evaluated in vitro, and it was found that eight compounds (**11**, **12**, **15**, **18**, **26**, and **36**–**38**) exhibited significant activation effects on the alcohol dehydrogenase at 50 μM.

## 1. Introduction

Drinking culture is shared by all nationalities in the world. However, regarding public health, heavy drinking has become a thorny global healthcare problem [1]. As the leading metabolic site of alcohol, the liver is responsible for most of the damage caused by alcohol metabolism. Heavy drinking results in a high incidence of alcoholic liver diseases (ALD), including fatty lesions, liver fibrosis, cirrhosis, and even acute and chronic hepatitis and liver cancer [2,3]. In addition to the liver, the brain, heart, and gastrointestinal tract will be partially damaged by excessive alcohol consumption [4,5,6]. To date, there is no specific drug to help people eliminate alcoholism, and few drugs, such as metadoxine, interleukin-22 analogues, and interleukin-1*β* antagonists, are available for ALD [7]. In Asia, including China, Japan, and Korea, botanical medicines are traditionally used to prevent and treat alcohol-related diseases [8,9].

White cardamom, the fruits of *Amomum kravanh* Pierre ex Gagnep and *Amomum compactum* Soland ex Maton (Zingiberaceae), is used worldwide as a spice in cooking to change the taste of food. In China, cardamom is used as a spice as well as herbal medicine to treat stomach and digestive diseases. Chinese people also use cardamom to promote alcohol metabolism, which was recorded in many ancient traditional medicine books [10]. However, the ingredients and mechanism of cardamom to promote alcohol metabolism have not been studied deeply. *A. kravanh* is a tropical plant native to Cambodia and Thailand. Previous phytochemical investigations showed that the main components of *A. kravanh* are volatile oil, diterpenoids, flavonoids, steroids, diarylheptane, and lignans [10,11,12]. These compounds showed various pharmacological effects, including anti-inflammatory, antibacterial, anti-cancer, hypoglycemic, and gastric protection [13,14,15,16,17].

**Figure 1:**
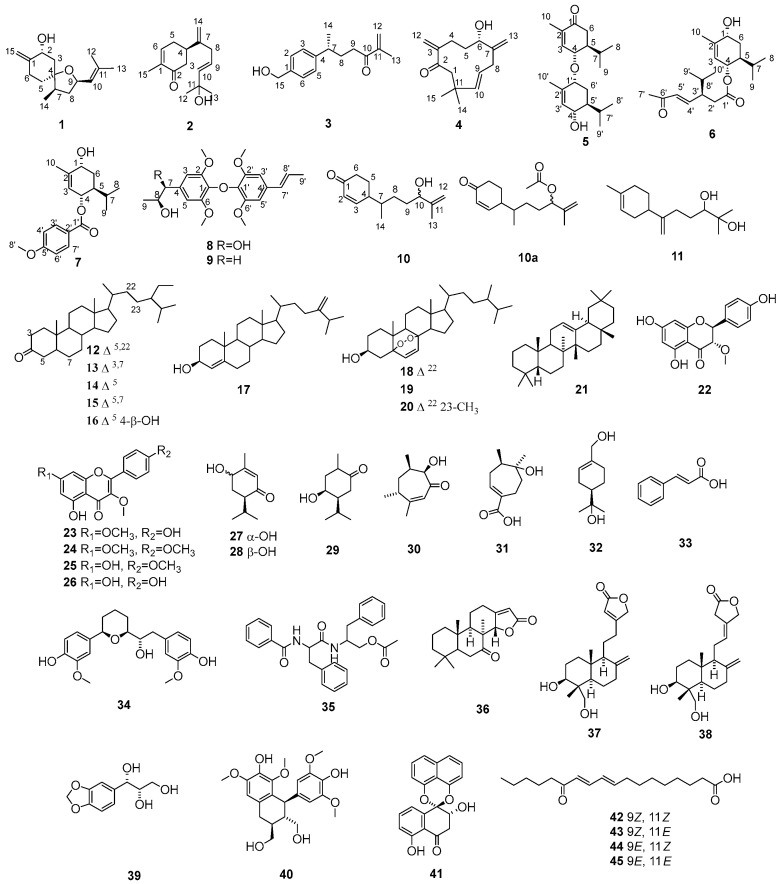
Chemical structures of compounds **1**–**45**.

In this study, we tried to find bioactive compounds from the fruit of *A. kravanh* that have the potential to help alcohol metabolism. An in vitro alcohol dehydrogenase activity (ADH) assay was used for the activity-guided isolation. It led to the isolation of 10 new (**1**–**10**) and 35 known (**11**–**45**) compounds (Figure 1) from the fruits of *A. kravanh* and the identification of 8 bioactive compounds (**11**, **12**, **15**, **18**, **26**, and **36**–**38**) with alcohol dehydrogenase activation. Herein, the isolation, structural elucidation, and bioassay of 10 new (**1**–**10**) and 35 known (**11**–**45**) compounds from the fruits of *A. kravanh* are reported.

## 2. Results and Discussion

The fruits of *A. kravanh* (10 kg) were extracted with 80% EtOH under reflux and then partitioned successively with petroleum ether (PE), EtOAc, and *n*-BuOH. The effects of these extracts on alcohol dehydrogenase activation were screened. Results showed that PE and EtOAc fractions could significantly activate alcohol dehydrogenase (Figure S103). Therefore, PE and EtOAc fractions were subjected to silica gel column chromatography and further purified by repeated MPLC and HPLC to obtain four new sesquiterpenoids (**1**–**4**), three new monoterpene derivatives (**5**–**7**), two new neolignans (**8**, **9**), a novel norsesquiterpenoid (**10**), and thirty-five known compounds (**11**–**45**) (Figure 1). 

### 2.1. Structural Elucidation

**Figure 2:**
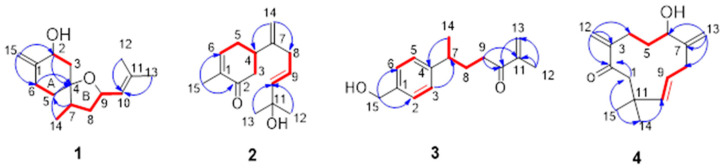
The key HMBC (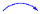) and ^1^H-^1^H COSY (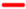) correlations of compounds **1**–**4**.

Compound **1** was obtained as a pale-yellow oil. Its molecular formula C_15_H_24_O_2_ was calculated by HRESIMS ion at *m*/*z* 237.1848 [M + H]^+^ (calculated for C_15_H_25_O_2_, 237.1849), corresponding to four degrees of unsaturation. In the ^1^H NMR spectrum, three methyl signals at *δ*_H_ 0.93 (3H, d, *J* = 6.9 Hz, H-14), 1.68 (3H, s, H-12), and 1.72 (3H, s, H-13), two oxygenated protons at *δ*_H_ 4.46 (1H, dd, *J* = 11.6, 4.8 Hz, H-2) and 4.66 (1H, ddd, *J* = 9.8, 8.5, 6.0 Hz, H-9), a group of extracyclic double bond resonances at *δ*_H_ 4.92 and 4.78 (2H, s, H-15), and an olefinic proton at *δ*_H_ 5.16 (1H, br d, *J* = 8.5 Hz, H-10) were observed (Table 1). Its ^13^C NMR and DEPT data (Table 1) showed 15 resonances, which can be classified as three methyls (*δ*_C_ 14.3, 18.1, and 25.8), two methines (*δ*_C_ 43.6 and 127.4), two oxygenated methines (*δ*_C_ 69.8 and 73.9), an olefinic methylene (*δ*_C_ 103.2), four methylenes (*δ*_C_ 29.5, 33.5, 40.7, and 46.8), two olefinic quaternary carbons (*δ*_C_ 134.6 and 151.8), and one oxygenated quaternary carbon (*δ*_C_ 84.1). These NMR data indicated that **1** is a sesquiterpenoid. Two double bonds occupied two degrees of unsaturation, and the remaining two indexes of hydrogen deficiency required a dicyclic skeleton for **1**. The A-ring fragment in the structure (Figure 2) was established by HMBC correlations from H-15 to C-1/C-2/C-6, from H-2/H-6 to C-4, and ^1^H-^1^H COSY correlations (Figure 2) of H-2/H-3 and H-5/H-6. By the ^1^H-^1^H COSY correlations of H-14/H-7/H-8/H-9/H-10 and HMBC correlations from H-12/H-13 to C-10, a 2-methylhept-2-ene fragment was deduced. The HMBC correlations between H-14 and C-4 and between H-5 and C-7 suggested that these two fragments were connected with the C4–C7 bond. According to the molecular formula and NMR data, a remaining tetrahydrofuran ring (B-ring) was deduced, which framed a specific 1-oxaspiro [4.5] decane ring system of **1**. Thus, the plane structure of the compound was determined.

**Figure 3:**
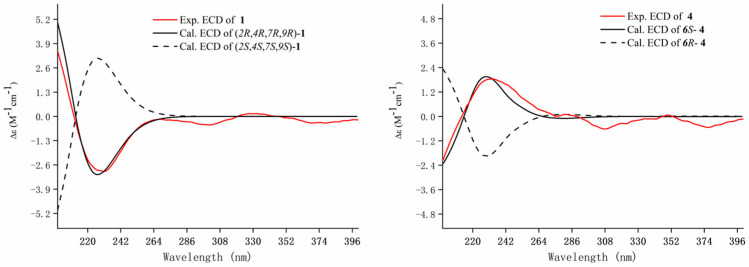
Experimental and calculated ECD spectra of compounds **1** and **4**.

Meanwhile, the NOE correlations between H-2/H-3a/H-14 indicated that they are on the same side and were assigned *β*-orientation. The *α*-orientation of H-9/H-7 was determined by the NOE correlations of H-5/H-9/H-7. Finally, the calculated ECD spectra of (2*R*,4*R*,7*R*,9*R*)-**1** and (2*S*,4*S*,7*S*,9*S*)-**1** were compared to the experimental ECD of **1** and found that it was matched well with that of (2*R*,4*R*,7*R*,9*R*)-**1** (Figure 3). Thus, the absolute configuration of **1** was defined as (2*R*,4*R*,7*R*,9*R*). Therefore, the structure of **1** was established as shown and named amomumol A.

Compound **2** is a yellowish oil and has a molecular formula C_15_H_22_O_2_ deduced from the [M + H]^+^ ion at *m*/*z* 235.1701 (calc. 235.1693). The NMR data of **2** (Table 1) were similar to those of (-)-(6*R*)-11-hydroxybisabol-2,7(14)-dien-4-one, a bisabolane sesquiterpenoid isolated from the aerial parts of *Mikania shushunensis* [18], except for that an additional double bond [*δ*_H_ 5.61 (1H, t, *J* = 20.1 Hz, H-9), 5.65 (1H, t, *J* = 5.9 Hz, H-10); *δ*_C_ 199.7 (C-9), 111.1 (C-9)] in **2** was observed. The double bond was assigned at C9-C10 based on the HMBC correlations from H-12/H-13 to C-10 and ^1^H-^1^H COSY correlations of H-8/H-9/H-10 (Figure 2). The negative rotation of **2** was comparable to that of (-)-(6*R*)-11-hydroxybisabol-2,7(14)-dien-4-one, which indicated that they possess the same absolute configuration of C-4, i.e., 4*R* [19]. Thus, the structure of **2** was elucidated and named amomumol B.

Compound **3** has a molecular formula C_15_H_20_O_2_ with six degrees of unsaturation. Detailed analysis of ^1^H and ^13^C-NMR of **3** (Table 1) suggested that **3** has the same bisabolane skeleton as **2**. Two pairs of specific aromatic protons (*δ*_H_ 7.18, 2H, d, *J* = 7.9 Hz; 7.30, 2H, d, *J* = 7.9 Hz) corresponding to para-disubstituted benzene indicated that the ring of **3** is aromatized, which was similar to bisabolane-type sesquiterpenoid derivatives isolated from the bark of Peltophorum dasyrachis [18]. A hydroxyl group was substituted at C-15 [*δ*_H_ 4.68 (2H, s), *δ*_C_ 65.2], confirmed by the HMBC correlations from H-15 to C-1, C-2, and C-6 (Figure 2). An *α*,*β*-unsaturated ketone group was assigned at the end of the side train based on the HMBC correlations from H-13 to C-10, C-11, and C-12 (Figure 2). By comparing the specific rotation of (+)-(*S*)-ar-turmerone [18], a bisabolane-type sesquiterpenoid derivative having the same chiral center at C-7 with **3**, the absolute configuration of **3** was determined as 7*R* supported by its negative rotation. Finally, the structure of **3** was determined as shown and named amomumol C.

Compound **4** was obtained as yellowish oil with a molecular formula C_15_H_22_O_2_ calculated by the HRESIMS ion at *m*/*z* 235.1694 [M + H]^+^ (calc. 235.1693). ^1^H and ^13^C-NMR data of **4** (Table 1) were closely comparable to those of (2*R*,6*S*)-2,6-dihydroxylhumla-9*E*,3(12),7(13),9-triene [20], except that a hydroxy group was replaced by a ketone in **4**. The planar structure of **4** was then fully elucidated by 2D NMR data (Figure 2). It was found that the positive cotton effect at 230 nm in the ECD of **4** matched well with those of the calculated ECD of 6*S*-**4**. Therefore, the absolute configuration of **4** was assigned to be 6*S* and named amomumol D.

**Figure 4:**
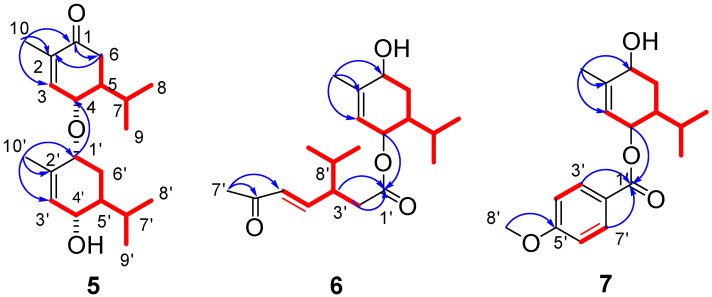
The key HMBC (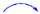) and ^1^H-^1^H COSY (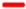) correlations of compounds **5**–**7**.

**Figure 5:**
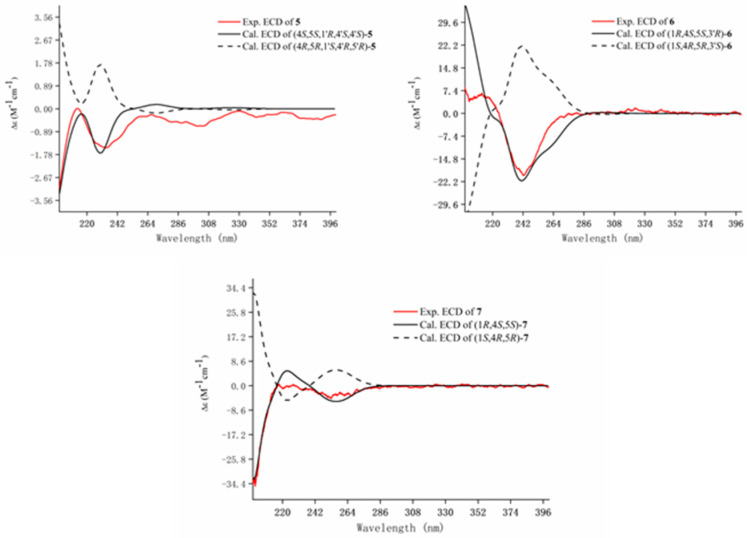
Experimental and calculated ECD spectra of compounds **5**–**7**.

The molecular formula of **5** was calculated to be C_20_H_32_O_3_ by the HRESIMS ion peak at *m*/*z* 321.2429 [M + H]^+^ (calc. 321.2424). ^1^H NMR spectrum of **5** (Table 2) showed four methyl doublets [*δ*_H_ 0.85 (3H, d, *J* = 6.9 Hz, H-8′), 0.87 (3H, d, *J* = 6.9 Hz, H-8), 0.91 (3H, d, *J* = 6.9 Hz, H-9), and 0.98 (3H, d, *J* = 6.9 Hz, H-9′)], two methyl singlets [*δ*_H_ 1.83 (3H, s, H-10) and 1.81 (3H, s, H-10′)], three oxygenated protons [*δ*_H_ 3.77 (1H, br s, H-1′), 3.92 (1H, d, *J* = 9.1 Hz, H-4′), and 3.98 (1H, d, *J* = 9.4 Hz, H-4)], two olefinic protons [*δ*_H_ 5.63 (1H, br s, H-3′), 6.73 (1H, br s, H-3)]. The ^13^C-NMR, DEPT, and HSQC spectra revealed 20 carbon resonances, including six methyls, four olefinic carbons, three oxygenated carbons, two methylenes, four methines, and a carbonyl carbon (Table 2). The above MS and NMR data indicated that **5** is a monoterpenoid dimer. The HMBC correlations (Figure 4) from H-10 to C-1/C-2/C-3 and H-10′ to C-1′/C-2′/C-3′ combining with ^1^H-^1^H COSY correlations (Figure 4) of H-3/H-4/H-5/H-6, H-5/H-7/H-8(H-9), H-3′/H-4′/H-5′/H-6′/H-1′, and H-5′/H-7′/H-8′(H-9′) deduced two similar monoterpene fragments as 4α-hydroxy-1-oxo-p-menth-3(2)-ene in **5**. Two monoterpenes were connected by an oxygen bridge at C4–O–C1′ based on the HMBC correlation (Figure 4) between H-4 and C-1′. The big coupling constants of H-4 (*J* = 9.4 Hz) with H-5 and H-4′ (*J* = 9.1 Hz) with H-5′ indicated that H-4 and H-4′ were *β*-oriented and H-5 and H-5′ were α-oriented, which are the same as those of monoterpenes reported in the literature [21,22]. The NOESY correlation from H-4′ to H- 1′ suggested H- 1′ was also *β*-oriented. Supporting this determination, the experimental ECD of **5** showed a negative cotton effect at 200 and 225 nm and matched well with the calculated ECD curve of (4*S*,5*S*,1′*R*,4′*S*,5′*S*)-**5** (Figure 5). The absolute configuration of **5** was identified as (4*S*,5*S*,1′*R*,4′*S*,5′*S*) and it was named amomumol E.

Amomumol F (**6**) had a molecular formula C_20_H_32_O_4_ based on its HRESIMS ion at m/z 359.2184 [M + Na]^+^ (calc. 359.21934), corresponding to 5 degrees of unsaturation. Comprehensive ^1^H and ^13^C NMR data analysis revealed that **6** is a similar derivative of **5**. A 1,4-dihydroxy-menth-3(2)-ene moiety was first deduced by HMBC correlations (Figure 4) from H-10 to C-1/C-2/C-3 and ^1^H-^1^H COSY correlations (Figure 4) of H-3/H-4/H-5/H-6, H-5/H-7/H-8(H-9). ^1^H and ^13^C NMR data (Table 2) showed two double bonds and two carbonyl groups, which, combined with the ring of 1,4-dihydroxy-menth-3(2)-ene moiety, occupied all five degrees of unsaturation, meaning the remaining monoterpene is a chain monoterpene. The HMBC correlations from H-2′/H-3′ to C-1′ and from H-7′ to C-6′/C-5′ as well as ^1^H-^1^H COSY correlations of H-2′/H-3′/H-4′/H-5′ and H-3′/H-8′/H-9′ (H-10′) established another monoterpene fragment, which could be derived from 1,4-dihydroxy-menth-3(2)-ene after undergoing dehydration and oxidation ring-opening at C1–C2. The relative configuration of **6** was determined as the same as **5** due to the similar NMR data and biogenetic origination. The absolute configuration of **6** was determined as (1*R*,4*S*,5*S*,3′*R*) by ECD calculation (Figure 5).

Compound **7** (amomumol G) had a molecular formula C_18_H_24_O_4_ corresponding to its HRESIMS ion at *m*/*z* 327.1568 [M + Na]^+^ (calc. 327.1567). NMR data showed that **7** is also a monoterpene derivative. The monoterpene moiety was easily determined as 1,4-dihydroxy-menth-3(2)-ene inferred by HMBC correlations (Figure 4) from H-10 to C-1/C-2/C-3 and ^1^H-^1^H COSY correlations (Figure 4) of H-3/H-4/H-5/H-6, H-5/H-7/H-8(H-9). A para-disubstituted benzene fragment was indicated by two pairs of specific aromatic protons at *δ*_H_ 6.93 (2H, d, *J* = 9.0 Hz, H-4′,6′) and 8.0 (2H, d, *J* = 9.0 Hz, H-3′,7′). Combining with HMBC correlations between the methoxy group (*δ*_H_ 3.86, s) and C-5′, and between H-3′,7′ and the carbonyl carbon (*δ*_C_ 166.6) determined that the remaining substructure is a *p*-methoxy benzoic acid. The HMBC correlation from H-4 to C-1′ revealed that **7** is 1,4-dihydroxy-menth-3(2)-ene-4-*p*-methoxybenzoate. Similar to compound **6**, the absolute configuration of **7** was determined as (1*R*,4*S*,5*S*) by comparable experimental and calculated ECD curves with a positive cotton effect at 225 nm and a negative cotton effect at 256 nm (Figure 5).

**Figure 6:**
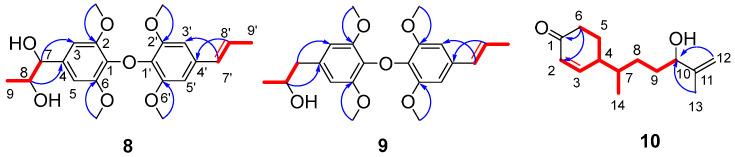
The key HMBC (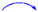) and ^1^H-^1^H COSY (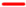) correlations of compounds **8**–**10**.

Amomumol H (**8**) was obtained as an amorphous powder, and its molecular formula was established to be C_22_H_28_O_7_ by the HRESIMS [M + Na]^+^ ion at *m*/*z* 427.1735 (calc. 427.1727). The NMR data (Table 3) were closely similar to alismaines A and B, two diphenylpropanoid ethers from *Alismatis Rhizoma* [23]. Two hydroxymethyl groups at C-9 and C-9′ and a methoxy group C-7 in alismaines A and B were replaced by two methyl groups and a hydroxy group in **8**. The ^1^H-^1^H COSY correlations between H-8 (*δ*_H_ 4.32, dq, *J* = 3.6, 6.3 Hz) and H-7 (*δ*_H_ 4.79, d, *J* = 3.6 Hz)/H-9 (*δ*_H_ 1.15, d, *J* = 6.3 Hz), between H-8′ (*δ*_H_ 6.24, dq, *J* = 6.5, 15.7 Hz) and H-7′ (*δ*_H_ 6.36, d, *J* = 15.7 Hz)/H-9′ (*δ*_H_ 1.88, d, *J* = 6.5 Hz), and other 2D NMR data (Figure 6) fully supported the structure of **8**. The small coupling constants of *J*_7,8_ (3.6 Hz) indicated the *erythro* configuration of H-7/H-8 (for the *threo* structure *J*_7,8_ is usually 8 Hz) [23,24]. The opposite optical rotation and ECD curve of **8** compared with those of alismaine A (7*S*,8*R*) suggested that the absolute configuration of **8** is (7*R*,8*S*).

The molecular formula C_22_H_28_O_6_ of **9** has one less oxygen atom than that of **8**. The ^1^H and ^13^C NMR data (Table 3) of **9** were very similar to those of **8**. The difference between the two compounds is that **9** possesses methylene at C-7 instead of an oxygenated methine of **8**. The structure of **9** was then fully elucidated by 2D NMR data analysis (Figure 6). Compound **9** has only one chiral carbon at C-8, but no cotton effect in the ECD spectrum of **9** was observed. Therefore, **9** was considered to be a racemate and named amomumol I.

Compound **10** (amomumol J) has a molecular formula C_14_H_22_O_2_ calculated by its HRESIMS ion at *m*/*z* 223.1697 [M + H]^+^. The ^1^H and ^13^C NMR data (Table 1) of **10** were similar to those of **2**, indicating that **10** is also a bisabolene-type sesquiterpenoid derivate. The molecular formula C_14_H_22_O_2_ and NMR data of **10** indicated that the structure of **10** lost a methyl group (C-15) of **2**. The ^1^H-^1^H COSY correlations of H-2/H-3/H-4/H-5/H-6 and HMBC correlations from H-3 (*δ*_H_ 7.03) and H-5 (*δ*_H_ 2.00, 1.79) to C-1(*δ*_C_ 202.7) formed the ring structure of **10**. HMBC correlations from H-12 (*δ*_H_ 4.94, 4.84) to C-10 (*δ*_C_ 76.6) and C-13 (*δ*_C_ 17.6) and ^1^H-^1^H COSY correlations of H-14/H-7/H-8/H-9/H-10 established the 2-methyl-3-hydroxy-hept-1-ene side train (Figure 6). As with other bisabolene-type sesquiterpenoids, the ring and side train moieties were connected by C4–C7 bond. In the ^13^C NMR spectrum of **10**, the carbon resonances corresponding to the 2-methyl-3-hydroxy-hept-1-ene side train showed a pair of resonances for each carbon, which may be caused by the different configurations of C-10. To separate the possible isomer of **10**, we tried different HPLC conditions, including the chiral column. However, compound **10** always showed a peak in different HPLC chromatographies. Next, we modified the hydroxyl group at C-10 of **10** to obtain the acetate of **10** (**10a**). In the ^13^C NMR spectrum of **10a**, the split carbons were still observed. Unfortunately, the separation of **10a** was not successive. Although the configuration of **10** has not been determined, compound **10** is the first example of norsesquiterpenoid with a new C14 nor-bisabolane skeleton.

Thirty-five known compounds were identified based on their MS and NMR data (**11**–**45**): 2-methyl-6-(4-methyl-3-cyclohexen-1-yl)-6-heptene-2,3-diol (**11**) [25], stigmasta-4,22-dien-3-one (**12**) [11], stigmasta-1,5-dien-3-one (**13**) [11], stigmast-4-en-3-one (**14**) [11], stigmasta-4,6-dien-3-one (**15**) [11], *β*-rosasterol (**16**) [26], ergosta-4,24(28)-dien-3-ol (**17**) [27], ergosterol peroxide (**18**) [28], 5,8-epidioxyergost-6-en-3-ol (**19**) [29], 5,8-epidioxy-23-methylergosta-6,22-dien-3-ol (**20**) [30], olean-12-ene (**21**) [31], 2,3-dihydro-5,7-dihydroxy-2-(4-hydroxyphenyl)-3-methoxy-4*H*-1-benzopyran-4-one (**22**) [32], kumatakenin (**23**) [33], ermanin (**24**) [34], 5-hydroxy-3,7,4′-trimethoxyflavone (**25**) [11], isokaempferide (**26**) [11], (4*S*,6*R*)-4-hydroxy-3-methyl-6-(1-methylethyl)-2-cyclohexen-1-one (**27**) [11], (4*R*,6*R*)-4-hydroxy-3-methyl-6-(1-methylethyl)-2-cyclohexen-1-one (**28**) [11], 4-hydroxy-2-methyl-5-(1-methylethyl)cyclohexanone (**29**) [11], *rel*-(+)-(4*R*,6*R*,7*R*)-7-hydroxy-3,4,6-trimethyl-2-cyclohepten-1-one (**30**) [35], *rel*-(+)-(4*R*,5*S*)-5-hydroxy-4,5-dimethyl-1-cycloheptene-1-carboxylic acid (**31**) [36], *p*-menth-1-ene-7,8-diol (**32**) [37], cinnamic acid (**33**) [37], kravanol B (**34**) [12], tifentai (**35**) [38], kravanhin B (**36**) [10], 3*β*,18-dihydroxylabda-8 (17), 13-dien-15,16-olide (**37**) [10], (12*E*)-3*β*,18-dihydroxylabda-8(17),12-dien-16,15-olide (**38**) [10], 1-(1,3-benzodioxol-5-yl)-1,2,3-propanetriol (**39**) [39], lyoniresinol (**40**) [40], 2,3-dihydro-2,5-dihydroxy-spiro[naphthalene-1(4*H*),2′-naphtho [1,8-de][1,3]dioxin]-4-one (**41**) [41], and 13-oxo-9*Z*(*E*),11*E*(*Z*)-octadecadienoic acid (**42–45**) [42].

### 2.2. In Vitro Alcohol Dehydrogenase (ADH) Promoting Activity of Isolated Compounds

**Figure 7:**
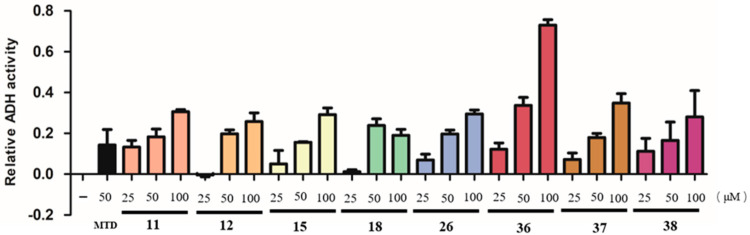
Compounds **11**, **12**, **15**, **18**, **26**, and **36**–**38** enhanced ADH enzyme activity in vitro (positive control: metadoxine).

Inspired by the results of the activity screening of the extracts, we evaluated the effects of 45 purified compounds on ADH enzyme activity by in vitro assays. All compounds were first screened using 50 μM of test compounds, and then concentration-dependent experiments (25, 50, and 100 μM) were performed on compounds with enhancing ADH enzyme activity. It was found that **11**, **12**, **15**, **18**, **26**, and **36**–**38** showed a concentration-dependent increase in ADH enzyme activity (Figure 7). These eight compounds belong to three steroids (**12**, **15**, and **18**), one flavonoid (**27**), one sesquiterpenoid (**11**), and three diterpenoids (**36**–**38**). Kravanhin B (**36**), a hemanthane-type diterpenoid, exhibited the most enhancing ADH enzyme activity compared to other compounds. Steroids with the peroxo bridge group showed the potential to activate ADH more than other steroids. This is the time the effects of these known compounds on ADH activation have been reported. These results suggested that the effect of *A. kravanh* on improving alcohol metabolism may come from the impact of the combination of multiple components.

## 3. Materials and Methods

### 3.1. General Experimental Procedures

Optical rotation was collected with Rudolph Research Analytical Autopol I automatic polarimeter. The acquisition of UV and CD spectra was performed on a Jasco *J*-1500 circular dichroism spectrometer. Data for IR spectra were collected using an Agilent Cary 660 Series IR spectrometer (KBr, Santa Clara, CA, USA). All NMR data were obtained on a Bruker Ascend 600 NMR spectrometer, and samples were dissolved in deuterated chloroform (CDCl_3_) or deuterated methanol (MEOD) with tetramethylsilane (TMS) as an internal reference. High-resolution electrospray ionization mass spectrometry (HRESIMS) analysis was performed on an Agilent 6230 MS spectrometer. Column chromatography (CC) used 40–63 μm chromatographic silica (Divisil, Germany). Medium pressure liquid chromatography (MPLC) was constructed by Buchi Sepacore flash system using RP-18 column (Unisil C18, 36 × 460 mm ID, 10–120 μm) and MCI column (Unips, 46 × 460 mm ID, 40–300 μm). HPLC purification was performed on Thermo Scientific Dionex ultimate 3000 UHPLC system or Agilent 1200 HPLC system equipped with a Waters Xbridge BEH C8 column (10 × 250 mm, 5 μm), an Atlantis T3 column (10 × 250 mm, 5 μm), an Xselect CSH Phenyl-Hexyl Column (10 × 250 mm, 5 μm), a Waters XSelect CSH C18 column (10 × 250 mm, 5 μm), a NanoChrom ChromCore pentafluorophenyl column (10 × 250 mm, 5 μm), and an XTerra-OBD C8 column (10 × 250 mm, 5 μm). The UHPLC-ESI-TOF-MS analysis system consisted of an Agilent 1290 UHPLC system, an Agilent 6230 MS spectrometer, and a ZORBAX RRHD Eclipse Plus C18 column (1.8 μm, 2.1 × 50 mm, Agilent). In the enzyme activity experiment, the ultraviolet absorbance at 340 nm was collected using the Spectrummax Paradigm Multi-Mode Detection Platform.

### 3.2. Plant Material

The dried fruit of *A. kravanh* was purchased from Guangdong Kangmei Pharmaceutical Co., Ltd., Puning, China, in September 2021. The samples were kept in the State Key Laboratory of Quality Research in Chinese Medicines (Macau University of Science and Technology) after being identified by Prof. G.-Y. Zhu.

### 3.3. Extraction and Isolation

The dried fruits power of *A. kavanh* (10 kg) were extracted by 80% EtOH under reflux for 1 h each time (4 × 20 L). The solvent was removed under reduced pressure to obtain the crude extract (300 g), which was suspended in water and then partitioned successively with PE, EtOAc, and *n*-BuOH to obtain PE extract (80 g), EtOAc extract (57 g), and *n*-BuOH (17 g).

The PE extract was separated using silica gel CC eluted with the PE-EtOAc (from 100:0 to 1:1) to give eight fractions (Fr.P1-P8). Fr.P3 was chromatographed on the MCI column eluted with MeCN-H_2_O (70:30 to 100:0) to obtain **21** (2.8 mg) after recrystallization. Compound **16** (217 mg) was recrystallized from Fr.P6, and the residue was separated by MPLC with the C18 column eluted with MeCN/H_2_O (50:0 to 100:0) and then purified by semi-preparative HPLC eluted with MeOH/H_2_O (50:50) to obtain **18** (1 mg), **19** (0.4 mg), and **20** (1 mg). Fr.P7 was separated by MPLC eluted with MeCN/H_2_O (50:0 to 100:0) to obtain fractions Fr.P7-1~8. Fr.P7-3 was purified using semi-preparative HPLC eluted with MeOH/H_2_O (50:50) to afford **27** (1.2 mg), **28** (7 mg), and **29** (41.4 mg). Fr.P7-4 was subjected to semi-preparative HPLC eluted with MeCN/H_2_O (53:47) using a PFP column to give **2** (7.8 mg), **3** (0.4 mg), **6** (0.3 mg), and **30** (0.3 mg). Compounds **1** (1.3 mg), **4** (0.34 mg), **5** (2.6 mg), **7** (4 mg), **11** (3.4 mg), and **41** (1 mg) were obtained from Fr.P7-5 by repeated semi-preparative HPLC eluted with MeOH/H_2_O (70:30) and MeCN/H_2_O (53:47). Fr.P7-6 were repeatedly separated by semi-preparative HPLC eluted with MeCN/H_2_O (80:20) to afford **42** (11.6 mg), **43** (16 mg), **44** (17.8 mg), and **45** (10 mg). Compound **26** (7 mg) was isolated from Fr.P7-8 by semi-preparative HPLC eluted with MeCN/H_2_O (80:20). Fr.P8 was subjected to MPLC eluted with MeCN/H_2_O (50:0 to 100:0) to obtain eight subfractions (Fr.P8-1~8). Fr.P8-2 was repeatedly purified by HPLC eluted with MeCN/H_2_O (70:30) and MeOH/H_2_O (63:37) to give **8** (2.4 mg), **23** (4.3 mg), **24** (2.7 mg), and **35** (68 mg). Repeated purification of Fr. P8-3 by HPLC eluted with MeCN/H_2_O (70:30) and MeOH/H_2_O (63:37) to obtain **9** (1.7 mg) and **36** (47 mg). Fr.P8-8 was repeatedly purified by semi-preparative HPLC eluted with 90% MeCN in H_2_O and with MeOH/H_2_O (95:5) to obtain **12** (52 mg), **13** (8 mg), **14** (4 mg), **15** (1.2 mg), and **17** (10 mg).

The EtOAc extract (57g) was separated into 17 fractions (Fr.E1-E17) using an MCI column eluted with MeCN/H_2_O (30:70 to 100:0). Fr.E7 was purified by semi-preparative HPLC eluted with MeCN/H_2_O (35:65) to afford **10** (7.4 mg), **23** (4.3 mg), **32** (1 mg), **33** (12 mg), **34** (0.8 mg), **37** (2 mg), and **38** (1.6 mg). Compound **25** (1.3 mg) was recrystallized from Fr.E17.

The *n*-BuOH fraction (17 g) was separated into 11 fractions (Fr.B1-11) by an MCI column eluted with MeCN/H_2_O (10:90 to 100:0). Compound **40** (0.6 mg) was purified from Fr.B3 by HPLC eluted with MeCN/H_2_O (30:70). Compound **41** (1 mg) was obtained from Fr.B6 by HPLC eluted with MeCN/H_2_O (30:70).

### 3.4. Spectral and Physical Data of Compounds ***1**–**10***

Amomumol A (**1**): yellow oil. [*α*]_22.5_^D^ −20.5 (c = 0.5, MeOH); UV (MeOH) *λ*_max_ (log *ε*) 195 (0.85) nm; IR (KBr) *ν*_max_ 2926, 1685, 1452, 1369, and 1095 cm^−1^; ^1^H NMR (CDCl_3_, 600 MHz) and ^13^C NMR (CDCl_3_, 150 MHz) data are given in Table 1; HRESIMS *m*/*z* 237.1848 [M + H]^+^ (calculated for [C_15_H_24_O_2_ + H]^+^, 237.1849).

Amomumol B (**2**): yellow oil. [*α*]_22.5_^D^ −32.2 (c = 0.5 MeOH); UV (MeOH) *λ*_max_ (log *ε*) 195 (0.55) nm; IR (KBr) *ν*_max_ 3435, 2929, 1708, 1450, 1375, 1242, 1051, and 898 cm^−1^; ^1^H NMR (CDCl_3_, 600 MHz) and ^13^C NMR (CDCl_3_, 150 MHz) data are given in Table 1; HRESIMS *m*/*z* 235.1701 [M + H]^+^ (calculated for [C_15_H_22_O_2_ + H] ^+^, 235.1693).

Amomumol C (**3**): yellow oil. [*α*]_22.5_^D^ −61.5 (c = 0.5, MeOH); UV (MeOH) *λ*_max_ (log *ε*) 195 (2.01), 217 (0.90) nm; IR (KBr) *ν*_max_ 2924, 1707, 1458, 1211, 1043 cm^−1^; ^1^H NMR (CDCl_3_, 600 MHz) and ^13^C NMR (CDCl_3_, 150 MHz) data are given in Table 1; HRESIMS *m*/*z* 250.1810 [M + NH_4_]^+^ (calculated for [C_15_H_20_O_2_ + NH_4_]^+^, 250.1802).

Amomumol D (**4**): yellow oil. [*α*]_22.5_^D^ −46.1 (c = 0.5, MeOH); UV (MeOH) *λ*_max_ (log *ε*) 195 (0.95), 225 (0.55) nm; IR (KBr) *ν*_max_ 2926, 1732, 1714, 1666, 1454, 1043, 979 cm^−1^; ^1^H NMR (CDCl_3_, 600 MHz) and ^13^C NMR (CDCl_3_, 150 MHz) data are given in Table 1; HRESIMS *m*/*z* 235.1694 [M + H]^+^ (calculated for [C_15_H_22_O_2_ + H]^+^, 235.1693).

Amomumol E (**5**): yellow oil. [*α*]_22.5_^D^ −75.9 (c = 0.5, MeOH); UV (MeOH) *λ*_max_ (log *ε*) 195 (1.00), 228 (0.78) nm; IR (KBr) *ν*_max_ 2956, 1676, 1369, 1265, 1051 cm^−1^; ^1^H NMR (CDCl_3_, 600 MHz) and ^13^C NMR (CDCl_3_, 150 MHz) data are given in Table 2; HRESIMS *m*/*z* 321.2429 [M + H]^+^ (calculated for [C_20_H_32_O_3_ + H]^+^, 321.2424).

Amomumol F (**6**): yellow oil. [*α*]_22.5_^D^ −34.4 (c = 0.5, MeOH); UV (MeOH) *λ*_max_ (log *ε*) 195 (1.00), 228 (0.78) nm; IR (KBr) *ν*_max_ 2958, 1728, 1371, 1255, 1165, 983 cm^−1^; ^1^H NMR (CDCl_3_, 600 MHz) and ^13^C NMR (CDCl_3_, 150 MHz) data are given in Table 2; HRESIMS *m*/*z* 359.2184 [M + Na]^+^ (calculated for [C_20_H_32_O_4_ + Na]^+^, 359.2193).

Amomumol G (**7**): colorless oil. [*α*]_22.5_^D^ −122.4 (c = 0.5, MeOH); UV (MeOH) *λ*_max_ (log *ε*) 197 (2.13), 256 (1.02) nm; IR (KBr) *ν*_max_ 2978, 1718, 1458 cm^−1^; ^1^H NMR (CDCl_3_, 600 MHz) and ^13^C NMR (CDCl_3_, 150 MHz) data are given in Table 3; HRESIMS *m*/*z* 327.1568 [M + Na]_+_ (calculated for [C_18_H_24_O_2_ + Na]^+^, 327.1567).

Amomumol H (**8**): yellow powder. [*α*]_22.5_^D^ −21.1 (c = 0.5, MeOH); UV (MeOH) *λ*_max_ (log *ε*) 205 (1.05), 220 (0.80), 270 (0.50) nm; IR (KBr) *ν*_max_ 2958, 1581, 1500, 1458, 1419, 1328, 1219, 1122 cm^−1^; ^1^H NMR (CDCl_3_, 600 MHz) and ^13^C NMR (CDCl_3_, 150 MHz) data are given in Table 3; HRESIMS *m*/*z* 427.1735 [M + Na]^+^ (calculated for [C_22_H_28_O_7_ + Na]^+^, 427.1727).

Amomumol I (**9**): yellow powder. [*α*]_22.5_^D^ −30.8 (c = 0.5, MeOH); UV (MeOH) *λ*_max_ (log *ε*) 205 (1.05), 220 (0.80), 270 (0.50) nm; IR (KBr) *ν*_max_ 2958, 1724, 1375, 1240, 1028 cm^−1^; ^1^H NMR (MeOD, 600 MHz) and ^13^C NMR (MeOD, 150 MHz) data are given in Table 3. HRESIMS *m*/*z* 389.1961 [M + H]^+^ (calculated for [C_22_H_28_O_6_ + H]^+^, 389.1959).

Amomumol *J* (**10**): yellow oil. [*α*]_22.5_^D^ −25.1 (c = 0.5, MeOH); UV (MeOH) *λ*_max_ (log *ε*) 198 (0.95), 225 (0.55) nm; IR (KBr) *ν*_max_ 2958, 1737, 1714, 1371, 1240, 1029 cm^−1^; ^1^H NMR (CDCl_3_, 600 MHz) and ^13^C NMR (CDCl_3_, 150 MHz) data are given in Table 1; HRESIMS *m*/*z* 223.1697 [M + H]^+^ (calculated for [C_14_H_22_O_2_ + H]^+^, 223.1697).

Amomumol *J* derivative (**10a**): yellow oil. ^1^H NMR (MeOD, 600 MHz) and ^13^C NMR (MeOD, 150 MHz) data are given in Table S1; HRESIMS *m*/*z* 265.1801 [M + H]^+^ (calculated for [C_14_H_24_O_3_ + H]^+^, 265.1798).

### 3.5. Acetylation Reaction of Compound ***10***

This acetylation reaction used the traditional pyridine-acetic anhydride method to acylate the acetyl group to the OH-10 of **10**. Compound **10** (4 mg) was dissolved in 150 μL of pyridine, and then 50 μL of acetic anhydride was added to react at room temperature for 3 h. The solvent was recovered to obtain compound **10a** (4.5 mg).

### 3.6. Assay for ADH-Promoting Activity In Vitro

A modified traditional Valle–Hoch method was used to measure ADH activity [43]. Briefly, 10 μL of 27 mM NAD^+^ solution, 10 μL of ADH (0.1 mg/mL) solution, and 10 μL of different concentrations of test compounds (metadoxine as a positive control) were added into 60 μL of 32 mM sodium pyrophosphate buffer (pH 8.8). The negative control group used 10 μL buffer instead of the sample solution. After incubating at room temperature for 30 min, 10 μL of 5% alcohol was added to the mixture for 5 min. The absorbances at 340 nm wavelength were then taken using a microplate reader.

## 4. Conclusions

To identify the bioactive compounds from *A. kravanh*, we used in vitro assay to evaluate the ADH-promoting activity of the PE, EtOAc, and *n*-BuOH fractions of the extract of the fruits of *A. kravanh* and found that the PE and EtOAc fractions showed ADH enhancing activity. The following phytochemical investigation resulted in the isolation of four new sesquiterpenoids (**1**–**4**), three new monoterpene derivatives (**5**–**7**), two new neolignans (**8** and **9**), a novel norsesquiterpenoid (**10**), and thirty-five known compounds. The bioassay results showed that compounds **11**, **12**, **15**, **18**, **26**, and **36**–**38** significantly enhanced alcohol dehydrogenase activity in a dose-dependent manner. These results give a new insight into the chemical diversity and the potential usage in the hangover cure of *A. kravanh.*

## Figures and Tables

**Table 1:** ^1^H-NMR (600 MHz) and ^13^C-NMR (150 MHz) data of compounds **1**–**4** and **10** (*δ* in ppm, *J* in Hz).

No.	1 (CDCl_3_)		2 (CDCl_3_)		3 (CDCl_3_)		4 (CDCl_3_)		10 (MeOD)	
	*δ*_C_	*δ*_H_	*δ*_C_	*δ*_H_	*δ*_C_	*δ*_H_	*δ*_C_	*δ*_H_	*δ*_C_	*δ*_H_
1	151.8		135.4		138.6		202.8		202.8	
2	69.8	4.46, dd, 4.9, 11.5	199.7		127.2	7.18, d, 7.9	150.4		129.9	6.00, d, 10.0
3	29.5	1.97, m	31.5	2.59, dd, 1.44, 3.92	127.3	7.3, d, 7.9	29.3	2.84, ddt, 2.8, 5.8, 13.0	157.0	7.03, brd, 10.0
		1.42, m		2.36, dd, 13.2, 15.9				2.17, td, 2.8, 13.0		
4	84.1		40.8	2.7, m	146.2		35.4	1.44, m	42.5	2.54, m
5	40.7	1.54, m	43.3	2.47, 2.28	127.3	7.3, d, 7.9	70.8	3.92, d, 8.1	25.0	2.01, m
		1.27, m							(25.1)	1.79, m
6	46.8	2.43, dt, 3.3, 13.6	140.3	6.74, brd, 4.5	127.3	7.18, d, 7.9	151.8		38.2	2.49, brt, 16.5
		2.28, ddd, 2.7, 4.3, 13.3								2.41, dt, 4.2, 16.5
7	43.6	1.99, m	149.5		39.2	2.72, m	41.5	2.87, ddd, 1.2, 4.8, 13.1	37.7	1.71, m
								2.74, brt, 11.6	(37.8)	
8	33.5	2.10, m	37.3	2.79, d, 6.6	32.7	1.93, m	124.8	5.06, ddd, 4.8, 11.6, 15.4	31.0	1.42, m
		1.34, m				1.86, m			(31.1)	1.32, m
9	73.9	4.66, ddd, 5.9, 8.7, 10.0	124.3	5.61, dt, 6.6, 15.4	35.5	2.85, m	140.1	5.38, dd, 15.4	33.9	1.63, m
						2.50, m			(33.9)	1.54, m
10	127.4	5.16, brd, 1.5, 8.5	144.5	5.65, d, 15.4	202.0		37.3		76.8	4.02, t, 6.4
									(77.0)	
11	134.6		70.6		144.4		47.9	3.20, d, 11.0;	148.7	
								2.01, d, 4.5	(148.8)	
12	18.1	1.68, q, 1.2	29.8	1.33, s	124.4	5.81, s	124.3	5.84, s	111.4	4.94, s
						5.69, s		5.70, s	(111.6)	4.83, s
13	25.8	1.72, q, 1.2	29.8	1.33, s	17.6	1.83, s	109.2	5.10, s	17.6	1.73, s
								4.90, s	(17.7)	
14	14.3	0.93, d, 7.0	110.6	4.86, s	22.5	1.27, s	23.6	1.15, s	16.5	0.94, d, 6.8
									(16.5)	
15	103.2	4.78, q, 1.5;	15.6	1.79, s	65.2	4.68, s	31.6	1.09, s		
		4.92, q, 1.5								

**Table 2:** ^1^H-NMR (600 MHz) and ^13^C-NMR (150 MHz) data of compound **5**–**7**.

No.	5 (CDCl_3_)		6 (CDCl_3_)		7 (CDCl_3_)	
	*δ*_C_	*δ*_H_	*δ*_C_	*δ*_H_	*δ*_C_	*δ*_H_
1	200.0		67.5	4.00, t, 3	67.7	4.08, d, 3
2	135.3		139.2		138.5	
3	145.0	6.73, s	124.7	5.37, s	125.6	5.57, s
4	74.4	3.98, d, 9.4	71.9	5.16, brd, 8.6	71.9	5.44, brd, 9.0
5	47.7	2.03, m	38.9	1.76, m	39.0	1.98, m
6	35.7	2.45, dd, 3.2, 15.5	30.0	1.79, 1.56, m	30.4	1.89, 1.65, m
		2.11, d, 15.2				
7	25.1	2.27, dm, 3.2, 6.9	26.6	1.72, m	26.8	1.85, m
8	16.5	0.87, d, 6.9	20.6	0.92, d, 6.9	17.9	0.87, d, 6.9
9	20.7	0.91, d, 6.9	17.3	0.79, d, 6.9	20.3	0.96, d, 6.9
10	20.6	1.83, s	20.3	1.79, s	20.5	1.83, s
1′	73.1	3.77, brs	172.0		166.6	
2′	134.3		37.2	2.56, dd, 4.7, 14.8	122.8	
				2.40, dd, 9.5, 14.8		
3′	131.2	5.63, s	45.5	2.6, m, 4.7	131.6	8.01, d, 9.0
4′	69.3	3.92, d, 9.14	148.0	6.65, dd, 8.91, 16.0	113.6	6.93, d, 9.0
5′	42.7	1.61, m	132.3	6.09, d, 16.0	163.3	
6′	25.4	1.83,1.29, m	198.1		113.6	6.93, d, 9.0
7′	26.3	2.15, dm,2.7,6.9	27.1	2.24, s	131.6	8.01, d, 9.0
8′	16.8	0.85, d, 6.9	31.5	1.76, m	55.4	3.86, s
9′	20.9	0.98, d, 6.9	20.2	0.94, d, 6.1		
10′	15.6	1.81, s	19.3	0.89, d, 6.1		

**Table 3:** ^1^H-NMR (600 MHz) and ^13^C-NMR (150 MHz) data of compounds **8** and **9**.

No.	8 (MEOD)		9 (CDCl_3_)	
	*δ*_C_	*δ*_H_	*δ*_C_	*δ*_H_
1	135.5		132.9	
2	149.3		146.7	
3	104.7	6.60, s	106.7	6.45, s
4	135.5		130.1	
5	104.7	6.60, s	106.7	6.45, s
6	149.3		146.7	
7	75.8	4.79, d, 3.6	43.4	3.10, dd, 13.4, 5.1
				2.71, dd, 13.4, 8.1
8	83.8	4.32, dq, 3.6, 6.3	80.3	4.35, m
9	13.9	1.15, d, 6.3	20.4	1.21, d, 6.2
1′	135.5		135.2	
2′	154.9		153.7	
3′	104.2	6.67, s	103.0	6.56, s
4′	135.5		133.4	
5′	104.2	6.67, s	103.0	6.56, s
6′	154.9		153.7	
7′	132.3	6.36, d, 15.7	130.9	6.32, d, 15.7
8′	126.2	6.24, dq, 6.5, 15.7	125.1	6.17, dq, 6.5, 15.7
9′	18.4	1.88, d, 6.5	18.6	1.88, d, 6.5
2,6-OCH_3_	56.7	3.80, s	56.3	3.86, s
2′,6′-OCH_3_	56.5	3.85, s	56.0	3.81, s

## Data Availability

Data of the compounds are available in Supplementary Materials.

## Supplementary Materials

The following supporting information can be downloaded at https://www.mdpi.com/article/10.3390/molecules28124878/s1. Figures S1–S99: MS, IR, UV, ^1^H-NMR, ^13^C-NMR, and 2D NMR spectra of compounds **1–10**; Figures S100–S102: MS, ^1^H and ^13^C NMR spectra of compound **10a**. Table S1: NMR data of compound **10a**; Figure S103: The effects of PE, EtOAc, and *n*-BuOH extracts on ADH activity.

## Sample Availability

Not all samples of the compounds (**1**–**45**) are available from the authors since some compounds run out after their bioassay.

## References

[1] Aslam A., Kwo P.Y. (2023). Epidemiology and disease burden of alcohol associated liver disease. J. Clin. Exp. Hepatol..

[2] Hyun J.Y., Kim S.K., Yoon S.J., Lee S.B., Jeong J.J., Gupta H., Sharma S.P., Oh K.K., Won S.M., Kwon G.H. (2022). Microbiome-based metabolic therapeutic approaches in alcoholic liver disease. Int. J. Mol. Sci..

[3] Szabo G. (2015). Gut-liver axis in alcoholic liver disease. Gastroenterology.

[4] Rehm J. (2017). The relationship between different dimensions of alcohol use and the burden of disease-an update. Addiction.

[5] Stockwell T. (2017). Alcohol’s contribution to cancer is underestimated for exactly the same reason that its contribution to cardioprotection is overestimated. Addiction.

[6] Evangelou E., Suzuki H., Bai W., Pazoki R., Gao H., Matthews P.M., Elliott P. (2021). Alcohol consumption in the general population is associated with structural changes in multiple organ systems. Elife.

[7] Ayares G., Idalsoaga F., Díaz L.A., Arnold J., Arab J.P. (2022). Current medical treatment for alcohol-associated liver disease. J. Clin. Exp. Hepatol..

[8] Wen D.C., Hu X.Y., Wang Y.Y., Luo J.X., Lin W., Jia L.Y., Gong X.Y. (2016). Effects of aqueous extracts from *Panax ginseng* and hippophae rhamnoides on acute alcohol intoxication: An experimental study using mouse model. J. Ethnopharmacol..

[9] Liang J., Olsen R.W. (2014). Alcohol use disorders and current pharmacological therapies: The role of GABAA receptors. Acta Pharmacol. Sin..

[10] Yin H., Luo J.G., Kong L.Y. (2013). Tetracyclic diterpenoids with isomerized isospongian skeleton and labdane diterpenoids from the fruits of *Amomum kravanh*. J. Nat. Prod..

[11] Zhang J.S., Cao X.X., Zhang H. (2020). Chemical constituents from the fruits of *Amomum kravanh*. Biochem. Syst. Ecol..

[12] Zhang J.S., Cao X.X., Yu J.H., Yu Z.P., Zhang H. (2020). Diarylheptanoids with NO production inhibitory activity from *Amomum kravanh*. Bioorg. Med. Chem. Lett..

[13] Mathew J., Shiburaj S., George V. (2003). Antimicrobial activity of *Amomum cannicarpum*. Fitoterapia.

[14] Yang Y., Yan R., Zou G. (2010). Cytotoxic, apoptotic and antioxidant activity of the essential oil of *Amomum tsao-ko*. Bioresour. Technol..

[15] Patanasethanot D., Nagai J., Yumoto R., Murakami T., Sutthanut K., Sripanidkulchai B.O., Yenjai C., Takano M. (2007). Effects of *Kaempferia parviflora* extracts and their flavone constituents on P-glycoprotein function. J. Pharm. Sci..

[16] Sookkongwaree K., Geitmann M., Roengsumran S., Petsom A., Danielson U.H. (2006). Inhibition of viral proteases by Zingiberaceae extracts and flavones isolated from *Kaempferia parviflora*. Pharmazie.

[17] JiSuk L., Kyoung A.K., SeonHui J., SungGeum L., Hi J.P., Nam J.K., Sabina L. (2009). Anti-inflammatory, anti-nociceptive, and anti-psychiatric effects by the rhizomes of *Alpinia officinarum* on complete freund’s adjuvant-induced arthritis in rats. J. Ethnopharmacol..

[18] Borchuluun S., Wang Q., Xu Y., He X., Bao W., Pa B. (2021). Structure elucidation and NMR assignments of a new sesquiterpene of volatile oil from *Artemisia frigida* Willd. Nat. Prod. Res..

[19] Gutierrez A.B., Herz W. (1988). Bisabolones and other constituents of *Mikania shushunensis*. Phytochemistry.

[20] Takeda S., Iimura Y., Tanaka K., Kurosawa E., Suzuki T. (1990). A new naturally occurring racemic compound from the marine red alga laurencia obtusa (hudson) lamouroux. Chem. Lett..

[21] Cuenca M., Catalan C., Díaz J.G., Herz W. (1991). Monoterpenes and lignans from *Mikania saltensis*. J. Nat. Prod..

[22] Bohlmann F., Kramp W., Gupta R.K., King R.M., Robinson H. (1981). Four guaianolides and other constituents from three *kaunia* species. Phytochemistry.

[23] Liu S.S., Sheng W.L., Li Y., Zhang S.S., Zhang M. (2019). Chemical constituents from *Alismatis Rhizoma* and their anti-inflammatory activities in vitro and in vivo. Bio. Chem..

[24] Rédei D., Kúsz N., Rafai T., Bogdanov A., Burián K., Csorba A., Mándi A., Kurtán T., Vasas A., Hohmann J. (2019). 14-Noreudesmanes and a phenylpropane heterodimer from sea buckthorn berry inhibit Herpes simplex type 2 virus replication. Tetrahedron.

[25] Nuñez Y.O., Salabarria I.S., Collado I.G., Hernández-Galán R. (2007). Sesquiterpenes from the wood of *Juniperus lucayana*. Phytochemistry.

[26] Alexander-Lindo R.L., Morrison E.Y., Nair M.G. (2004). Hypoglycaemic effect of stigmast-4-en-3-one and its corresponding alcohol from the bark of *Anacardium occidentale* (cashew). Phytother. Res..

[27] Suguru T., Chiharu G., Yasuhisa N., Takahiro N., Shozo F. (2009). Synthesis of some C_28_ steroids with C-24 (28) double bonds. J. Jpn. Oil Chem. Soc..

[28] Huo J., Tang H., Li L., Liu B.S., Zhang W. (2011). Study on bioactive constituents of the south China sea soft coral *Scleronephthya* sp. Acad. J. Second Mil. Med. Univ..

[29] Anne G., Jacqueline S., Maurice A. (2000). Isolation of bioactive 5α,8α-epidioxy sterols from the marine sponge *Luffariella* cf. variabilis. Can. J. Chem..

[30] Yaoita Y., Amemiya K., Ohnuma H., Furumura K., Masaki A., Matsuki T., Kikuchi M. (1998). Cheminform abstract: Constituents of mushrooms. Part 3. sterol constituents from five edible mushrooms. Chem. Pharm. Bull..

[31] Haven H., Rullk-Tter J. (1988). The diagenetic fate of taraxer-14-ene and oleanene isomers. Geochim. Cosmochim. Acta.

[32] Parmar V.S., Vardhan A., Nagarajan G.R., Jain R. (1992). Dihydroflavonols from *Prunus Domestica*. Phytochemistry.

[33] Afifi F.Ü., Al-Khalil S., Abdul-Haq B.K., Mahasneh A. (1991). Antifungal flavonoids from *Varthemia iphionoides*. Phyto. Res..

[34] Yenjai C., Prasanphen K., Daodee S., Wongpanich V., Kittakoop P. (2004). Bioactive flavonoids from *Kaempferia parviflora*. Fitoterapia.

[35] Lin R.J., Lo W.L., Wang Y.D., Chen C.Y. (2008). A novel cytotoxic monoterpenoid from the leaves of *Cinnamomum subavenium*. Nat. Prod. Res..

[36] Versini G., Rapp A., Reniero F., Mandery H. (1991). Structural identification and presence of some *p*-menth-1-enediols in grape products. Vitis.

[37] Hoskins J.A. (1984). The occurrence, metabolism and toxicity of cinnamic acid and related compounds. J. Appl. Toxicol..

[38] Wahidullah S., Desouza L., Kamat S.Y. (1991). Dipeptides from the red alga *Acanthopora spicifera*. Phytochemistry.

[39] Tan S.B., Guo T., Tang X.F., Song T.T., Wang Y. (2018). Chemical constituents of *Zanthoxylum armatum*. II. Chem. Nat. Compd..

[40] Ouyang M.A., Wein Y.S., Su R.K., Kuo Y.H. (2007). Rhusemialins A-C, new cyclolignan esters from the roots of *Rhus javanica var. roxburghiana*. Chem. Pharm. Bull..

[41] Ai W., Wei X., Lin X., Sheng L., Wang Z., Tu Z., Yang X., Zhou X., Li J., Liu Y. (2014). Guignardins A–F, spirodioxynaphthalenes from the endophytic fungus *Guignardia* sp. KcF8 as a new class of PTP1B and SIRT1 inhibitors. Tetrahedron.

[42] Vollenweider S., Weber H., Stolz S., Chételat A., Farmer E.E. (2000). Fatty acid ketodienes and fatty acid ketotrienes: Michael addition acceptors that accumulate in wounded and diseased *Arabidopsis* leaves. Plant J..

[43] Shi Y., Yu F., Wu Y., Dai L., Feng Y., Chen S., Wang G., Ma H., Li X., Dai C. (2022). Identification of a novel peptide that activates alcohol dehydrogenase from crucian carp swim bladder and how it protects against acute alcohol-induced liver injury in mice. J. Pharm. Biomed. Anal..

