# Supplementary material for: Chemical Constituents from the Fruits of Amomum kravanh and Their Role in Activating Alcohol Dehydrogenase
Source: Molecules. 2023 Jun 20;28(12):4878. doi: 10.3390/molecules28124878 (PMC10303173; doi:10.3390/molecules28124878)
Supplement: Supplementary file 1 [file molecules-28-04878-s001.zip › molecules-2425797-supplementary.pdf]

Supplementary Materials

Chemical constituents from the fruits of *Amomum kravanh* and their role in activating alcohol dehydrogenase

Hao-Ming Xiong, Hui-Ying Li, Zhi-Rong Lin, Xiao-Mei Liu, Li-Ping Bai, Wei Zhang, Zhi-Hong Jiang,* and Guo-Yuan Zhu*

State Key Laboratory of Quality Research in Chinese Medicine, Guangdong-Hong Kong-Macao Joint Laboratory of Respiratory Infectious Disease, Macau Institute for Applied Research in Medicine and Health, Macau University of Science and Technology, Macau 999078, China

* Correspondence: zhjiang@must.edu.mo (Z.-H. J.); gyzhu@must.edu.mo (G.-Y. Z.)

Table of Contents

Figure S1. The HRESIMS spectrum of compound **1** **S5**
Figure S2. The ^{1}H NMR spectrum of compound **1** **S5**
Figure S3. The ^{13}CNMR spectra of compound **1** **S6**
Figure S4. The DEPT135 spectra of compound **1** **S6**
Figure S5. The HSQC spectrum of compound **1** **S7**
Figure S6. The ^{1}H-^{1}H COSY spectrum of compound 1 .... **S7**
Figure S7. The HMBC spectrum of compound **1** **S8**
Figure S8. The NOESY spectrum of compound **1** **S8**
Figure S9. The IR spectrum of compound **1** **S9**
Figure S10. The UV spectrum of compound **1** **S9**
Figure S11. The HRESIMS spectrum of compound **2** **S10**
Figure S12. The ^{1}H NMR spectrum of compound **2** **S10**
Figure S13. The ^{13}CNMR spectra of compound **2** **S11**
Figure S14. The DEPT135 spectra of compound **2** **S11**
Figure S15. The HSQC spectrum of compound **2** **S12**
Figure S16. The ^{1}H-^{1}H COSY spectrum of compound **2** **S12**
Figure S17. The HMBC spectrum of compound **2** **S13**
Figure S18. The NOESY spectrum of compound **2**........ **S13**
Figure S19. The IR spectrum of compound **2** **S14**
Figure S20. The UV spectrum of compound **2** **S14**
Figure S21. The HRESIMS spectrum of compound **3**........ **S15**
Figure S22. The ^{1}H NMR spectrum of compound **3** **S15**
Figure S23. The ^{13}CNMR spectra of compound **3** **S16**
Figure S24. The DEPT135 spectra of compound **3** **S16**
Figure S25. The HSQC spectrum of compound **3** **S17**
Figure S26. The ^{1}H-^{1}H COSY spectrum of compound **3** **S17**
Figure S27. The HMBC spectrum of compound **3** **S18**
Figure S28. The NOESY spectrum of compound **3** **S18**
Figure S29. The IR spectrum of compound **3** **S19**
Figure S30. The UV spectrum of compound **3** **S19**
Figure S31. The HRESIMS spectrum of compound **4** **S20**
Figure S32. The ^{1}H NMR spectrum of compound **4** **S20**
Figure S33. The ^{13}CNMR spectra of compound **4** **S21**
Figure S34. The DEPT135 spectra of compound **4** **S21**
Figure S35. The HSQC spectrum of compound **4** **S22**
Figure S36. The ^{1}H-^{1}H COSY spectrum of compound **4** **S22**
Figure S37. The HMBC spectrum of compound **4** **S23**
Figure S38. The NOESY spectrum of compound **4** **S23**
Figure S39. The IR spectrum of compound **4** **S24**
Figure S40. The UV spectrum of compound **4** **S24**
Figure S41. The HRESIMS spectrum of compound **5** **S25**
Figure S42. The ^{1}H NMR spectrum of compound **5** **S25**
Figure S43. The ^{13}CNMR spectra of compound **5** **S26**

Figure S44. The DEPT135 spectra of compound **5** **S26**
Figure S45. The HSQC spectrum of compound **5** **S27**
Figure S46. The ^{1}H-^{1}H COSY spectrum of compound **5** **S27**
Figure S47. The HMBC spectrum of compound **5** **S28**
Figure S48. The NOESY spectrum of compound **5** **S28**
Figure S49. The IR spectrum of compound **5**.......... **S29**
Figure S50. The UV spectrum of compound **5** **S29**
Figure S51. The HRESIMS spectrum of compound **6** **S30**
Figure S52. The ^{1}H NMR spectrum of compound **6** **S30**
Figure S53. The ^{13}CNMR spectra of compound **6** **S31**
Figure S54. The DEPT135 spectra of compound **6** **S31**
Figure S55. The HSQC spectrum of compound **6** **S32**
Figure S56. The ^{1}H-^{1}H COSY spectrum of compound **6** **S32**
Figure S57. The HMBC spectrum of compound **6** **S33**
Figure S58. The NOESY spectrum of compound **6** **S33**
Figure S59. The IR spectrum of compound **6** **S34**
Figure S60. The UV spectrum of compound **6** **S34**
Figure S61. The HRESIMS spectrum of compound **7** **S35**
Figure S62. The ^{1}H NMR spectrum of compound **7** **S35**
Figure S63. The ^{13}CNMR spectra of compound **7** **S36**
Figure S64. The DEPT135 spectra of compound **7** **S36**
Figure S65. The HSQC spectrum of compound **7** **S37**
Figure S66. The ^{1}H-^{1}H COSY spectrum of compound **7** **S37**
Figure S67. The HMBC spectrum of compound **7** **S38**
Figure S68. The NOESY spectrum of compound **7** **S38**
Figure S69. The IR spectrum of compound **7** **S39**
Figure S70. The UV spectrum of compound **7** **S39**
Figure S71. The HRESIMS spectrum of compound **8** **S40**
Figure S72. The ^{1}H NMR spectrum of compound **8** **S40**
Figure S73. The ^{13}CNMR spectra of compound **8** **S41**
Figure S74. The DEPT135 spectra of compound **8** **S41**
Figure S75. The HSQC spectrum of compound **8** **S42**
Figure S76. The ^{1}H-^{1}H COSY spectrum of compound **8** **S42**
Figure S77. The HMBC spectrum of compound **8** **S43**
Figure S78. The IR spectrum of compound **8** **S43**
Figure S79. The UV spectrum of compound **8** **S44**
Figure S80. The HRESIMS spectrum of compound **9** **S44**
Figure S81. The ^{1}H NMR spectrum of compound **9** **S45**
Figure S82. The ^{13}CNMR spectra of compound **9** **S45**
Figure S83. The DEPT135 spectra of compound **9** **S46**
Figure S84. The HSQC spectrum of compound **9** **S46**
Figure S85 The ^{1}H-^{1}H COSY spectrum of compound **9** **S47**
Figure S86. The HMBC spectrum of compound **9** **S47**
Figure S87. The NOESY spectrum of compound **9** **S48**
Figure S88. The IR spectrum of compound **9** **S48**
Figure S89. The UV spectrum of compound **9** **S49**
Figure S90. The HRESIMS spectrum of compound **10** **S49**

Figure S91. The ^{1}H NMR spectrum of compound **10** S**50**
Figure S92. The ^{13}CNMR spectra of compound **10** S**50**
Figure S93. The DEPT135 spectra of compound **10** S**51**
Figure S94. The HSQC spectrum of compound **10** S**51**
Figure S95. The ^{1}H-^{1}H COSY spectrum of compound **10** S**52**
Figure S96. The HMBC spectrum of compound **10** S**52**
Figure S97. The NOESY spectrum of compound **10** S**53**
Figure S98. The IR spectrum of compound **10** S**53**
Figure S99. The UV spectrum of compound **10** S**54**
Figure S100. The HRESIMS spectrum of compound **10** S**54**
Figure S101. The ^{1}H NMR spectrum of compound **10** S**55**
Figure S102. The ^{13}CNMR spectra of compound **10** S**55**
Table S1. ^{1}H-NMR (600 MHz) and 13C-NMR (150 MHz) data of compound **10a**.................. **S56**
Figure S103. The effects of PE, EtOAc, and n-BuOH extracts on ADH activity **S56**

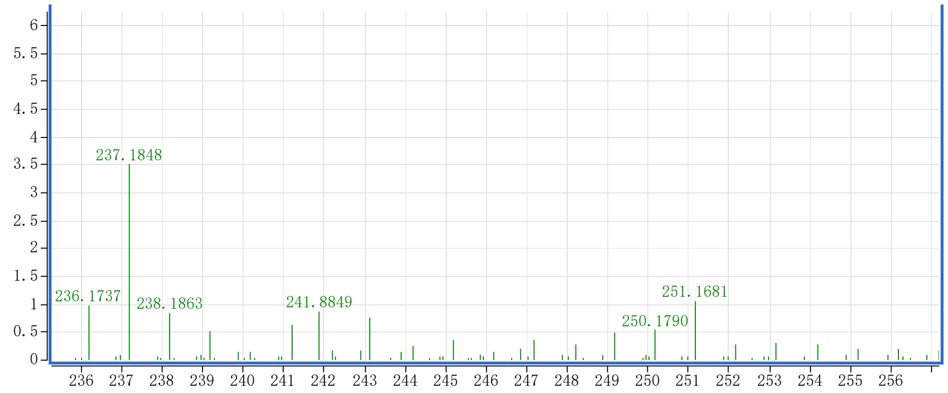

Formula	Score	Mass	Mass (MFG)	m/z (Calc)	Diff (ppm)	Ion Formula	m/z
$C_{15}H_{24}O_2$	99.95	236.1775	236.1776	237.1849	0.45	$C_{15}H_{25}O_2$	237.1848

Figure S1. The HRESIMS spectrum of compound **1**

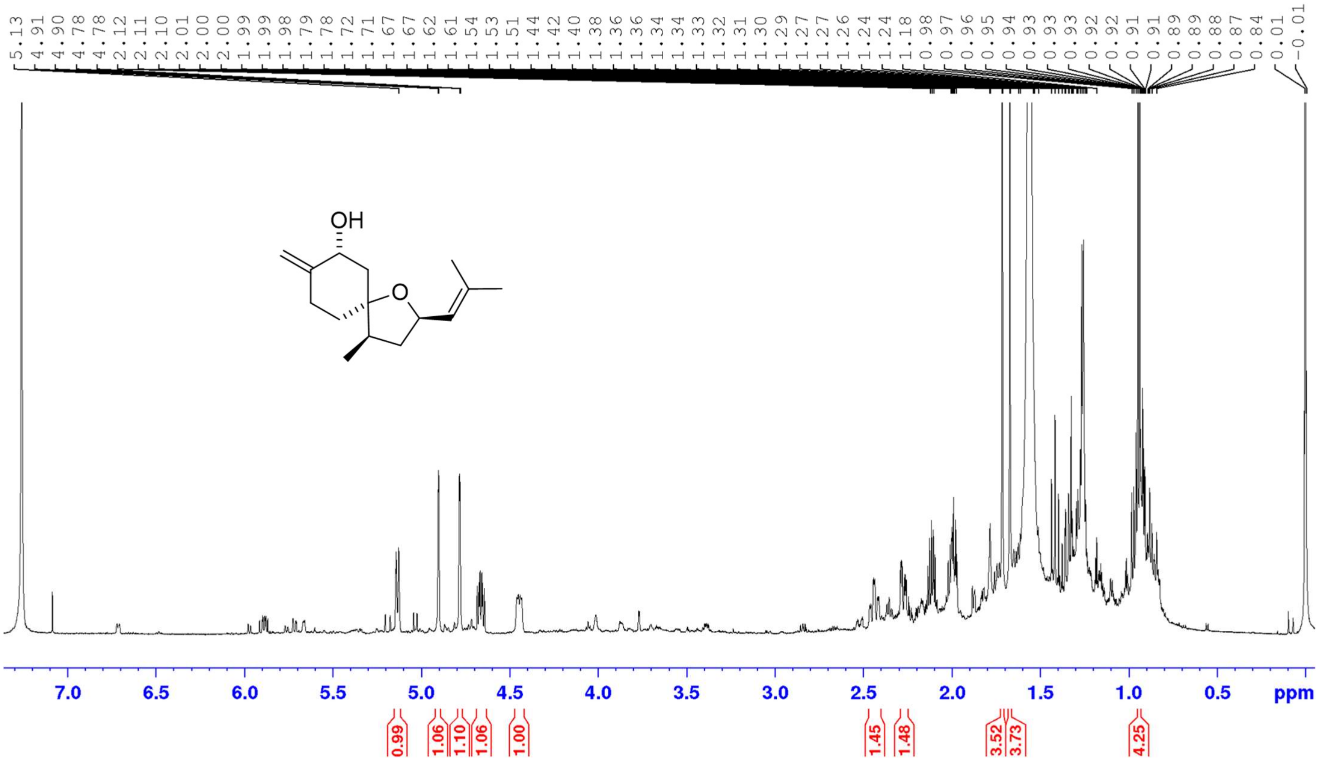

Figure S2. The ^{1}H NMR spectrum of compound **1**

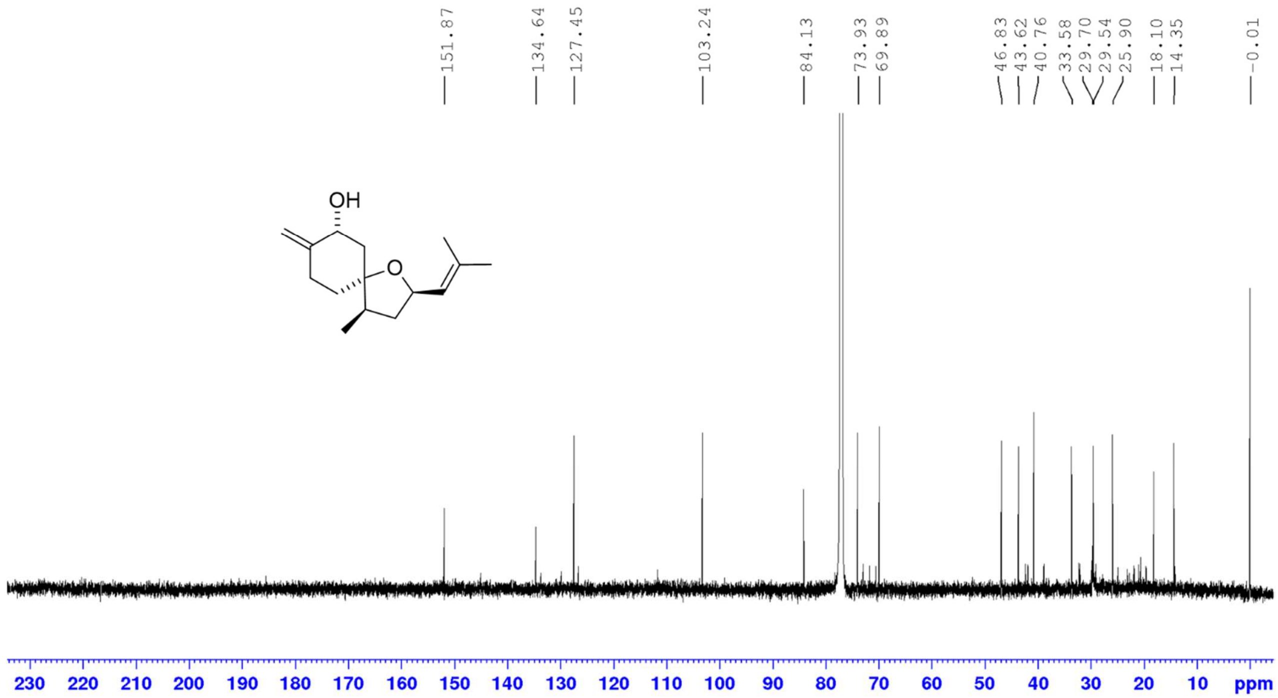

Figure S3. The ^{13}CNMR spectrum of compound **1**

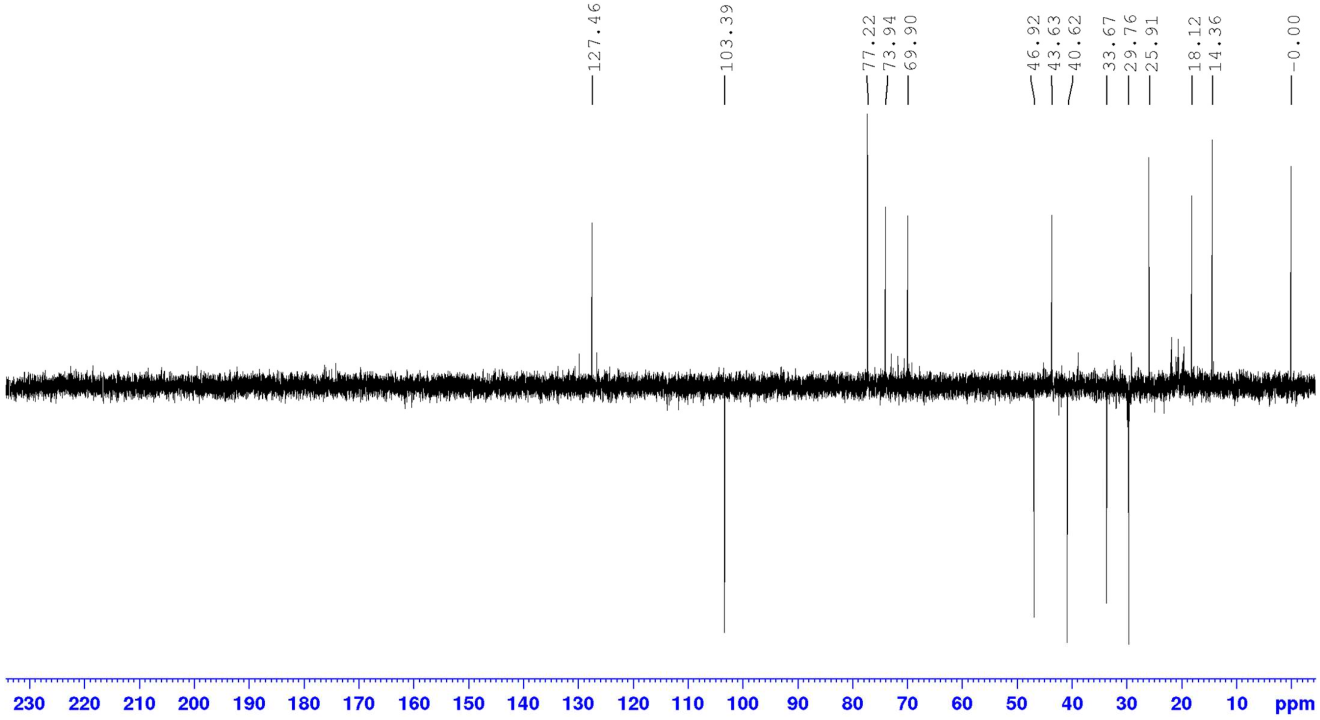

Figure S4. The DEPT135 spectrum of compound **1**

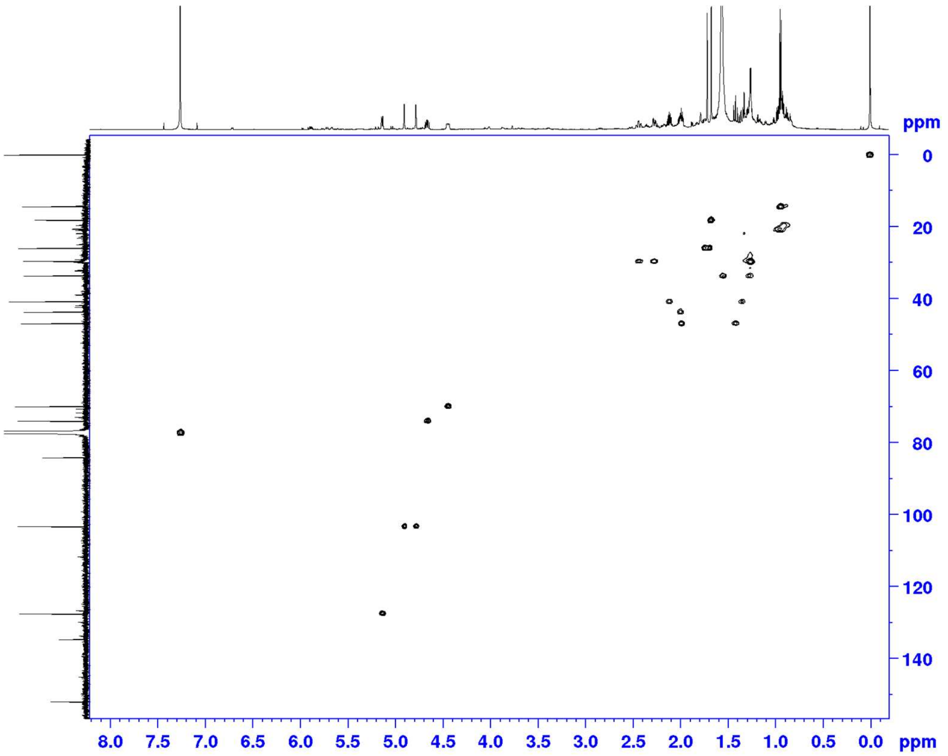

Figure S5. The HSQC spectrum of compound **1**

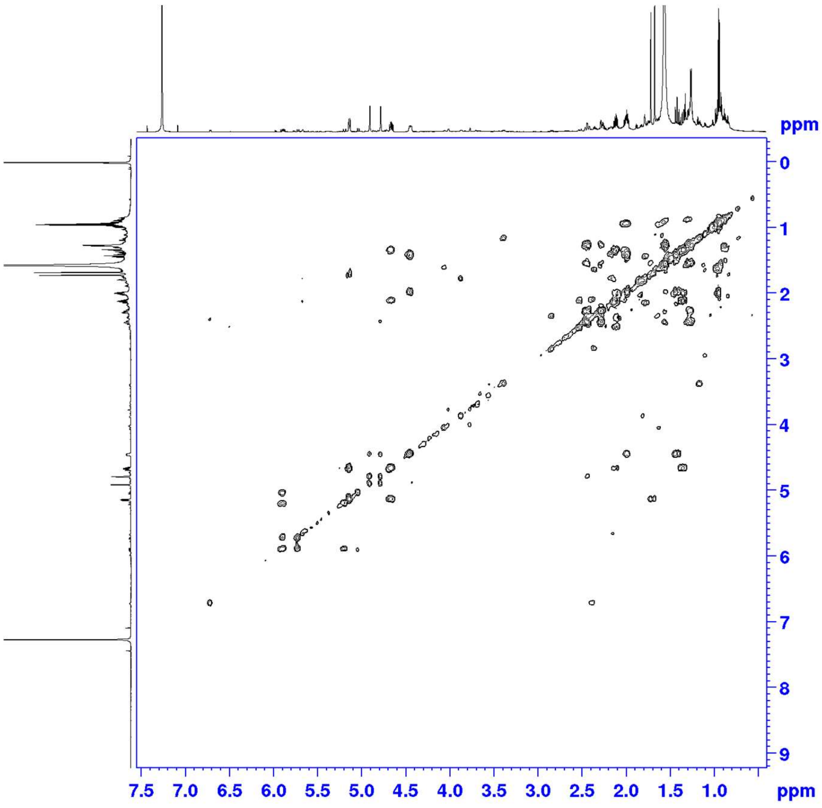

Figure S6. The ^{1}H-^{1}H COSY spectrum of compound **1**

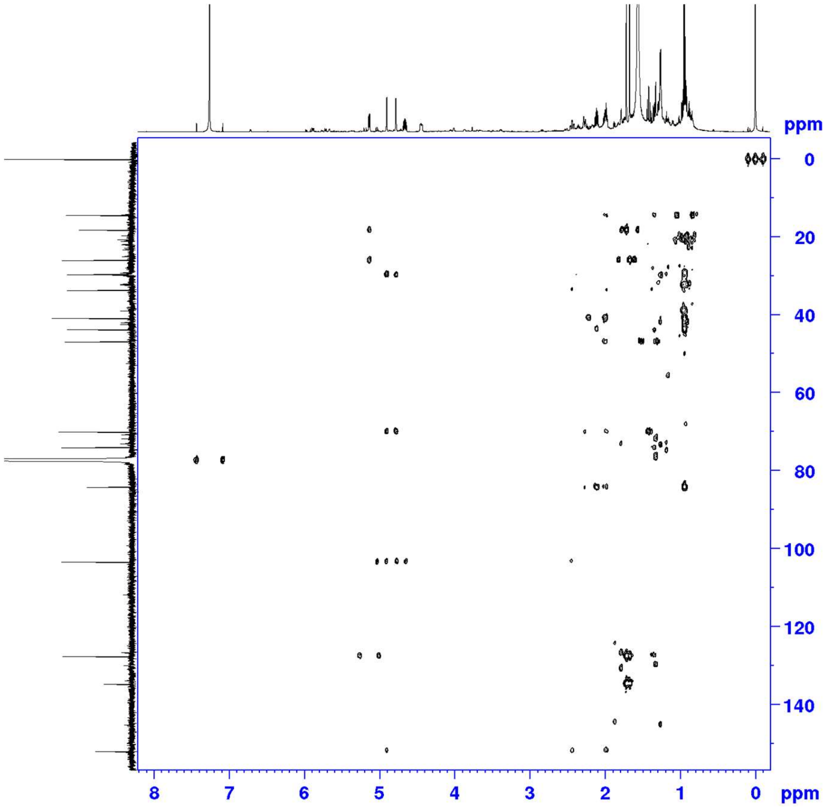

Figure S7. The HMBC spectrum of compound **1**

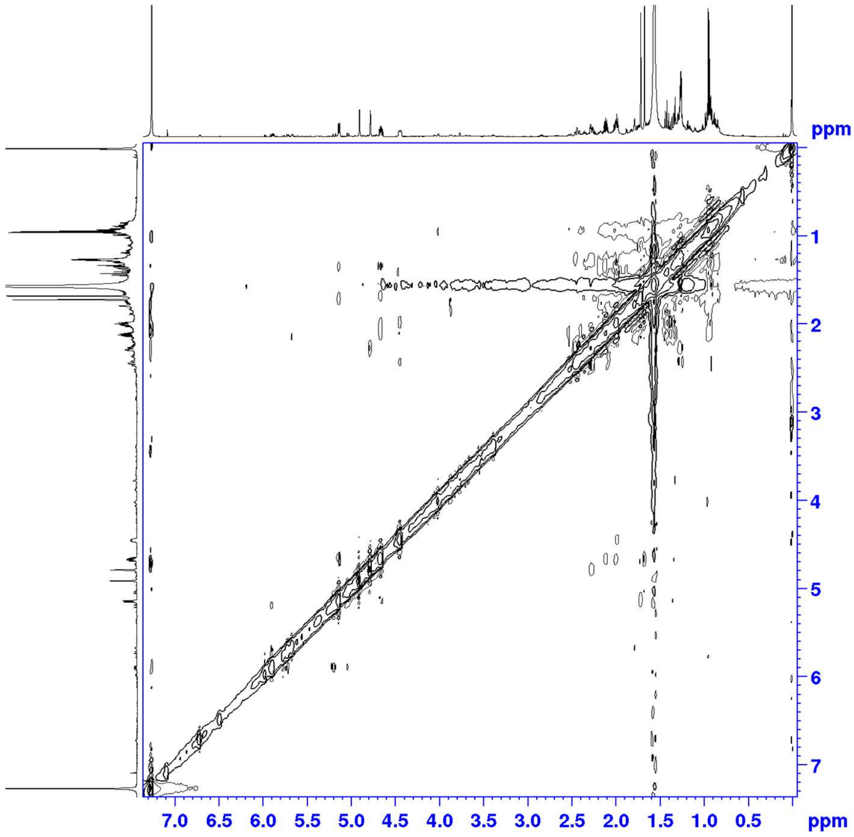

Figure S8. The NOESY spectrum of compound **1**

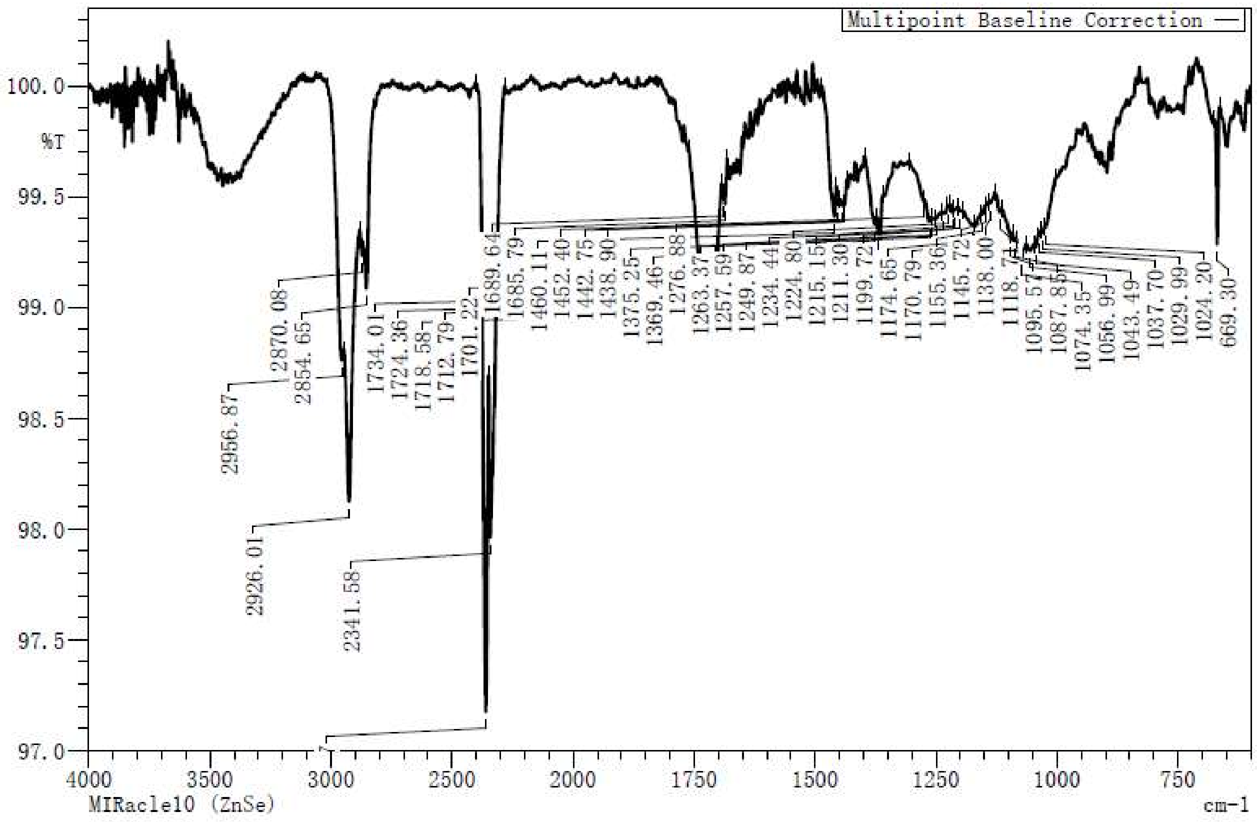

Figure S9. The IR spectrum of compound **1**

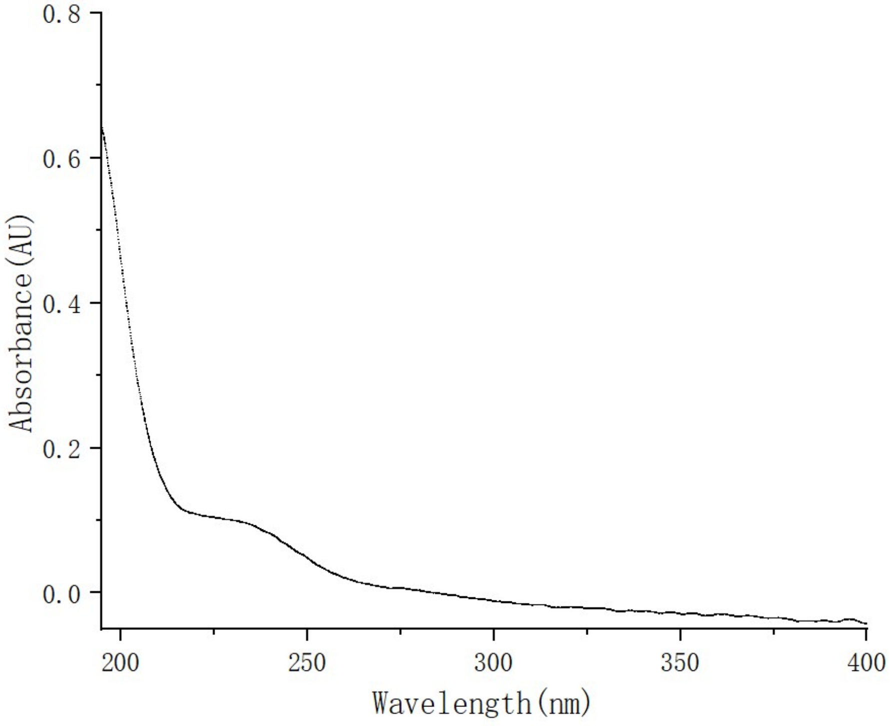

Figure S10. The UV spectrum of compound **1**

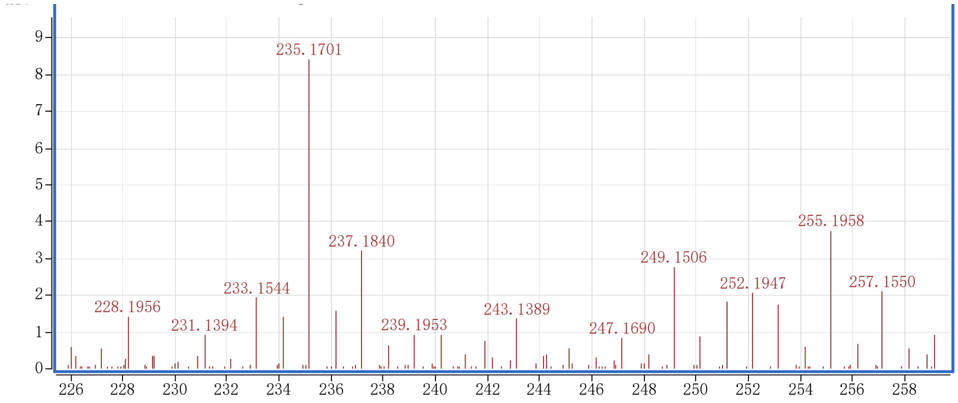

Formula	Score	Mass	Mass (MFG)	m/z (Calc)	Diff (ppm)	Ion Formula	m/z
$C_{15}H_{22}O_2$	97.01	234.1628	234.162	235.1693	-3.6	$C_{15}H_{23}O_2$	235.1701

Figure S11. The HRESIMS spectrum of compound **2**

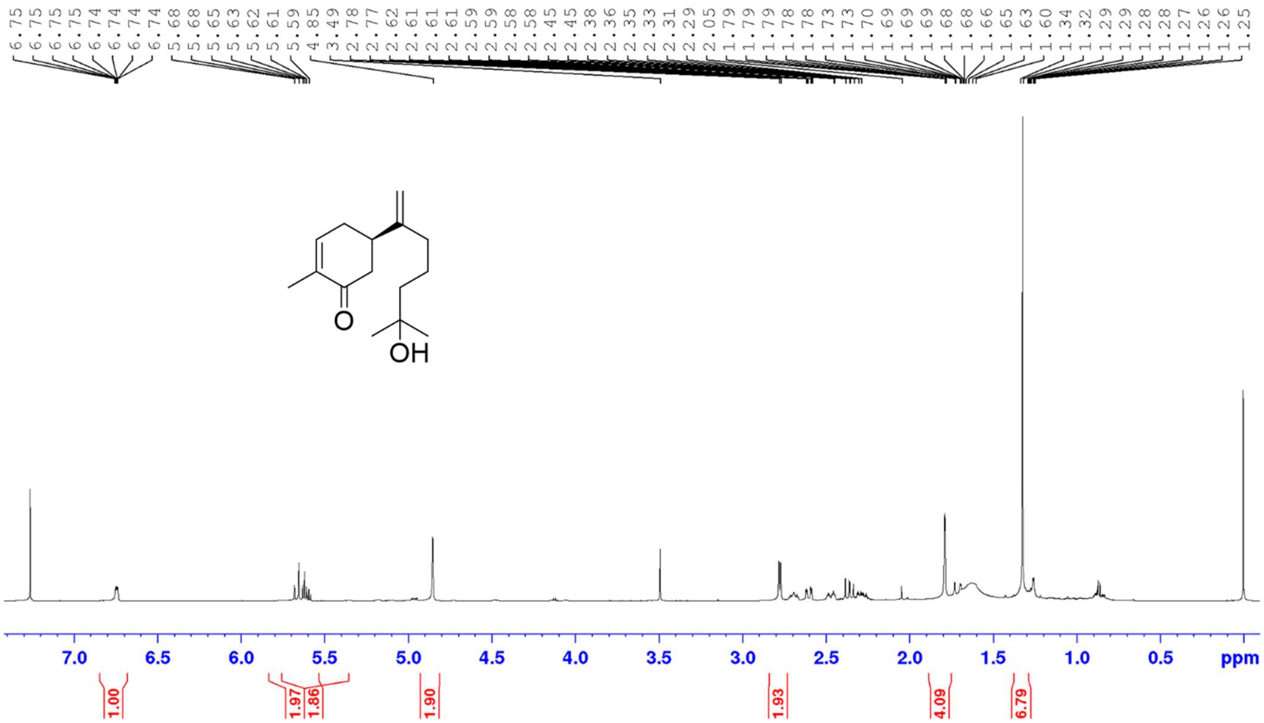

Figure S12. The ^{1}HNMR spectrum of compound **2**

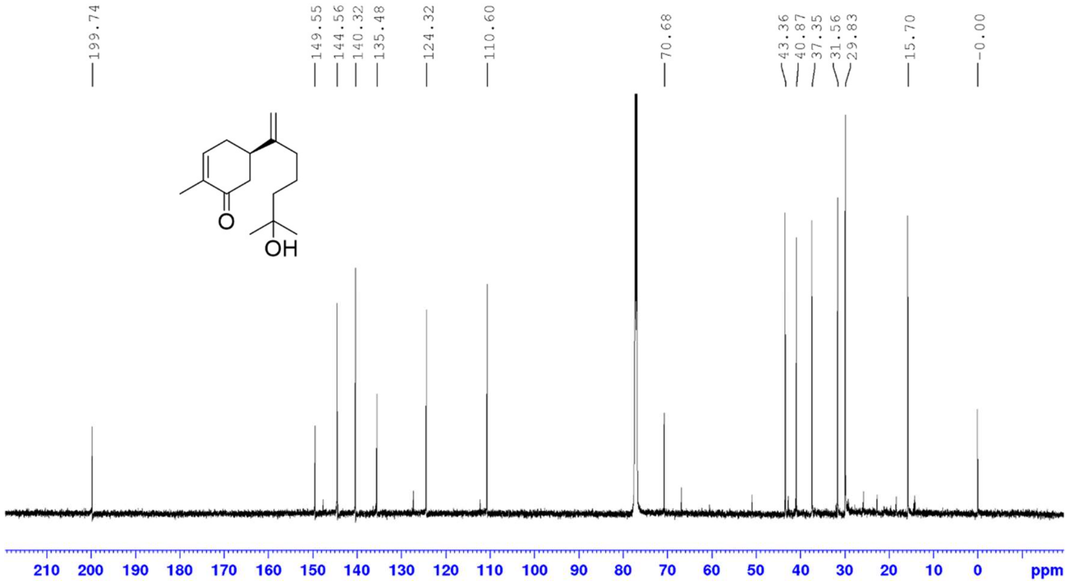

Figure S13. The ^{13}CNMR spectrum of compound **2**

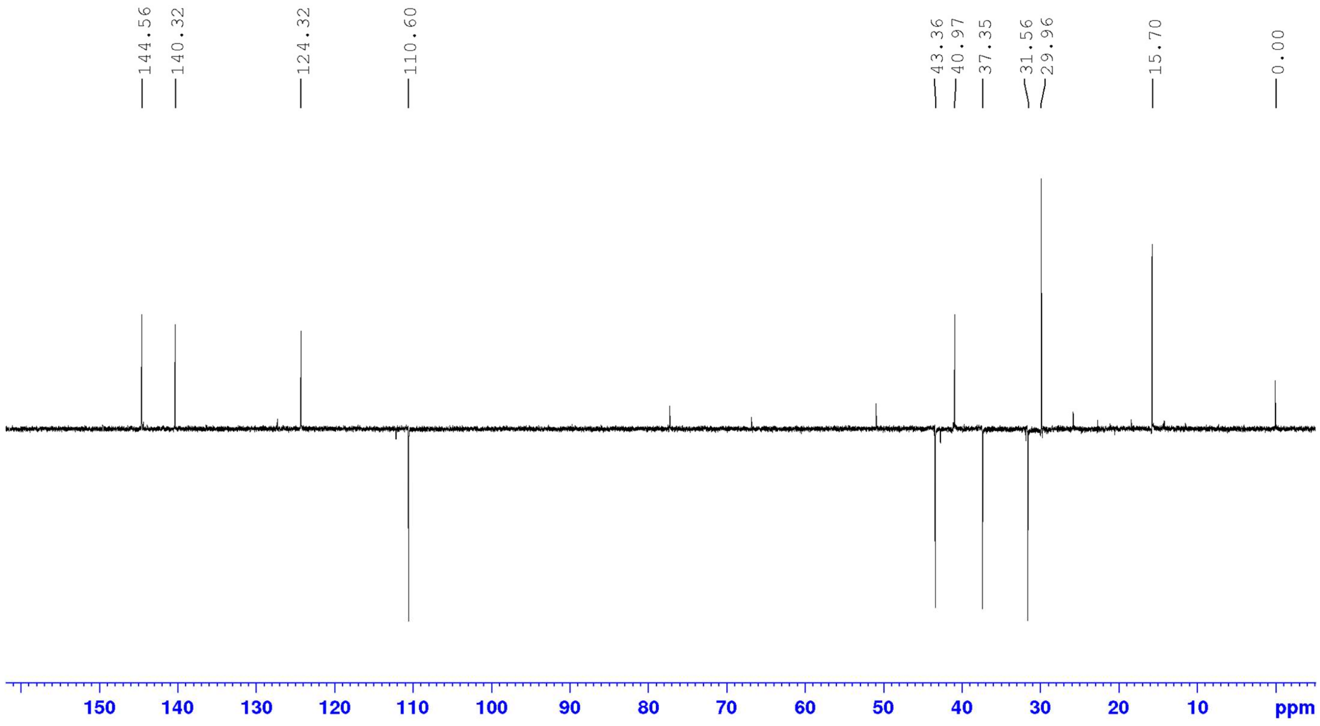

Figure S14. The DEPT135 spectrum of compound **2**

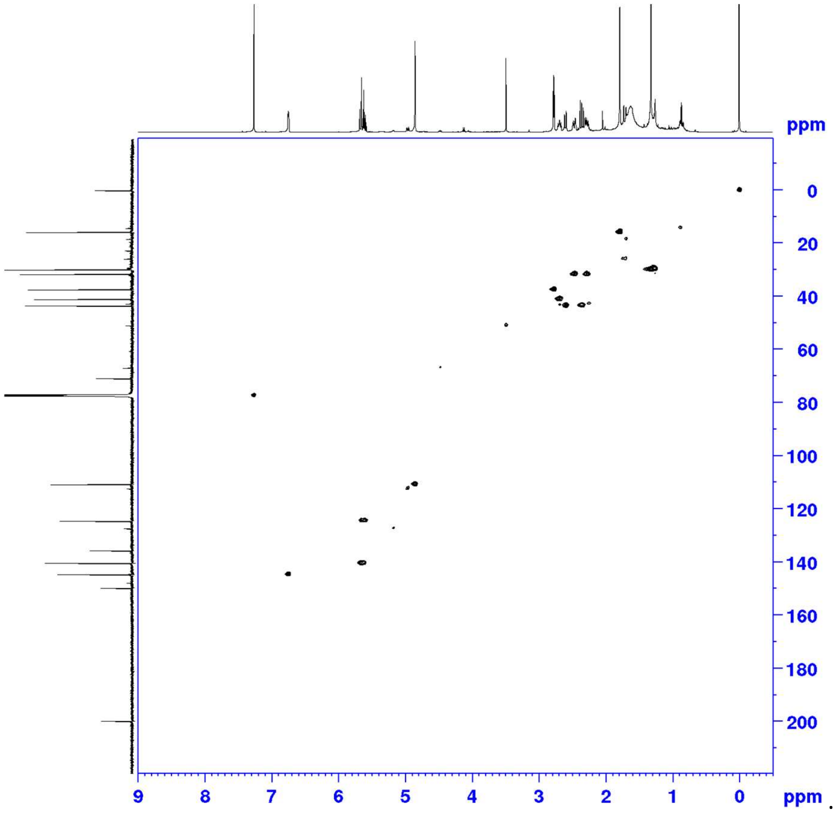

Figure S15. The HSQC spectrum of compound **2**

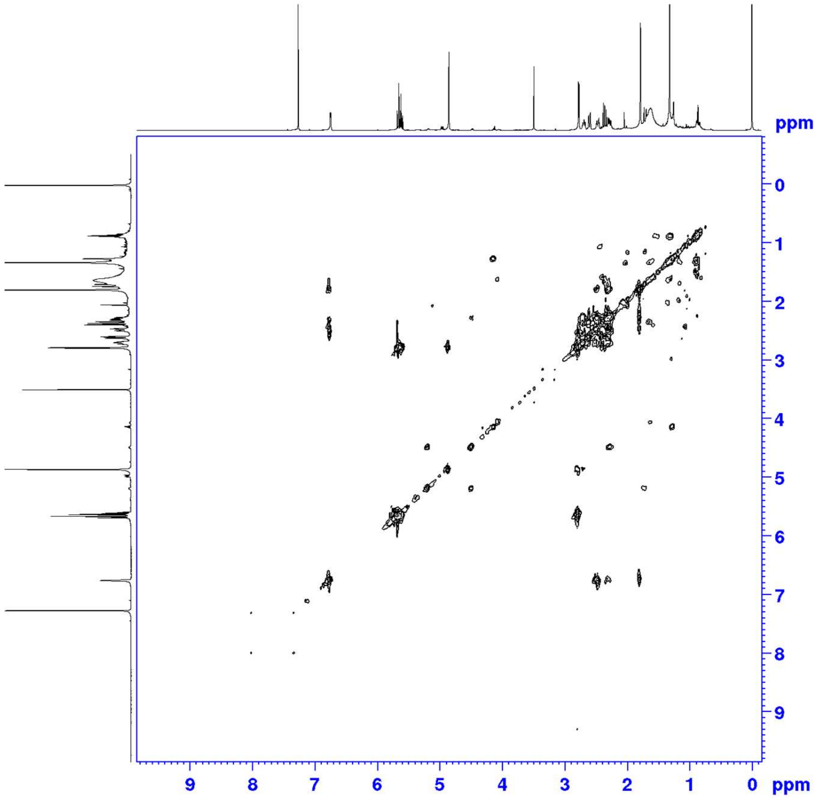

Figure S16. The ^{1}H-^{1}H COSY spectrum of compound **2**

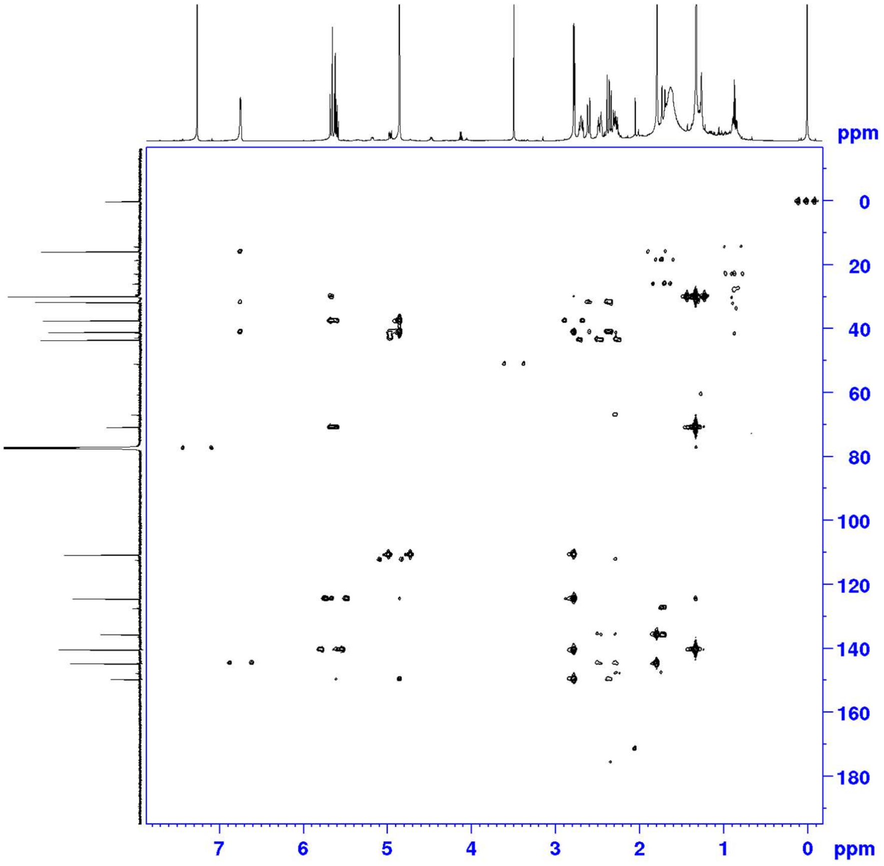

Figure S17. The HMBC spectrum of compound **2**

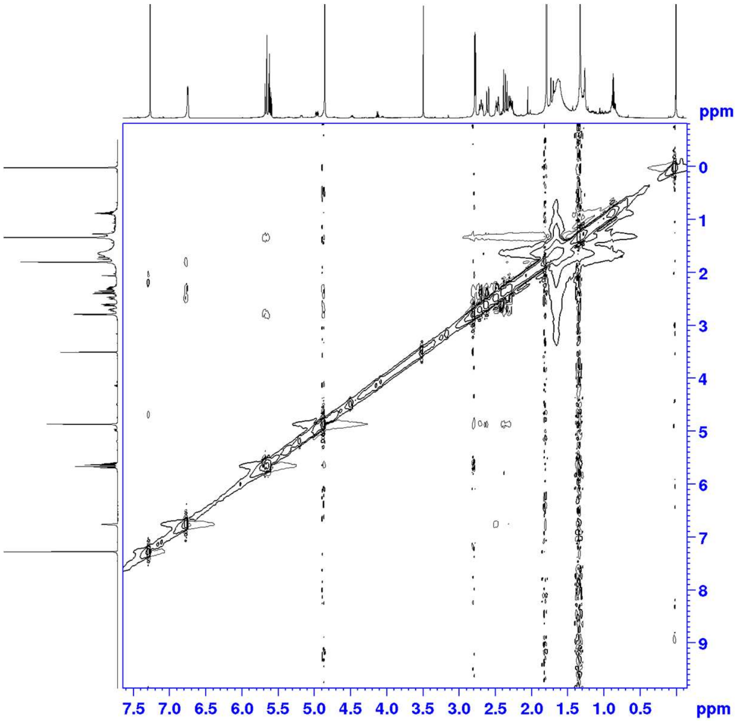

Figure S18. The NOESY spectrum of compound **2**

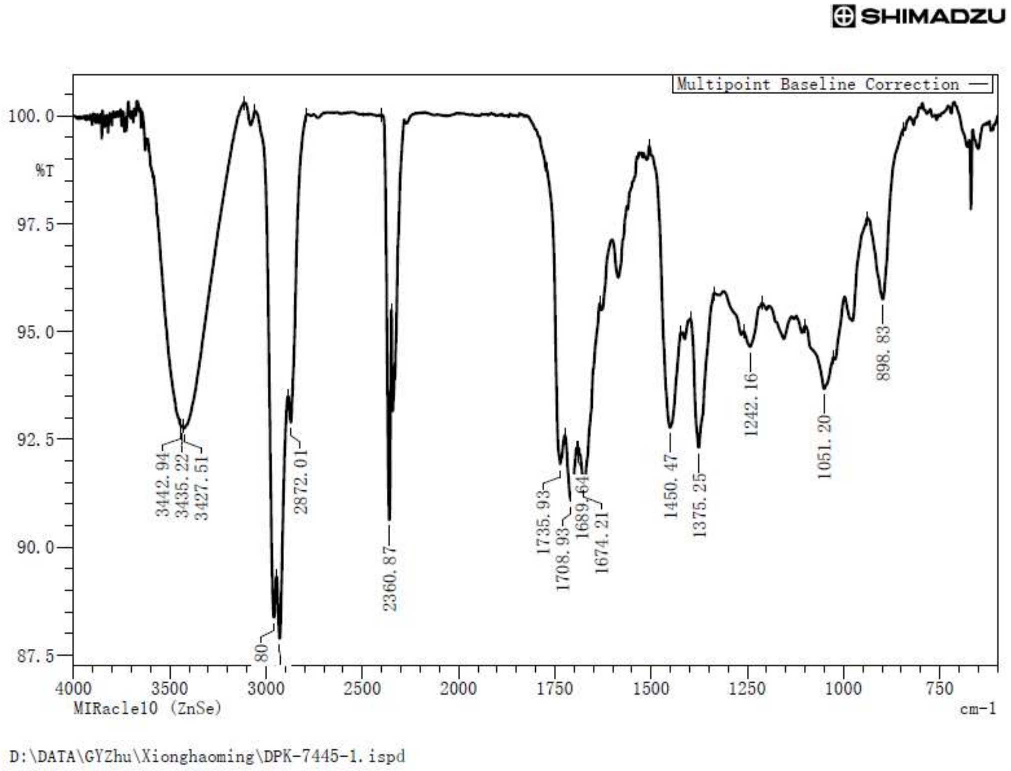

Figure S19. The IR spectrum of compound **2**

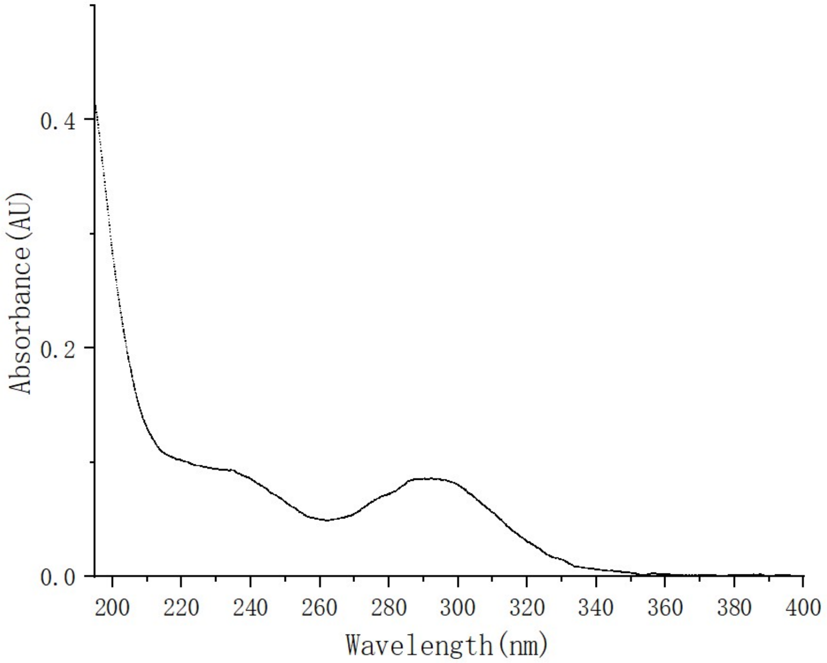

Figure S20. The UV spectrum of compound **2**

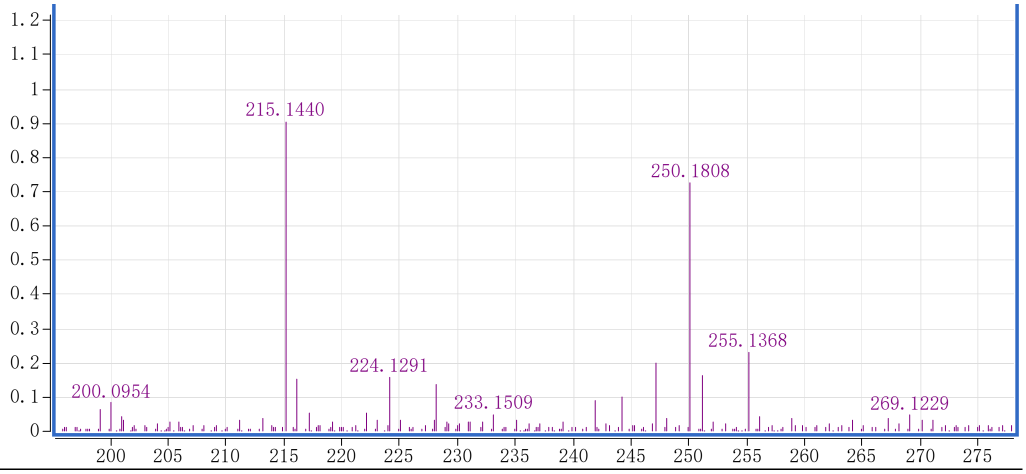

Formula	Score	Mass	Mass (MFG)	m/z (Calc)	Diff (ppm)	Ion Formula	m/z
$C_{15}H_{20}O_2$	98.22	232.147	232.1463	250.1802	-2.78	$C_{15}H_{24}NO_2$	250.1808

Figure S21. The HRESIMS spectrum of compound **3**

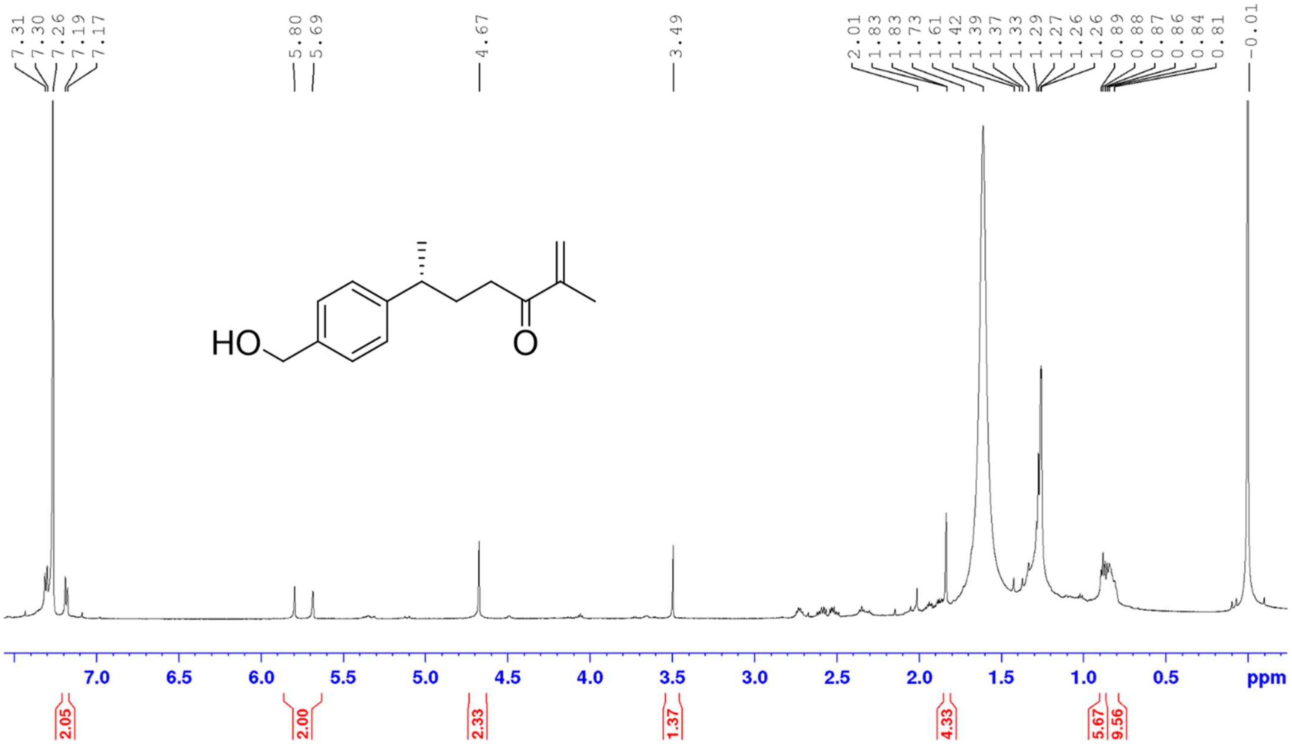

Figure S22. The ^{1}HNMR spectrum of compound **3**

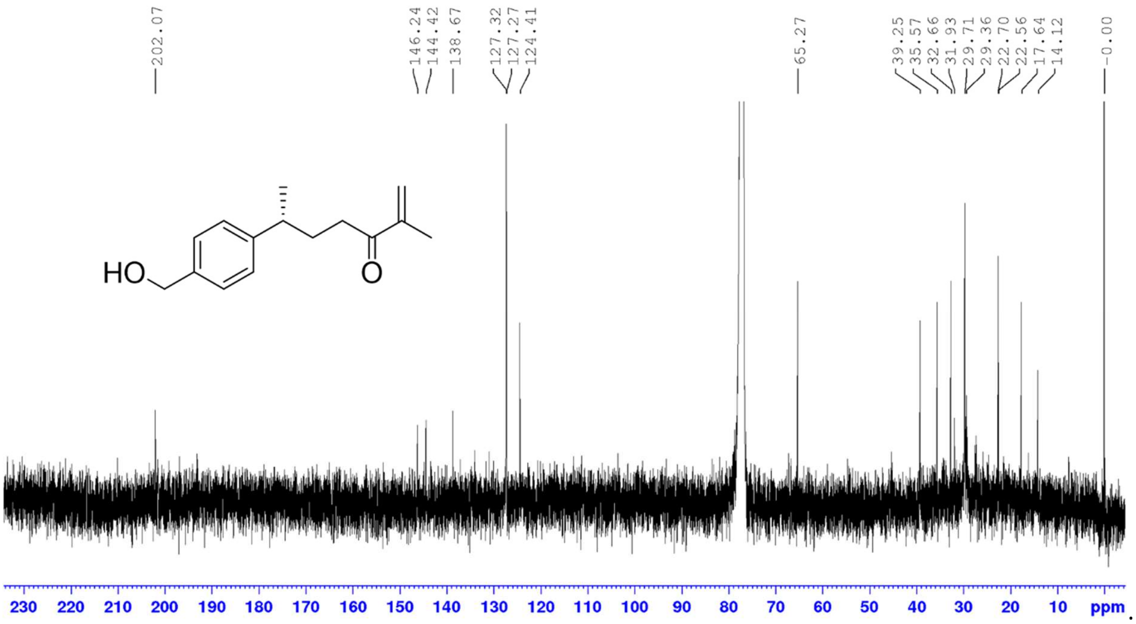

Figure S23. The ^{13}CNMR spectrum of compound **3**

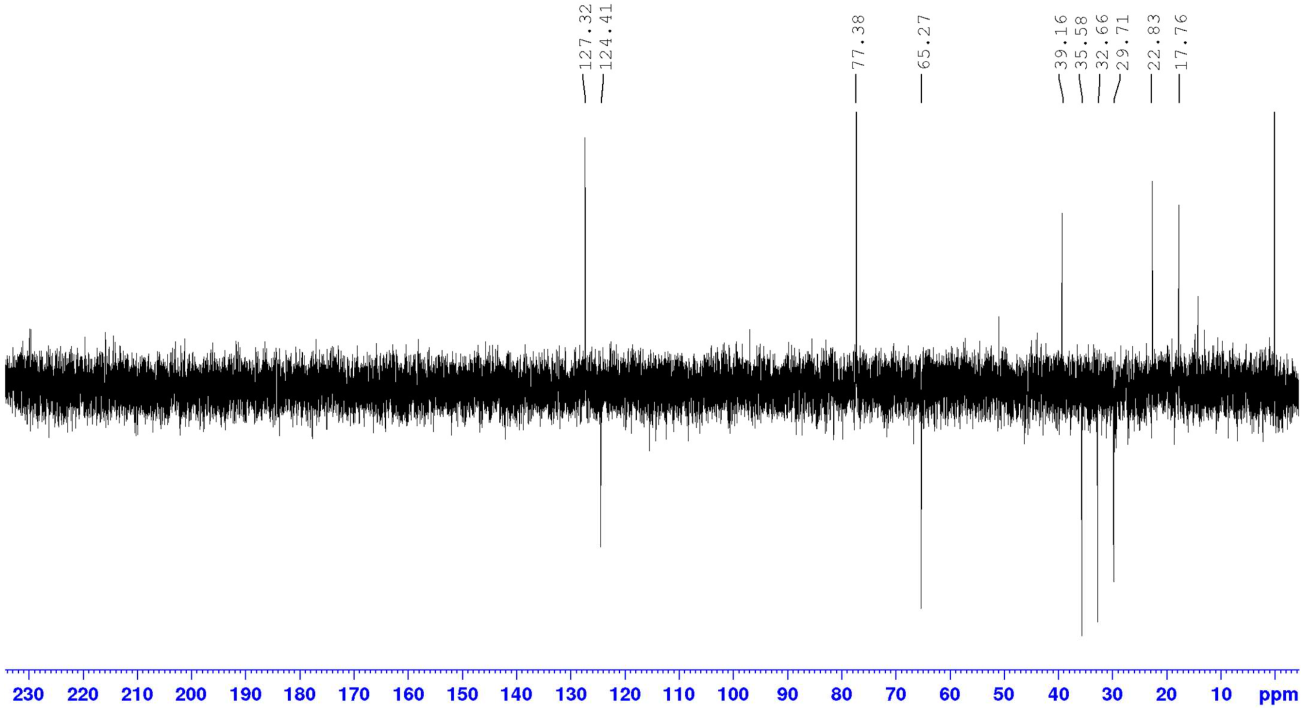

Figure S24. The DEPT135 spectrum of compound **3**

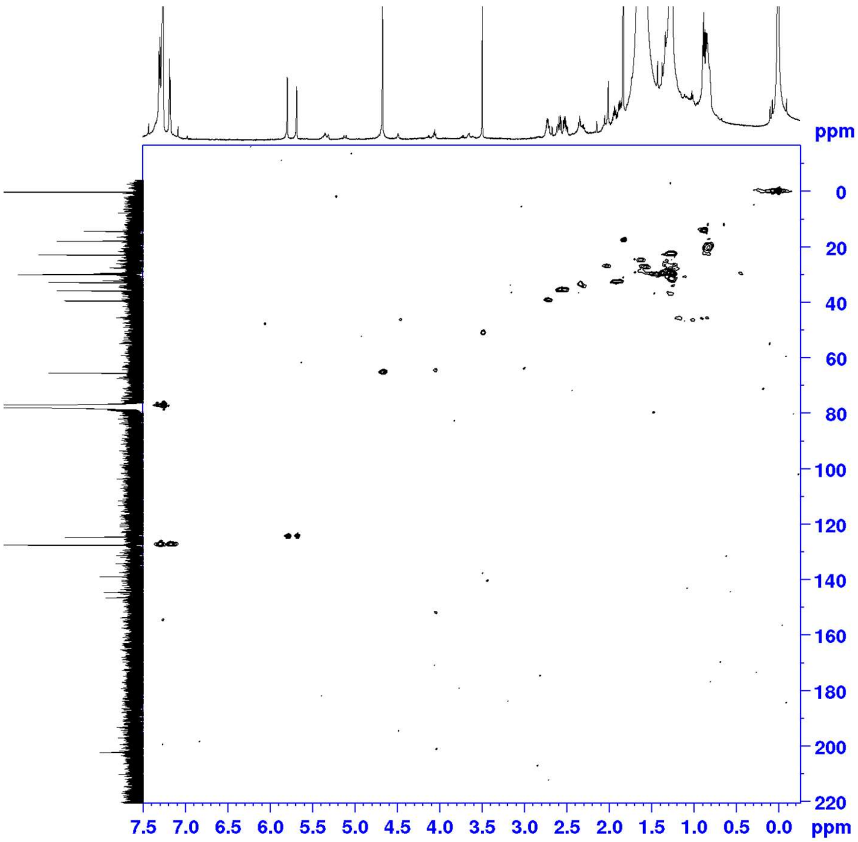

Figure S25. The HSQC spectrum of compound **3**

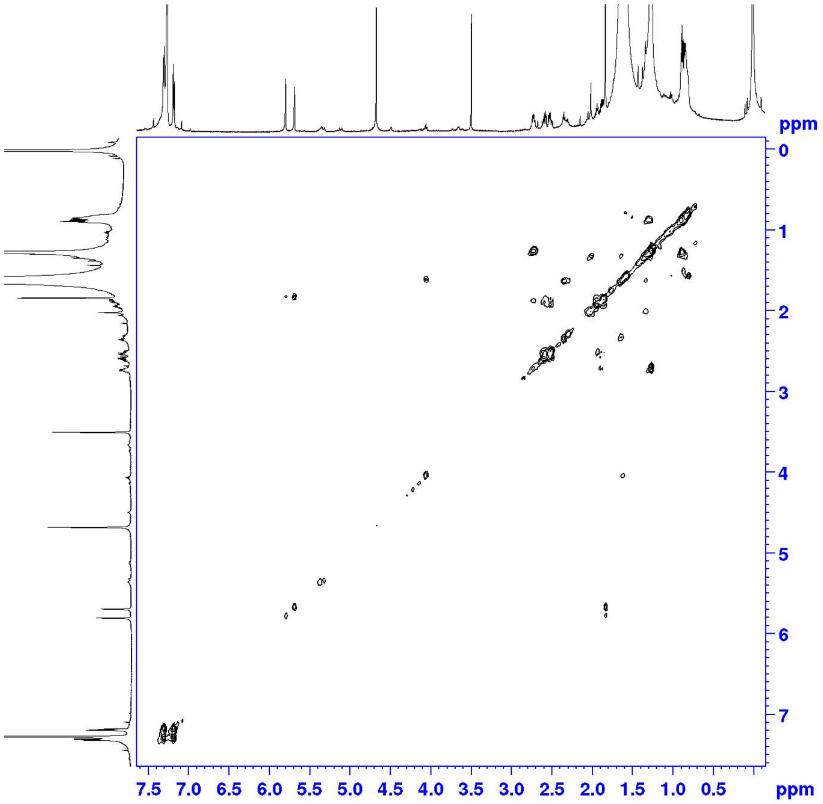

Figure S26. The ^{1}H-^{1}H COSY spectrum of compound **3**

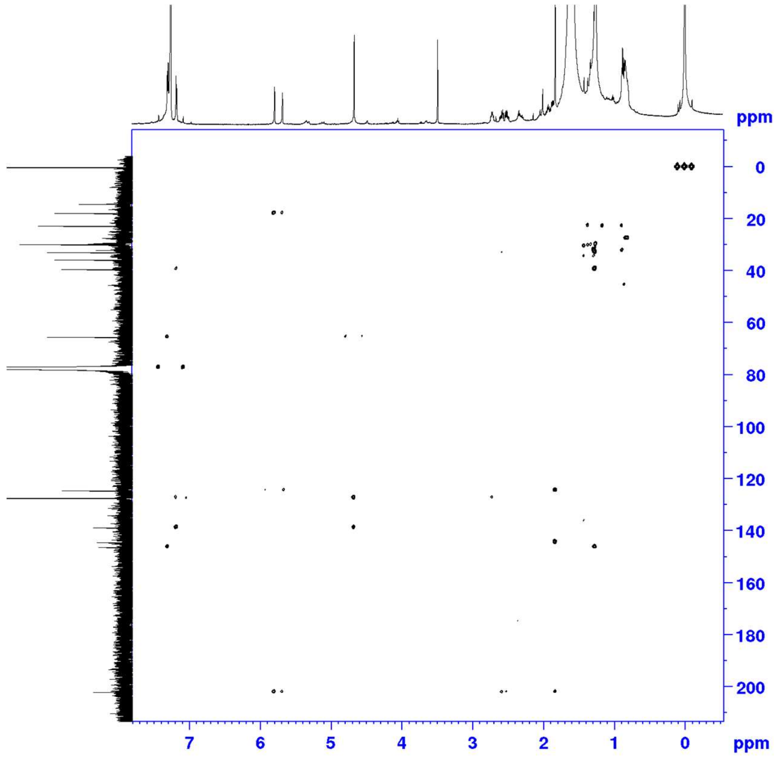

Figure S27. The HMBC spectrum of compound **3**

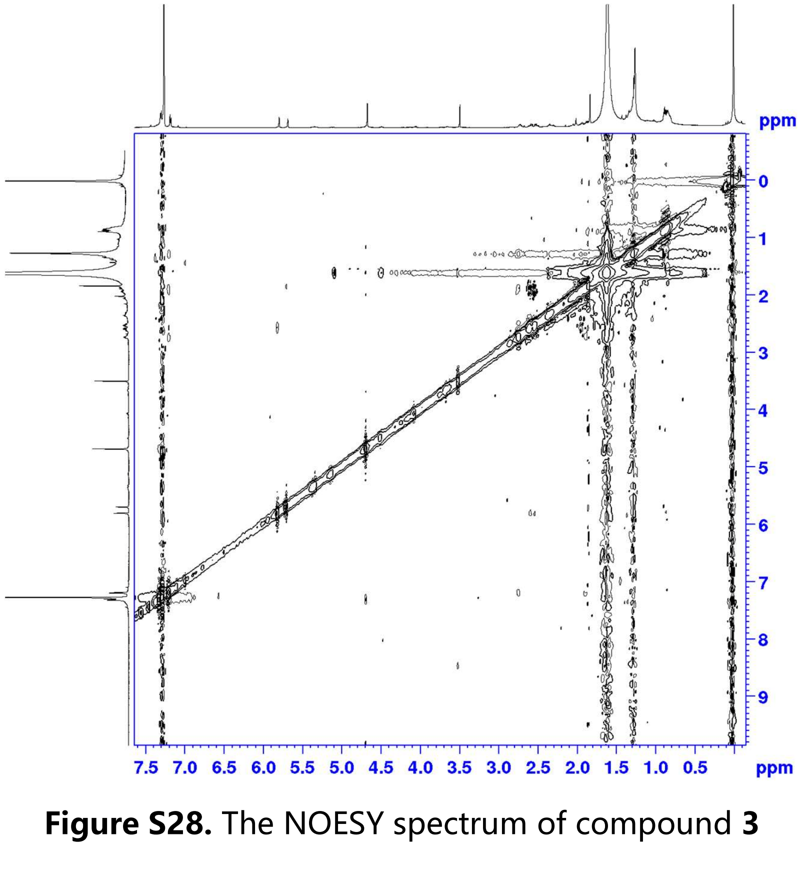

Figure S28. The NOESY spectrum of compound **3**

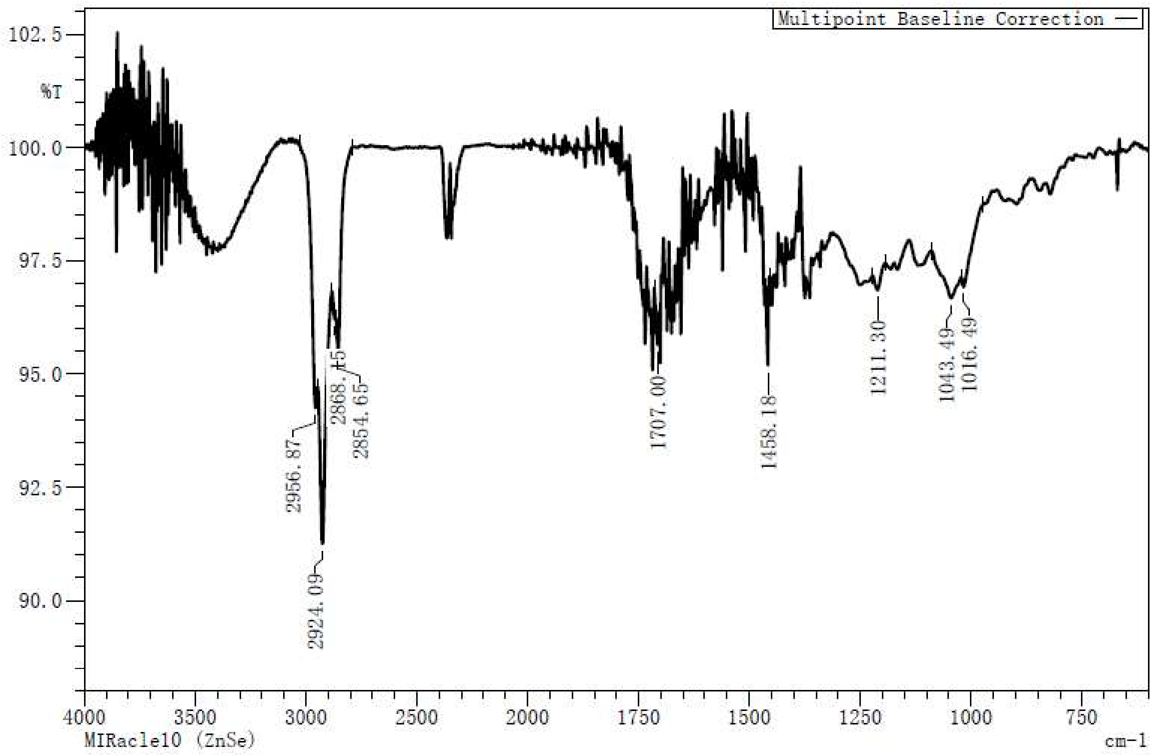

Figure S29. The IR spectrum of compound **3**

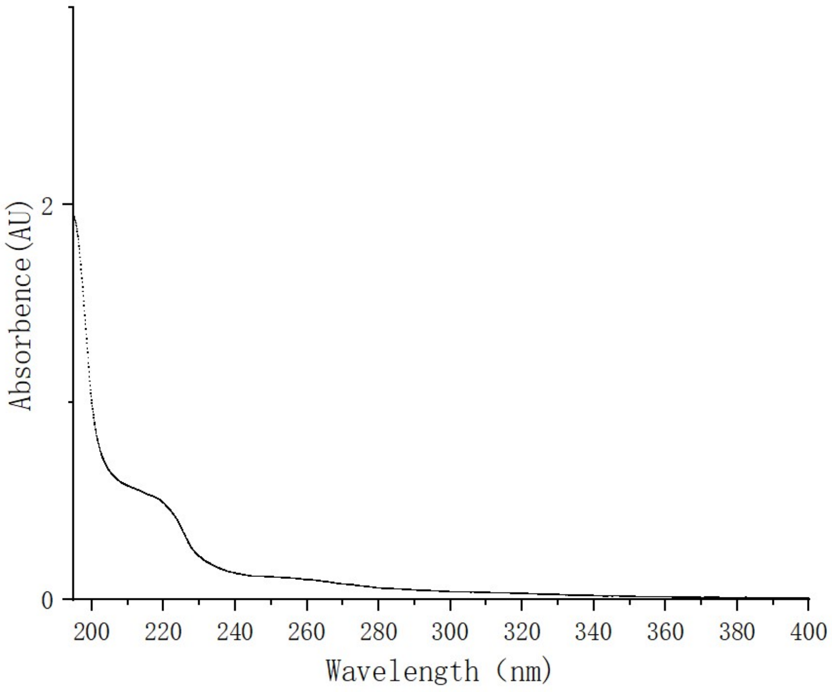

Figure S30. The UV spectrum of compound **3**

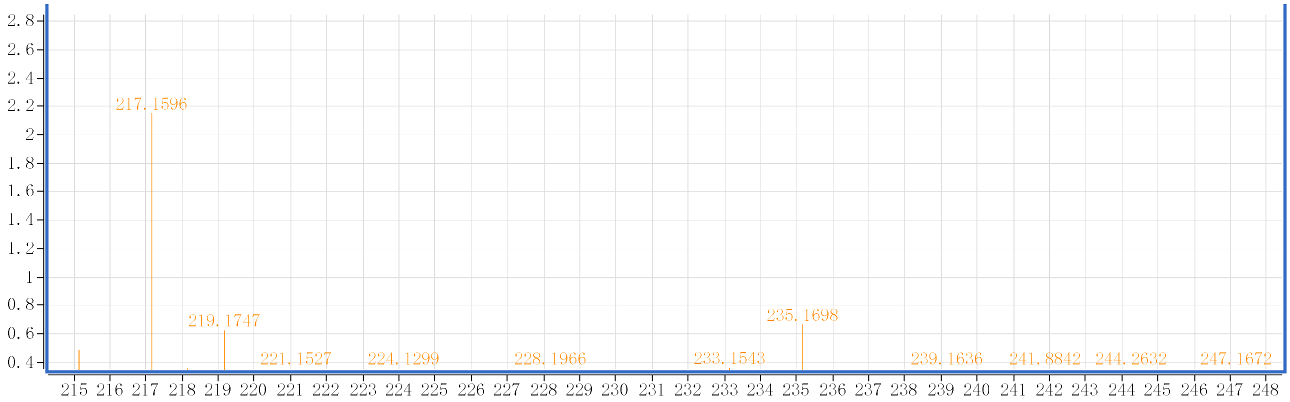

Formula	Score	Mass	Mass (MFG)	m/z (Calc)	Diff (ppm)	Ion Formula	m/z
$C_{15}H_{22}O_2$	99.45	234.1616	234.162	235.1693	1.52	$C_{15}H_{23}O_2$	235.1689

Figure S31. The HRESIMS spectrum of compound **4**

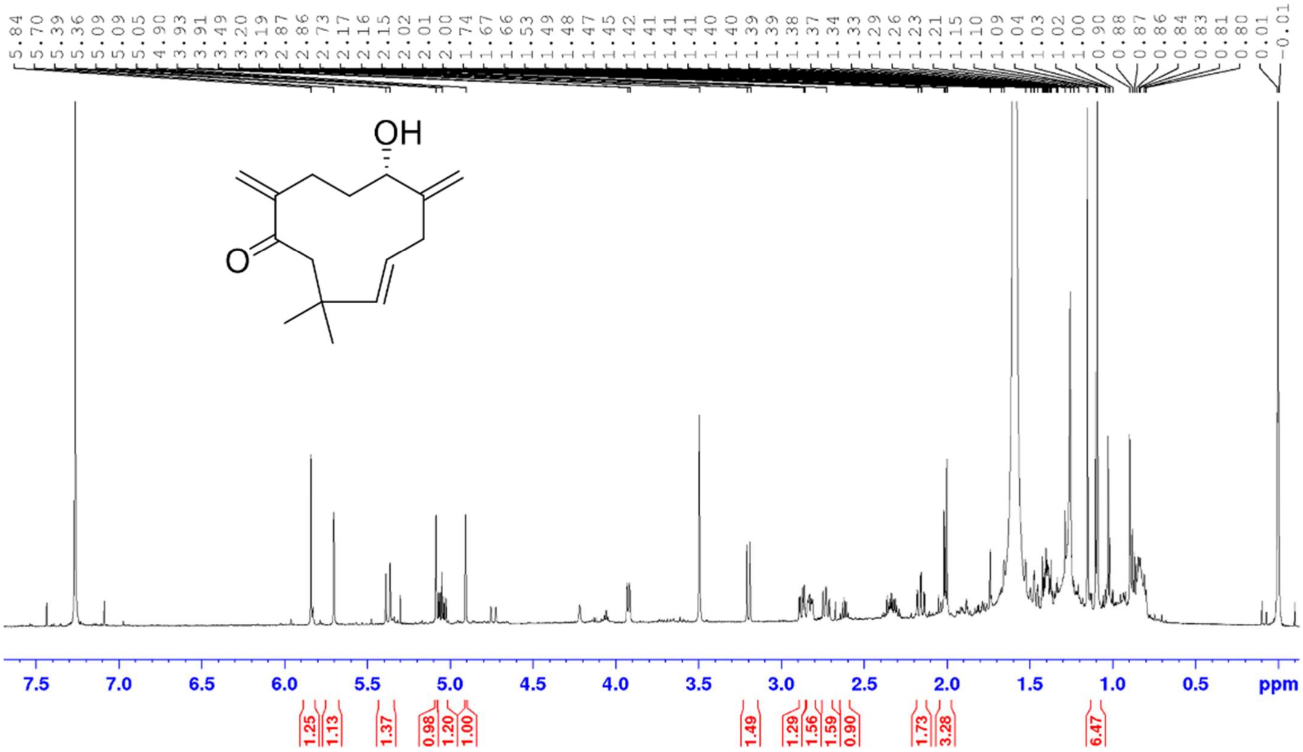

Figure S32. The ^{1}HNMR spectrum of compound **4**

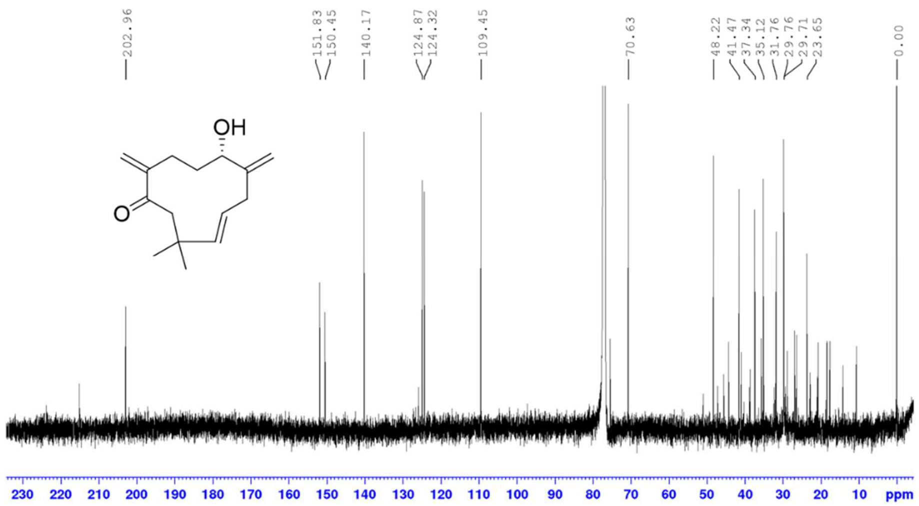

Figure S33. The ^{13}CNMR spectrum of compound **4**

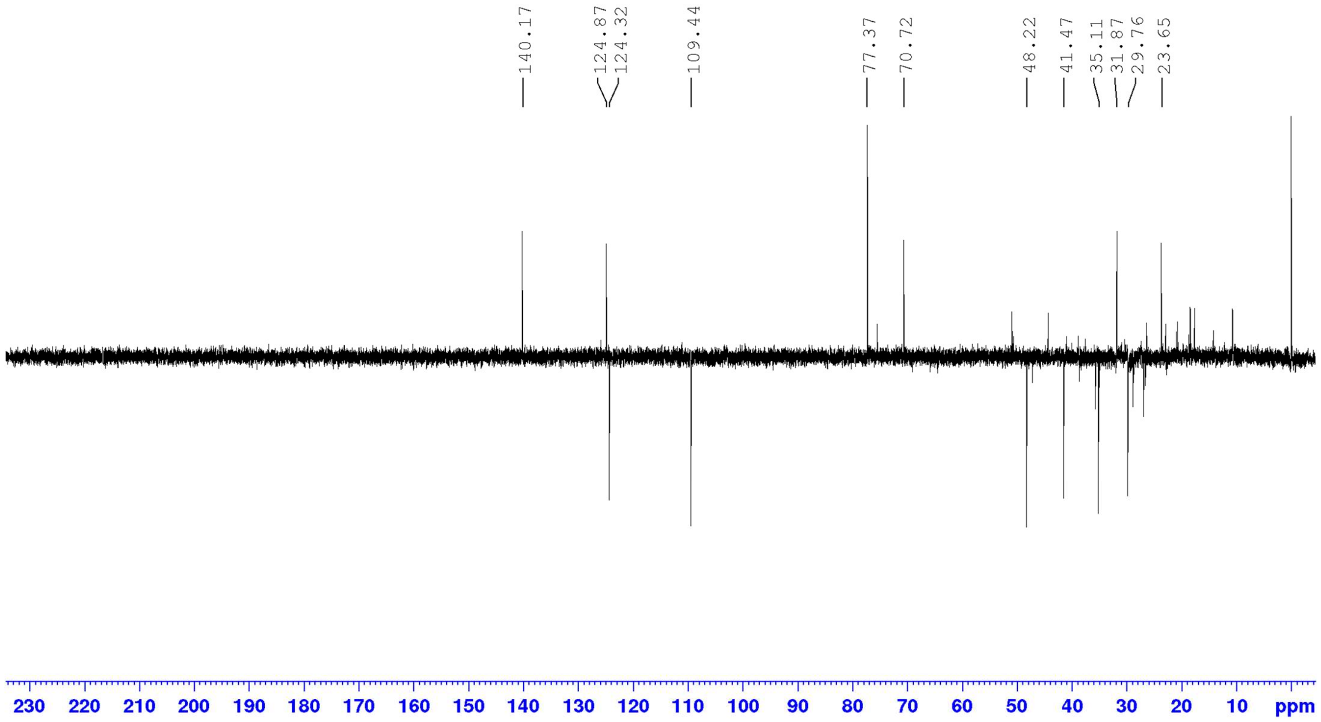

Figure S34. The DEPT135 spectrum of compound **4**

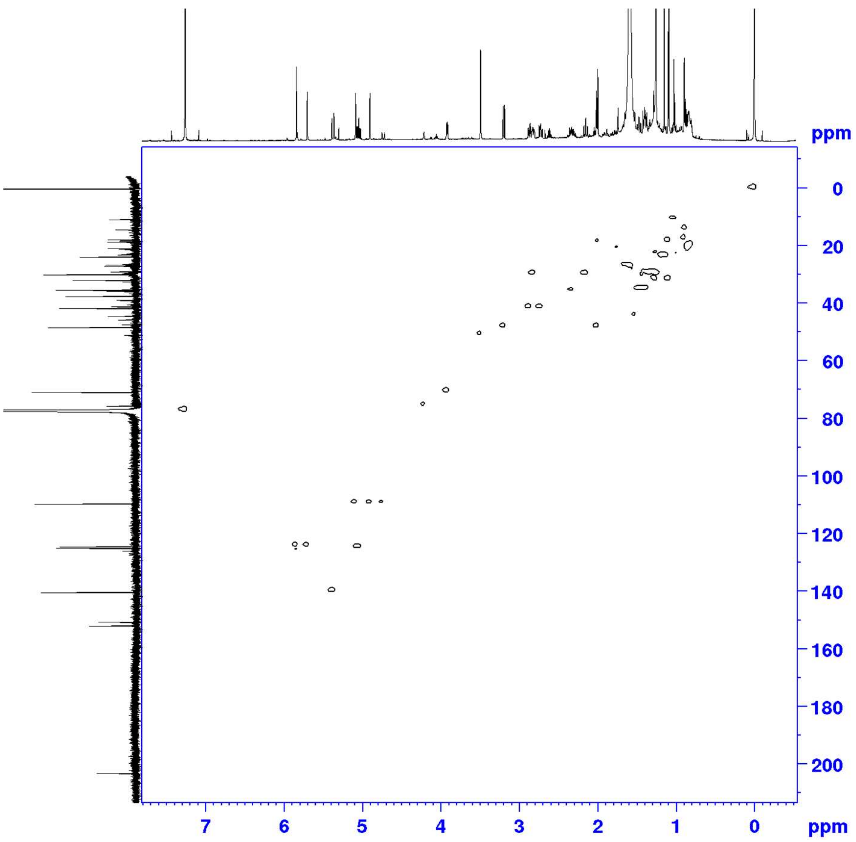

Figure S35. The HSQC spectrum of compound **4**

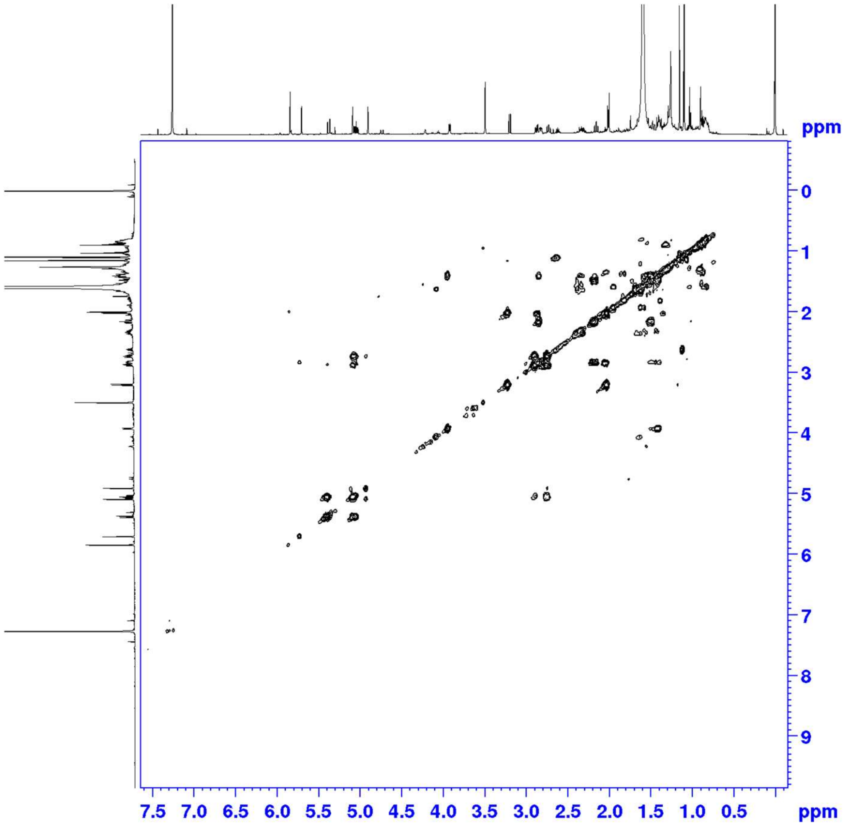

Figure S36. The ^{1}H-^{1}H COSY spectrum of compound **4**

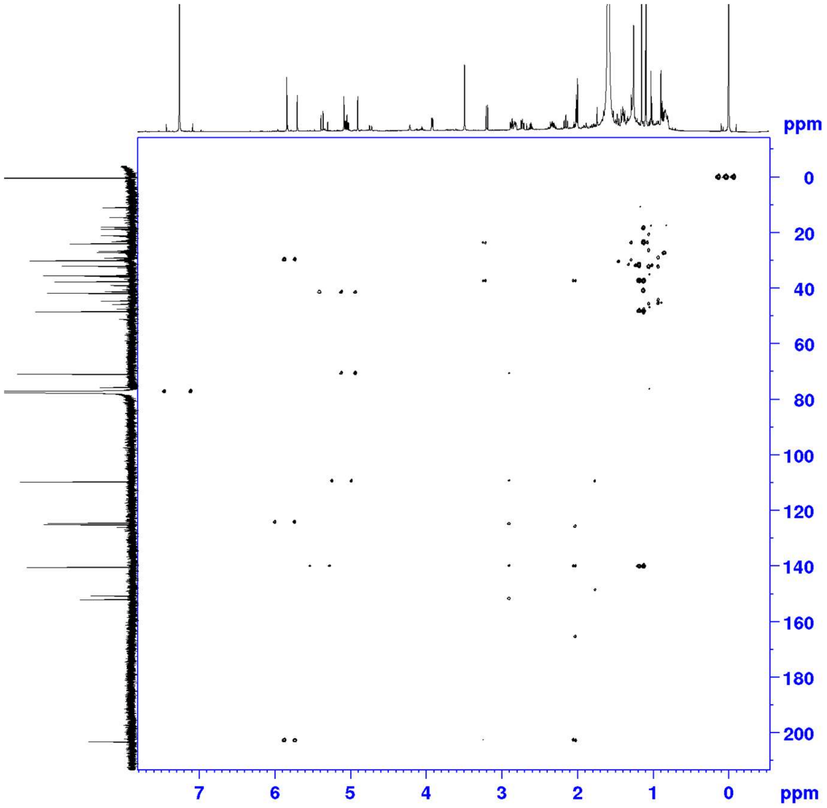

Figure S37. The HMBC spectrum of compound **4**

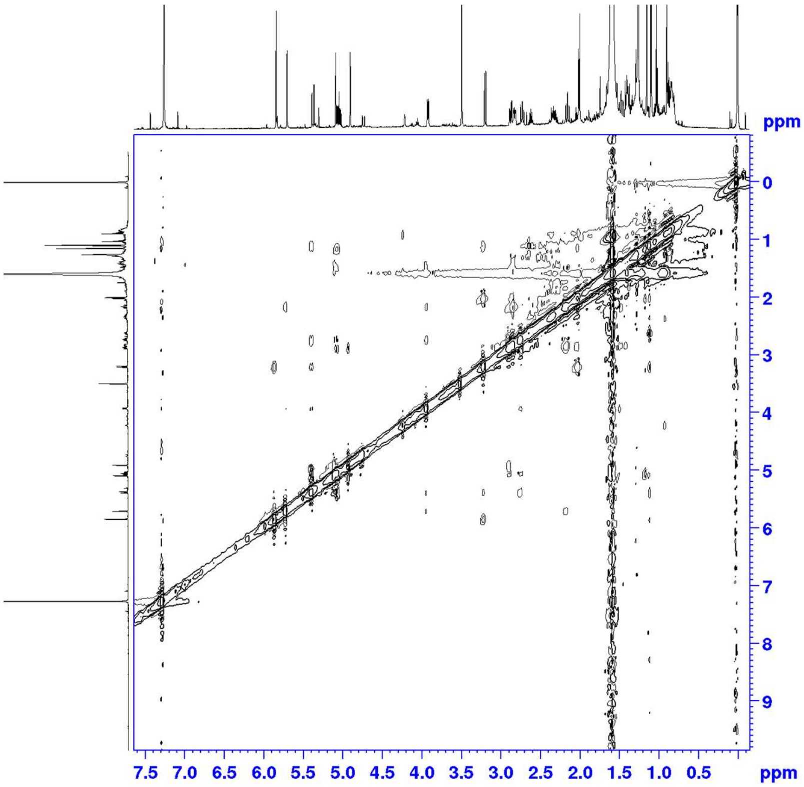

Figure S38. The NOESY spectrum of compound **4**

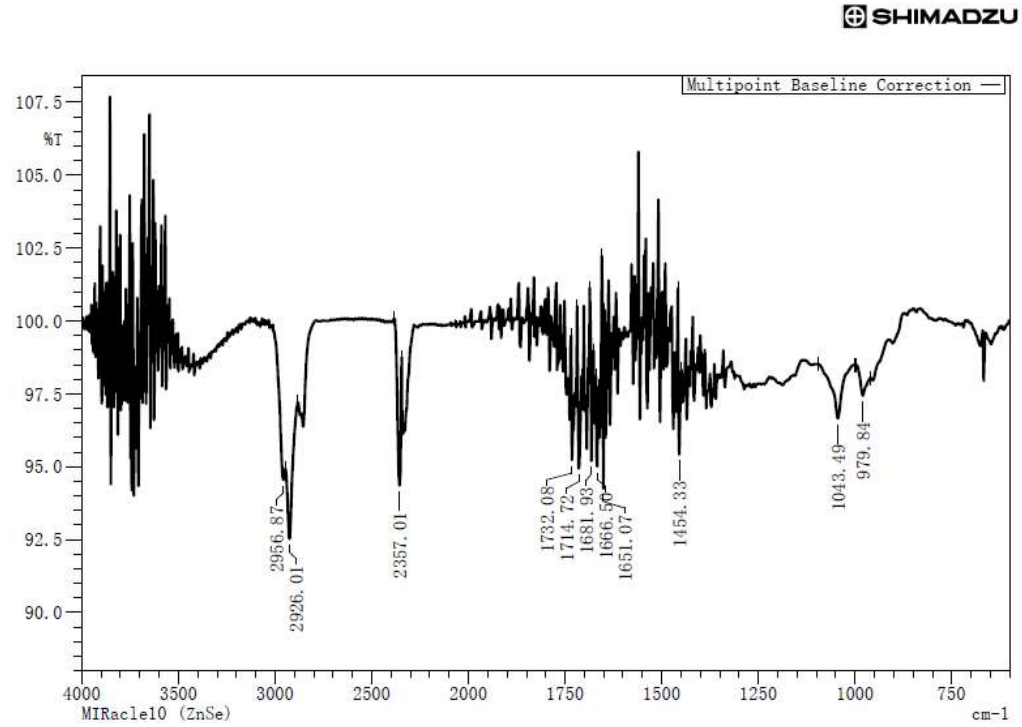

Figure S39. The IR spectrum of compound **4**

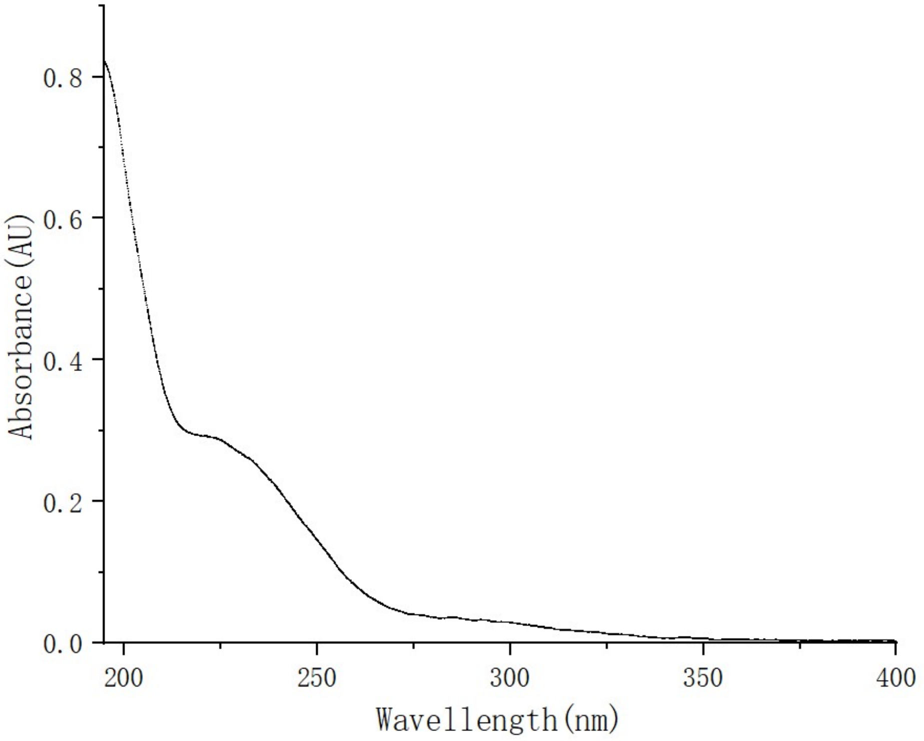

Figure S40. The UV spectrum of compound **4**

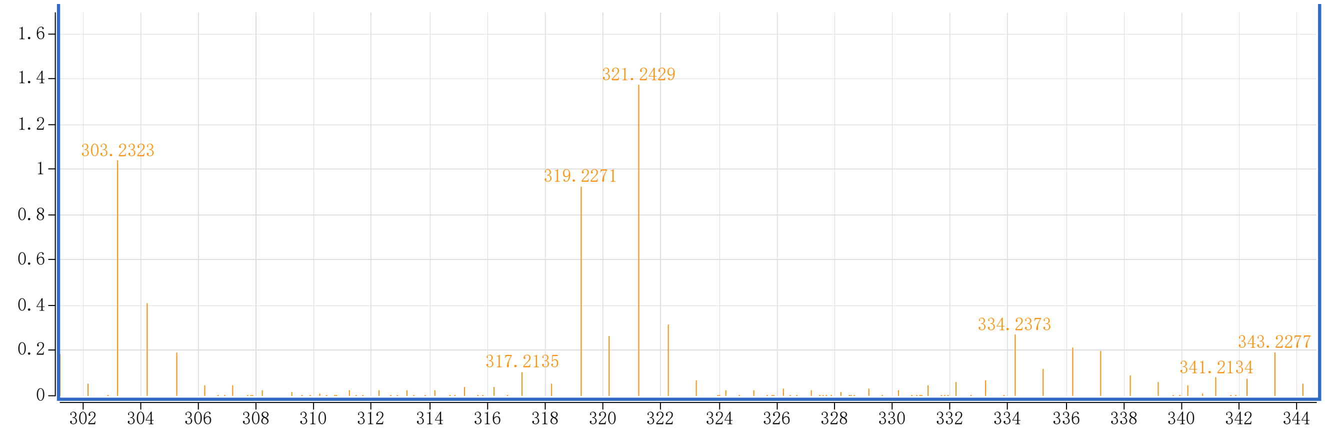

Formula	Score	Mass	Mass (MFG)	m/z (Calc)	Diff (ppm)	Ion Formula	m/z
$C_{20}H_{32}O_3$	99.25	320.2356	320.2351	321.2424	-1.49	$C_{20}H_{33}O_3$	321.2429

Figure S41. The HRESIMS spectrum of compound **5**

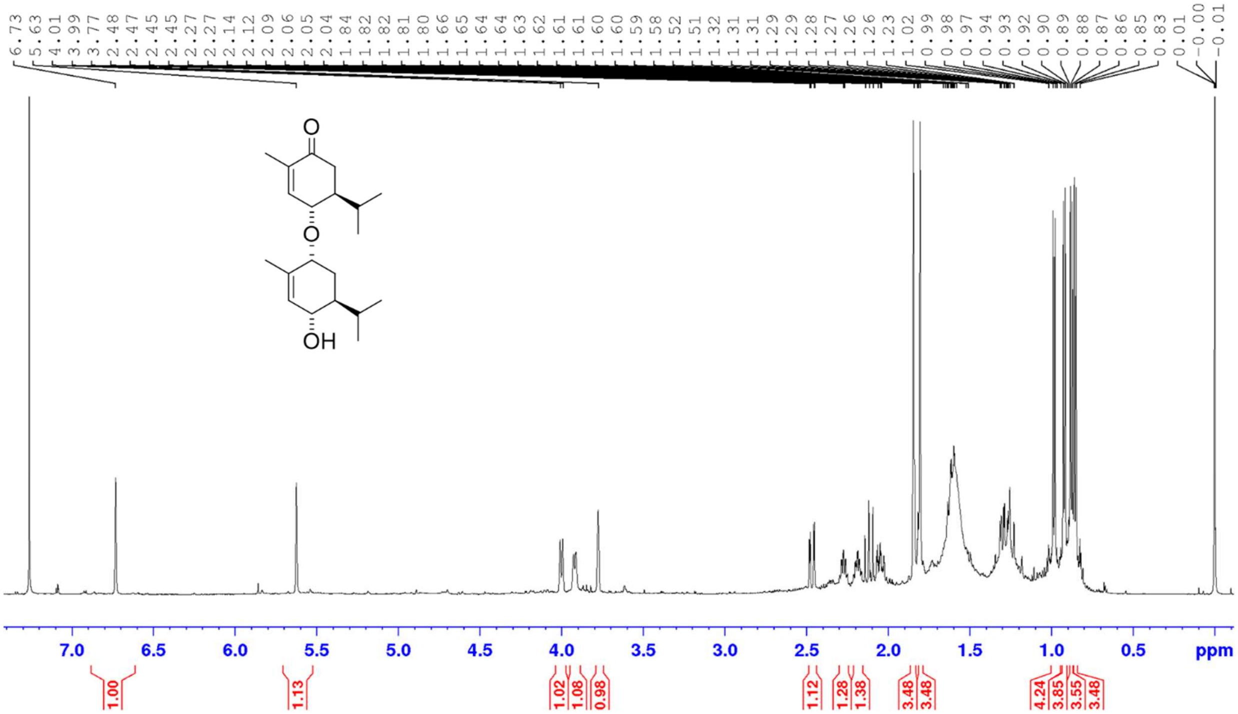

Figure S42. The ^{1}HNMR spectrum of compound **5**

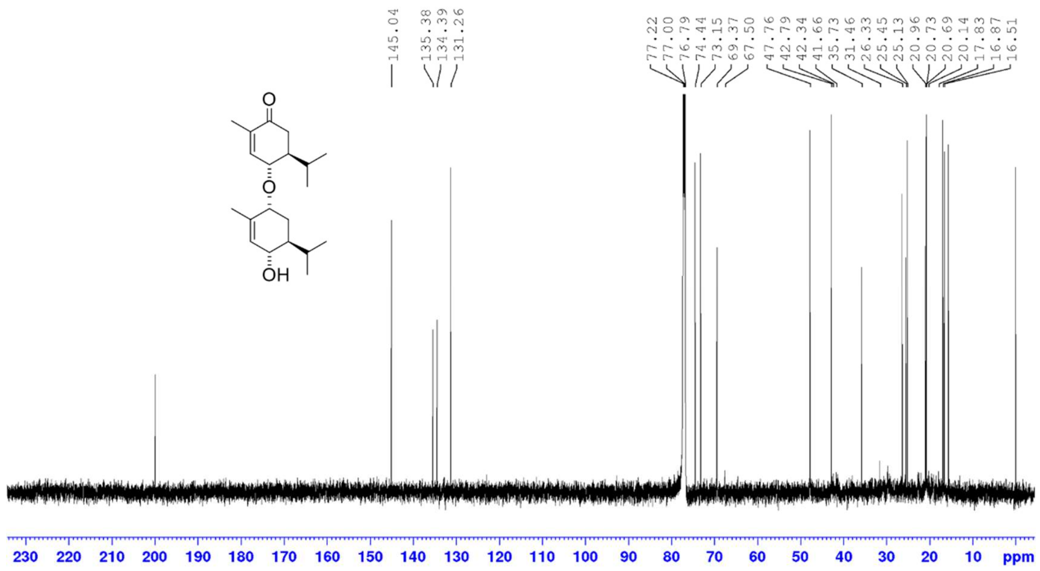

Figure S43. The ^{13}CNMR spectrum of compound **5**

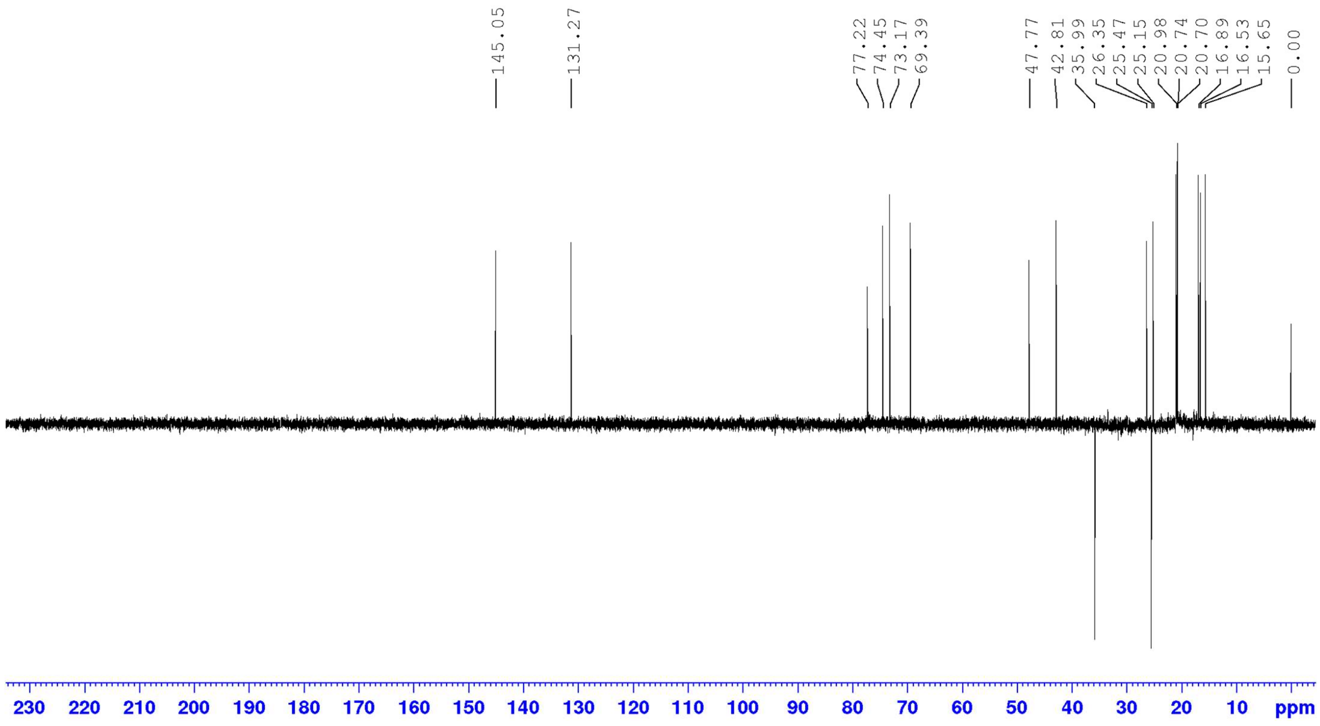

Figure S44. The DEPT135 spectrum of compound **5**

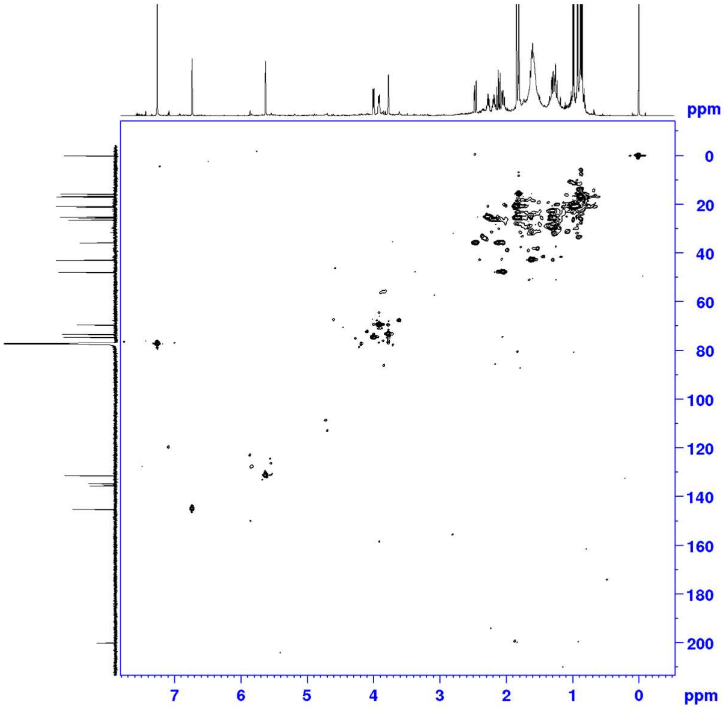

Figure S45. The HSQC spectrum of compound **5**

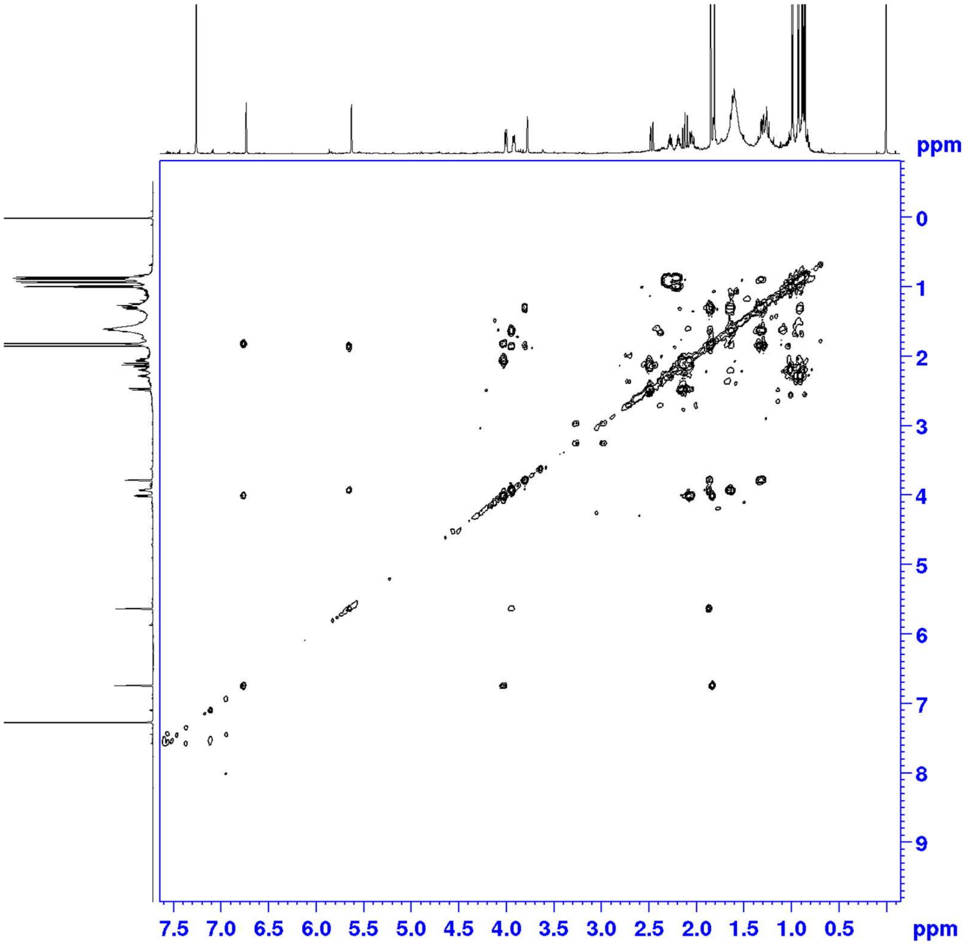

Figure S46. The ${}^{1}H$-${}^{1}H$ COSY spectrum of compound **5**

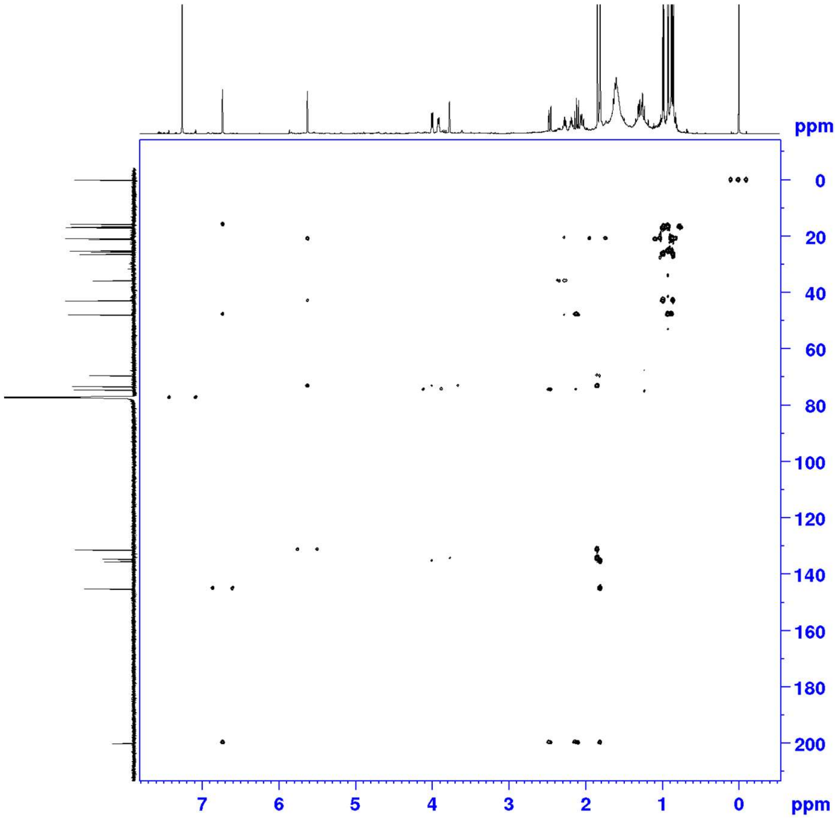

Figure S47. The HMBC spectrum of compound **5**

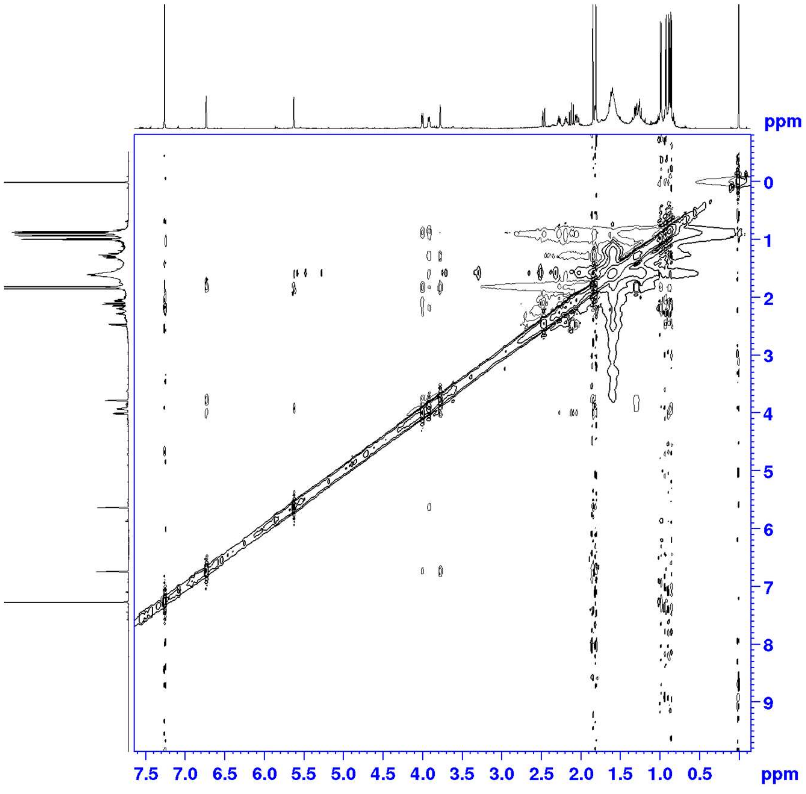

Figure S48. The NOESY spectrum of compound **5**

SHIMADZU

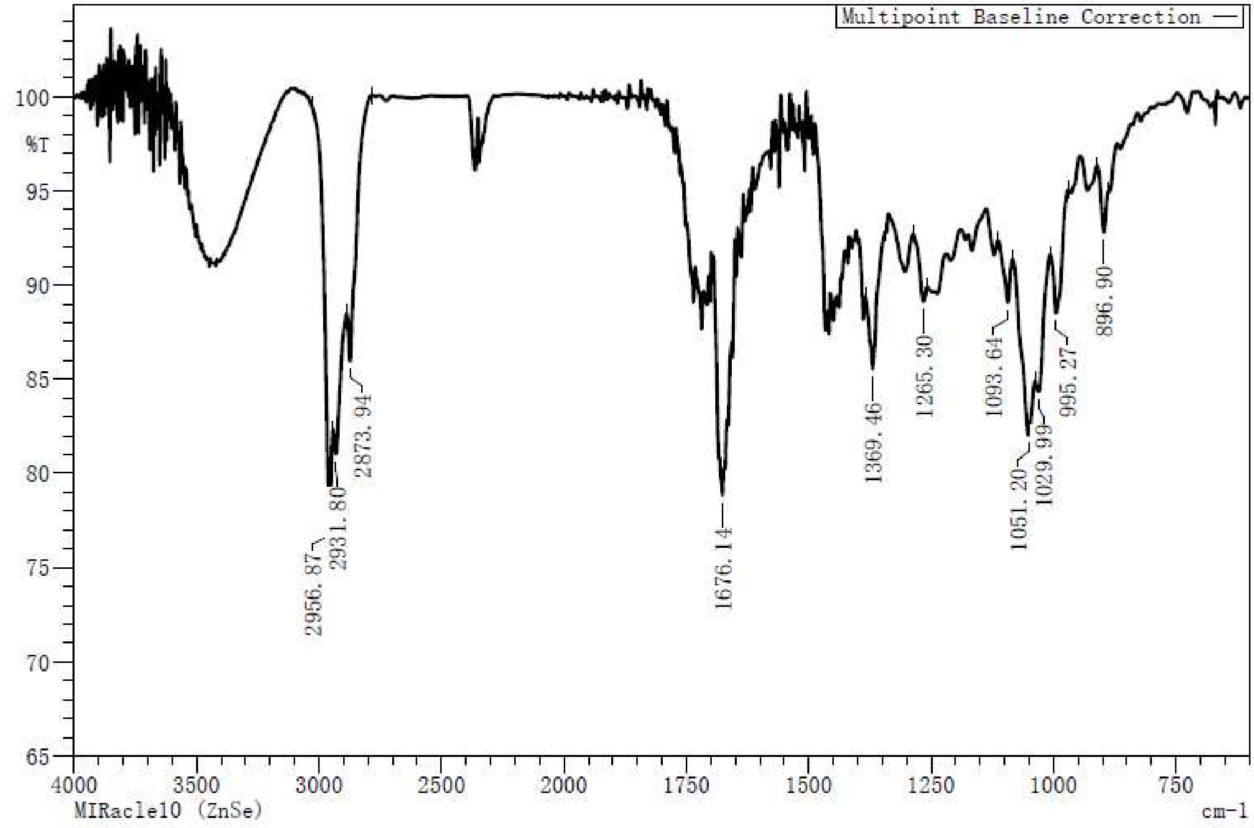

Figure S49. The IR spectrum of compound **5**

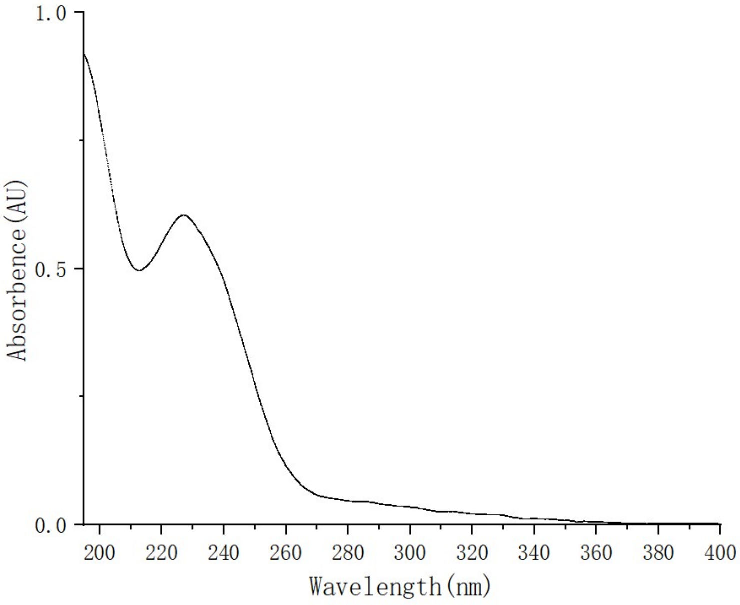

Figure S50. The IR spectrum of compound **5**

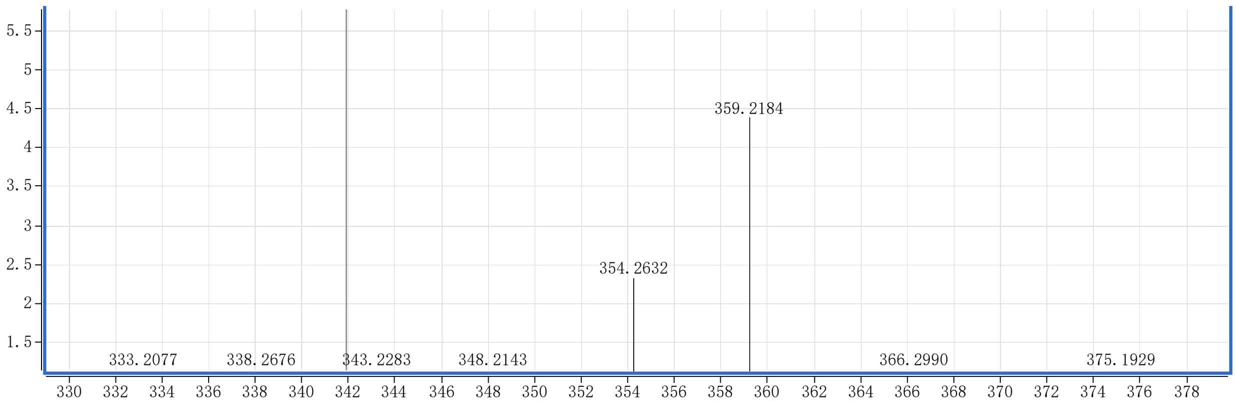

Formula	Score	Mass	Mass (MFG)	m/z (Calc)	Diff (ppm)	Ion Formula	m/z
$C_{20}H_{32}O_4$	97.61	336.2292	336.2301	359.2193	-1.49	$C_{20}H_{33}NaO_3$	359.2184

Figure S51. The HRESIMS spectrum of compound **6**

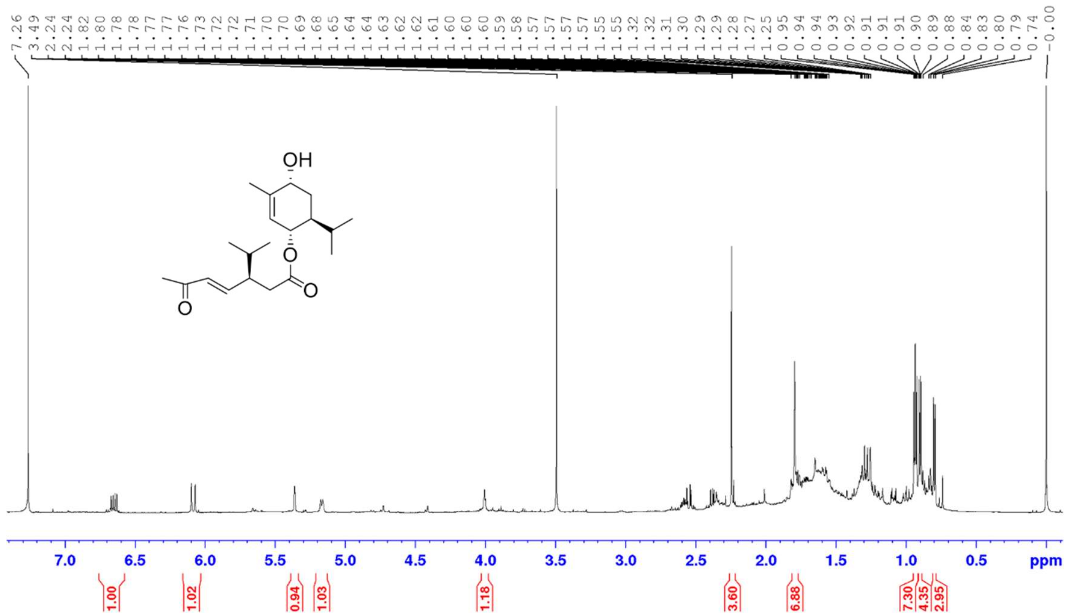

Figure S52. The ^{1}HNMR spectrum of compound **6**

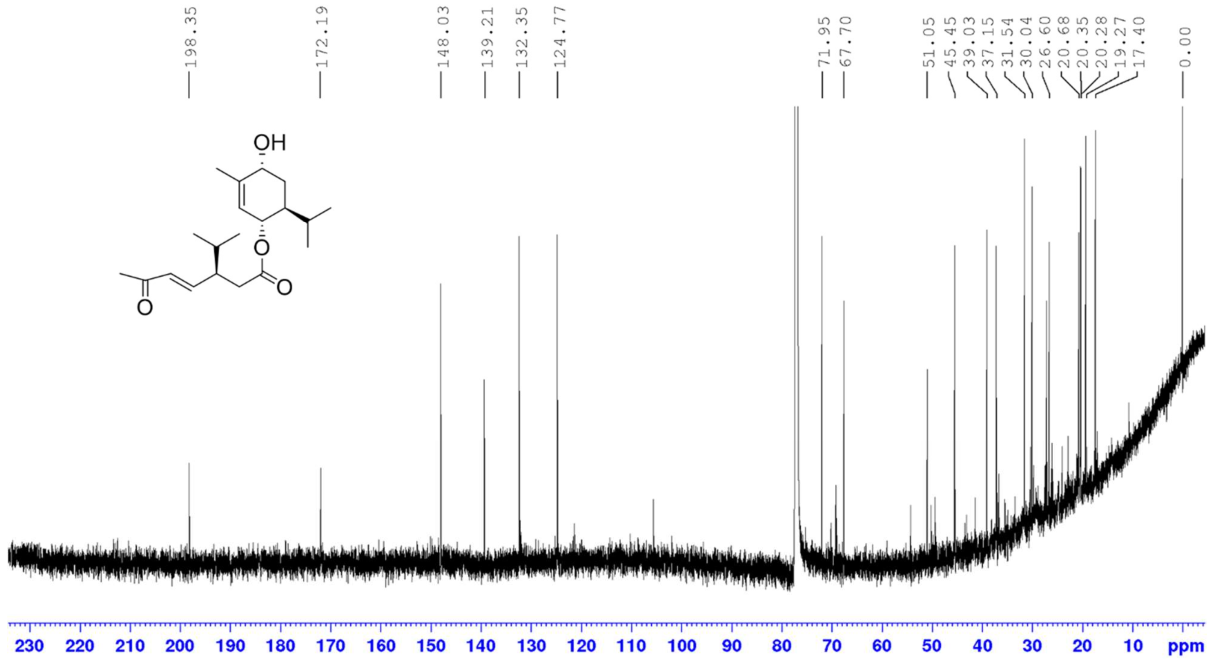

Figure S53. The ^{13}CNMR spectrum of compound **6**

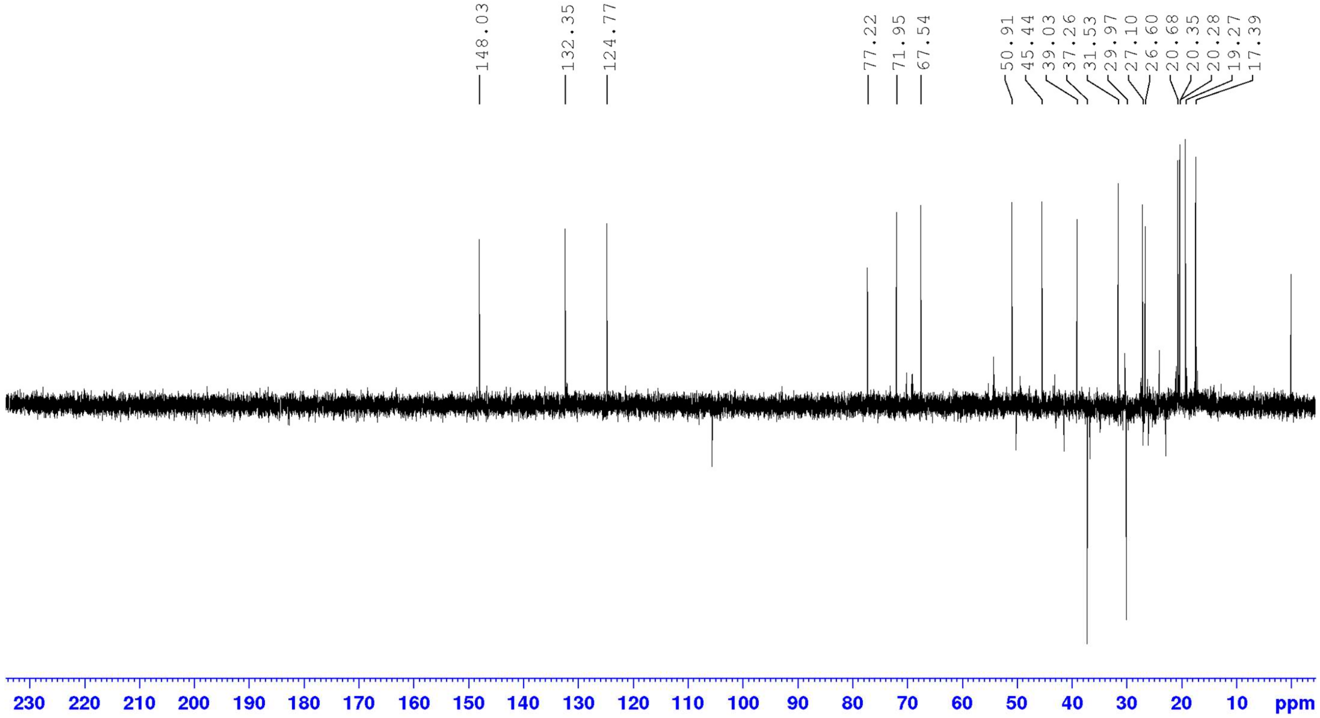

Figure S54. The DEPT135 spectrum of compound **6**

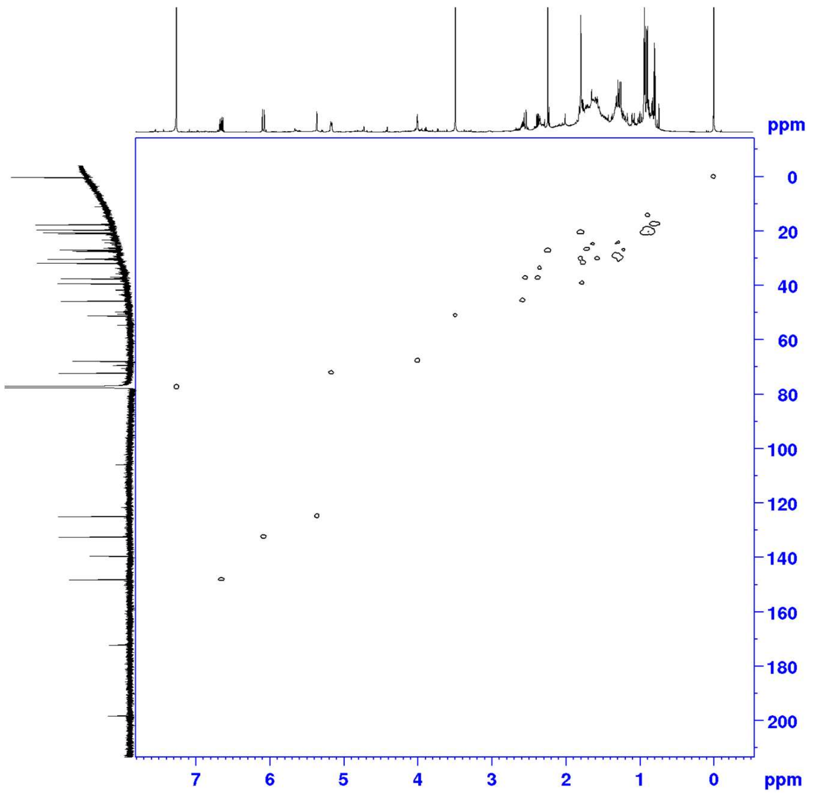

Figure S55. The HSQC spectrum of compound **6**

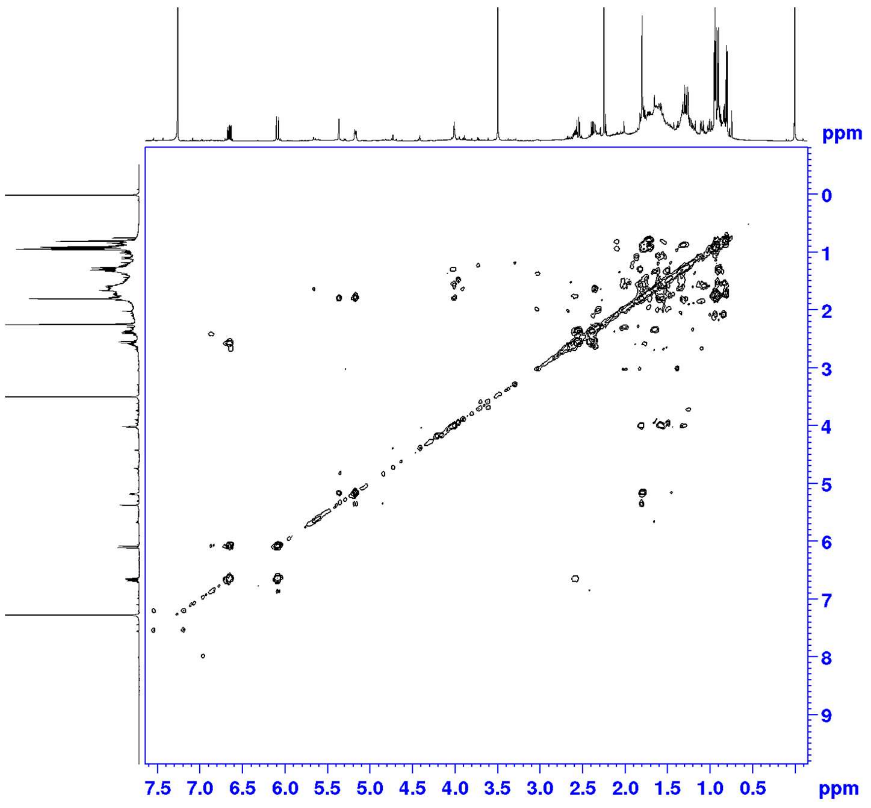

Figure S56. The ^{1}H-^{1}H COSY spectrum of compound **6**

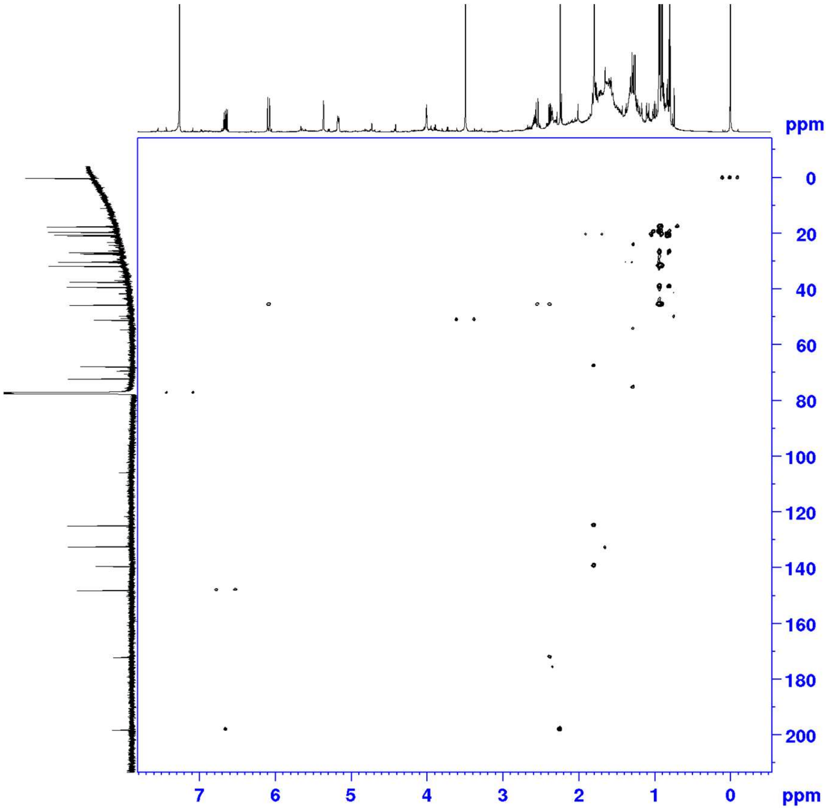

Figure S57. The HMBC spectrum of compound **6**

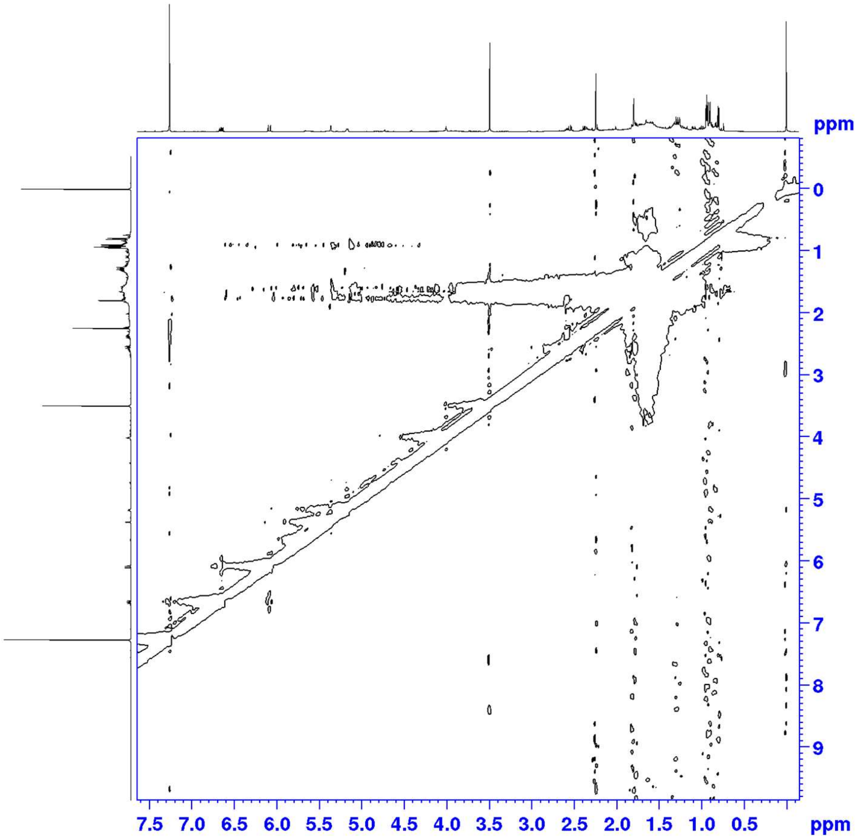

Figure S58. The NOESY spectrum of compound **6**

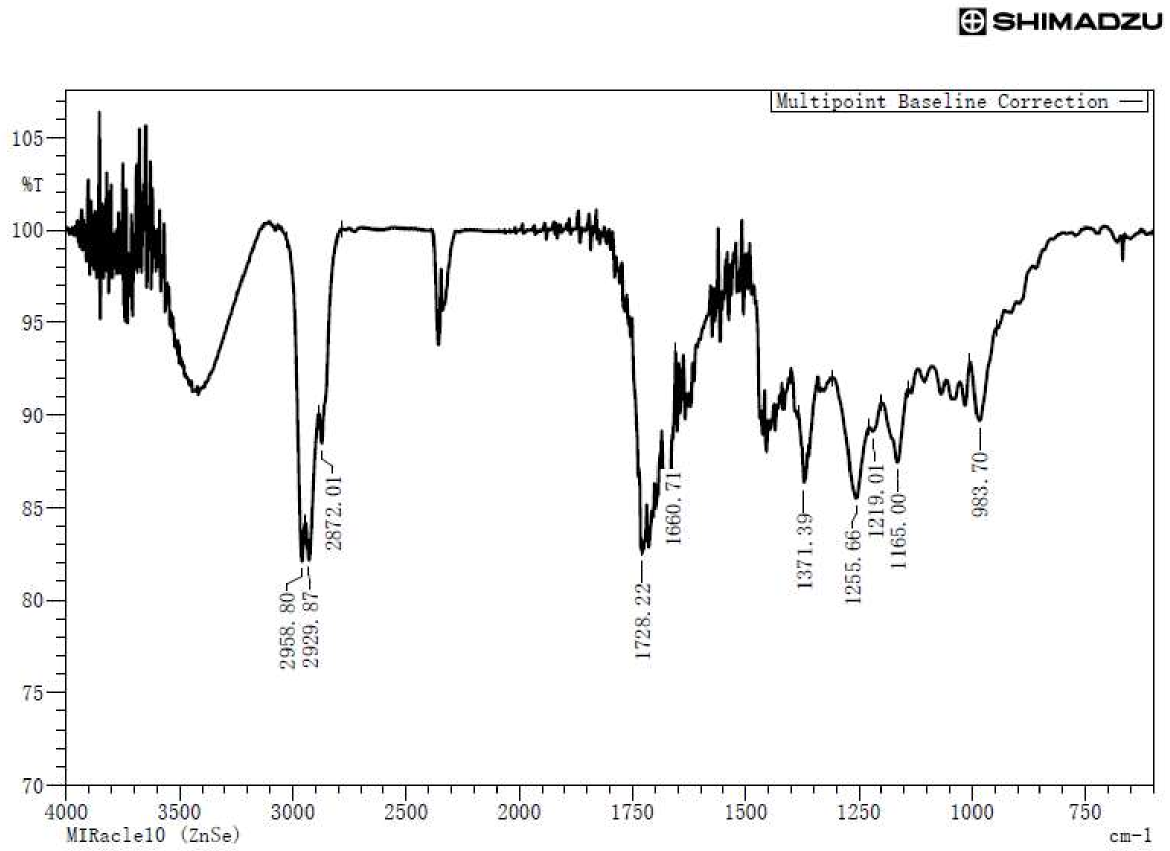

Figure S59. The IR spectrum of compound **6**

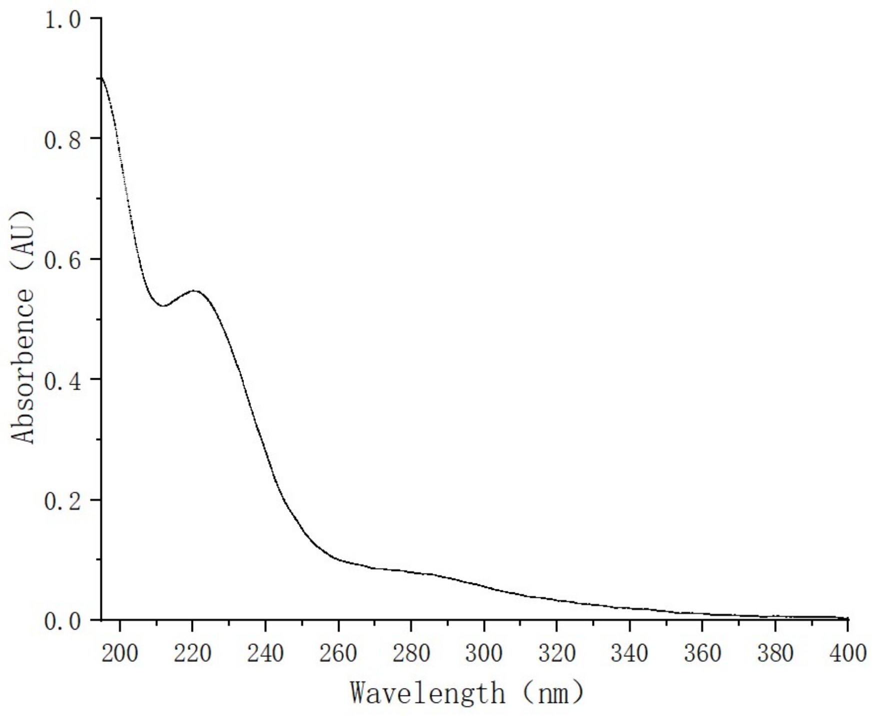

Figure S60. The UV spectrum of compound **6**

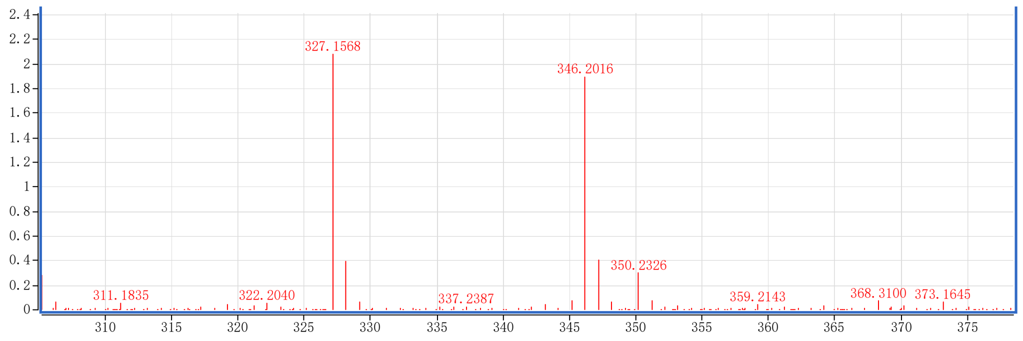

Formula	Score	Mass	Mass (MFG)	m/z (Calc)	Diff (ppm)	Ion Formula	m/z
$C_{18}H_{24}O_4$	99.95	304.1676	304.1675	327.1567	-0.39	$C_{18}H_{24}NaO_4$	327.1568

Figure S61. The HRESIMS spectrum of compound **7**

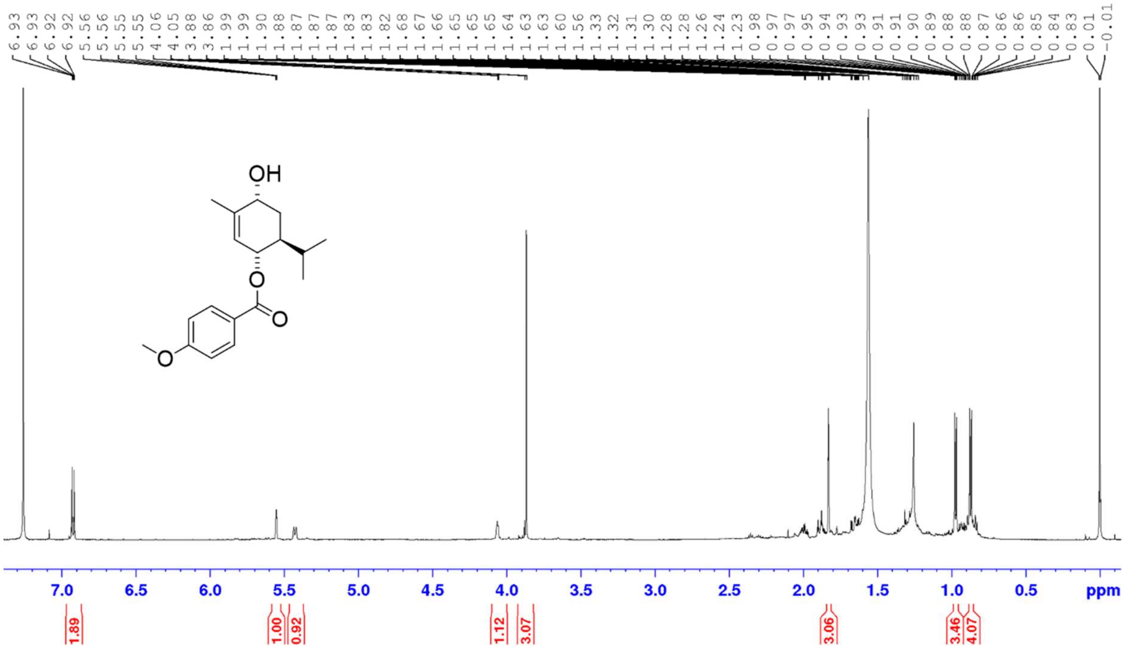

Figure S62. The ^{1}HNMR spectrum of compound **7**

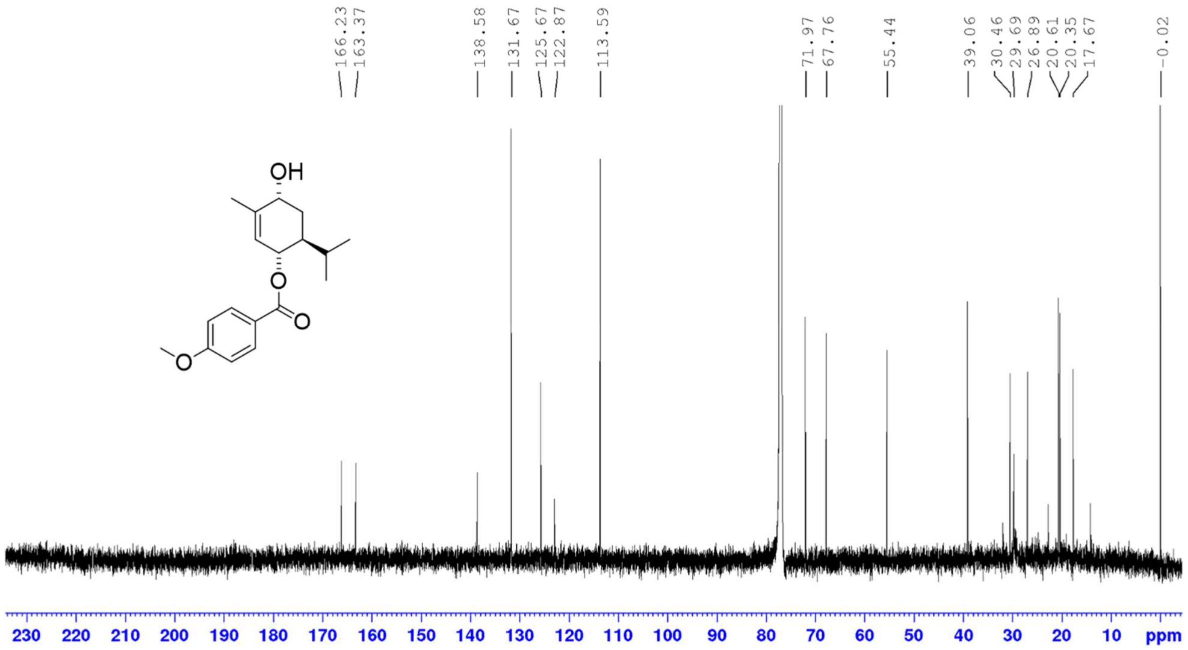

Figure S63. The ¹³CNMR spectrum of compound **7**

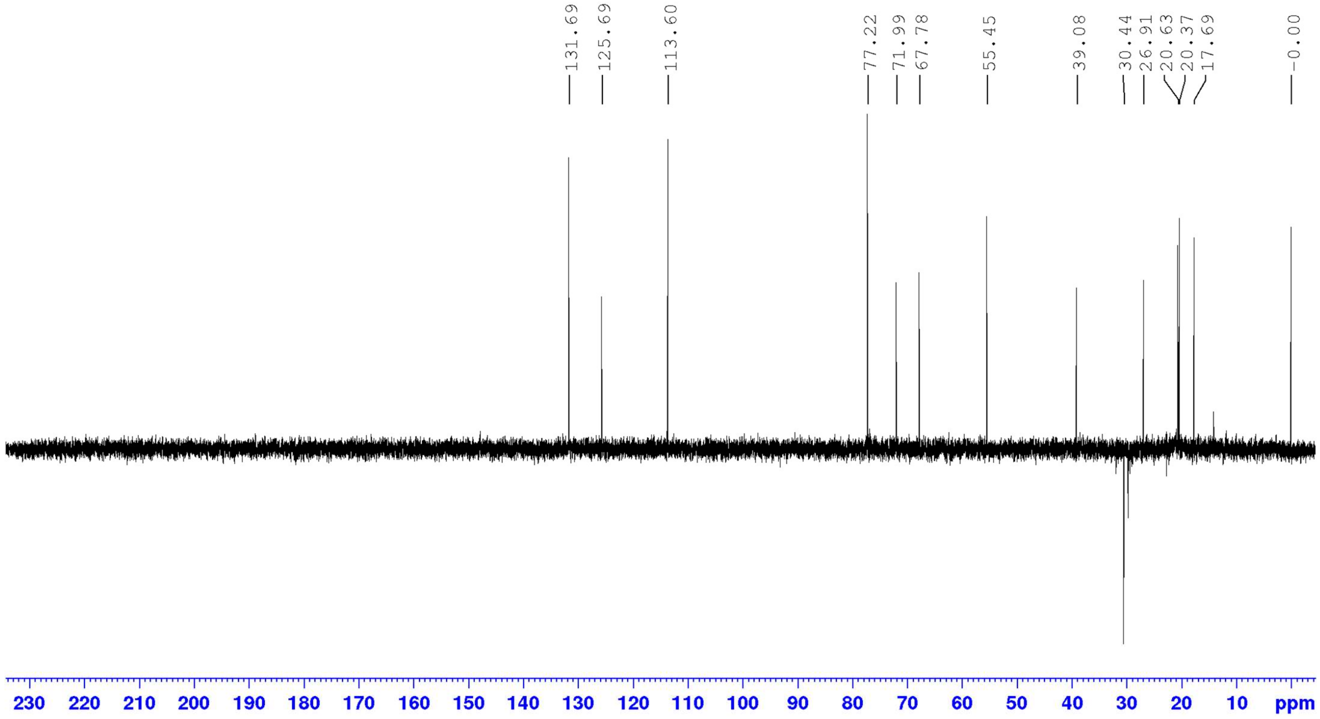

Figure S64. The DEPT135 spectrum of compound **7**

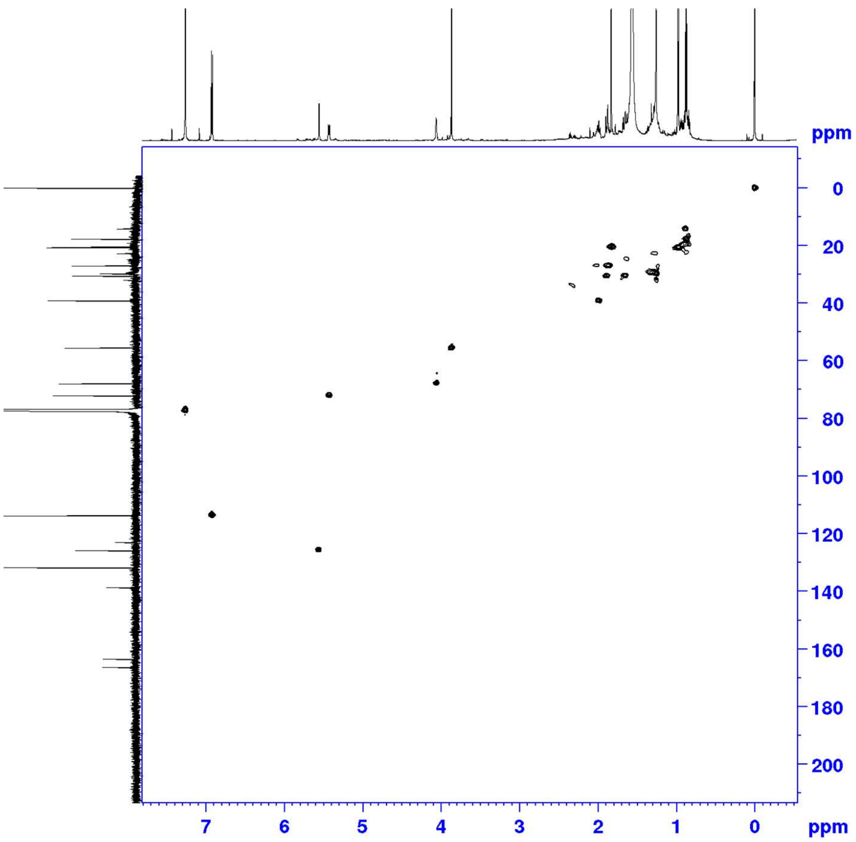

Figure S65. The HSQC spectrum of compound **7**

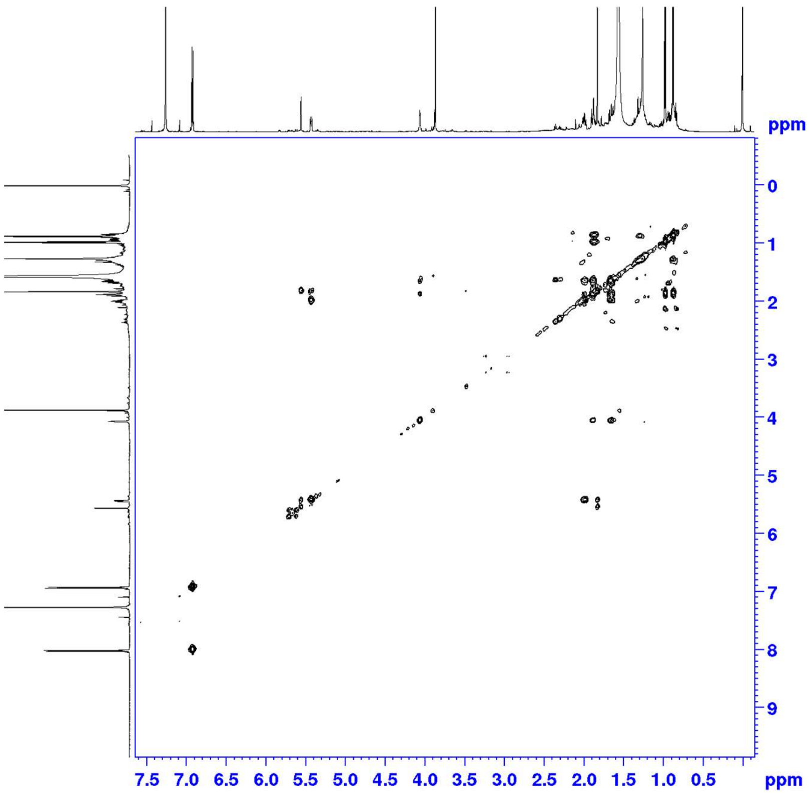

Figure S66. The ^{1}H-^{1}H COSY spectrum of compound **7**

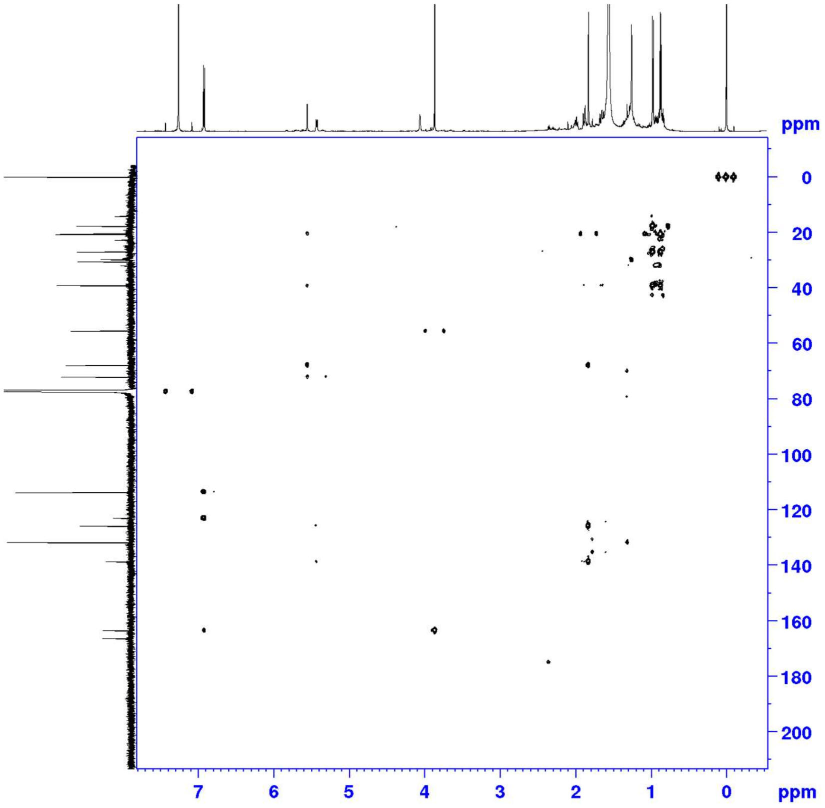

Figure S67. The HMBC spectrum of compound **7**

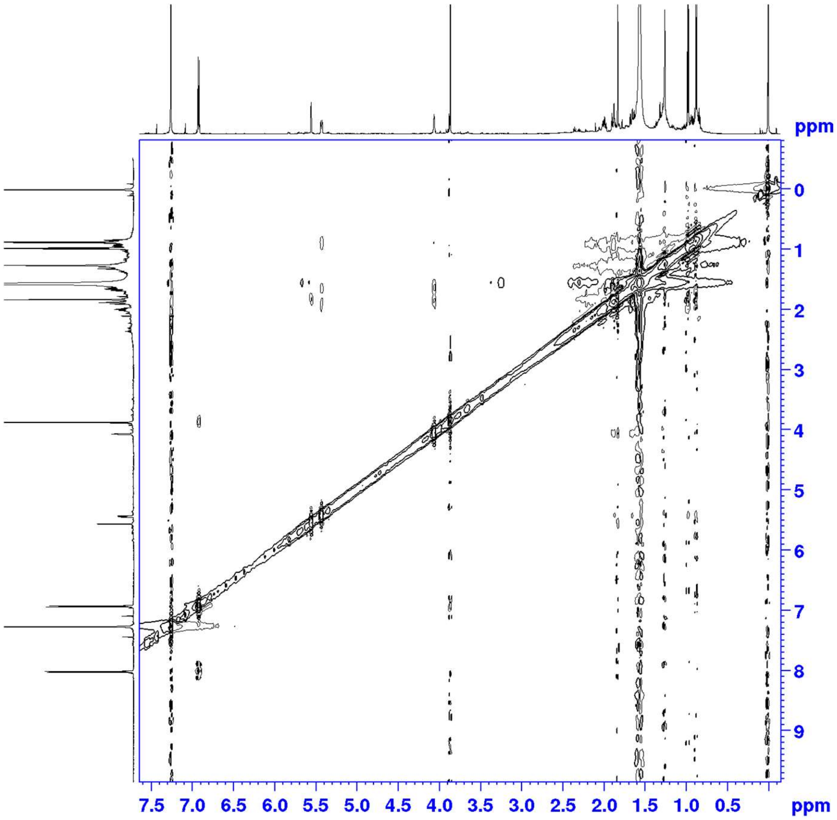

Figure S68. The NOESY spectrum of compound **7**

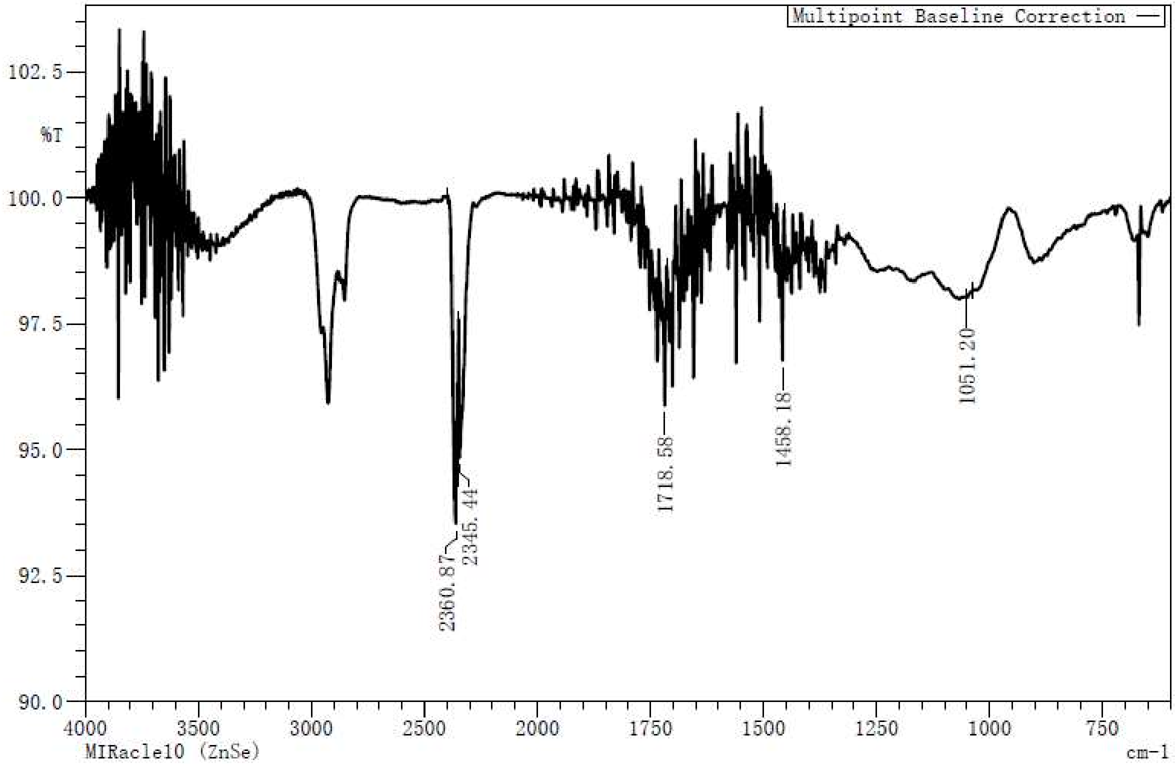

Figure S69. The IR spectrum of compound **7**

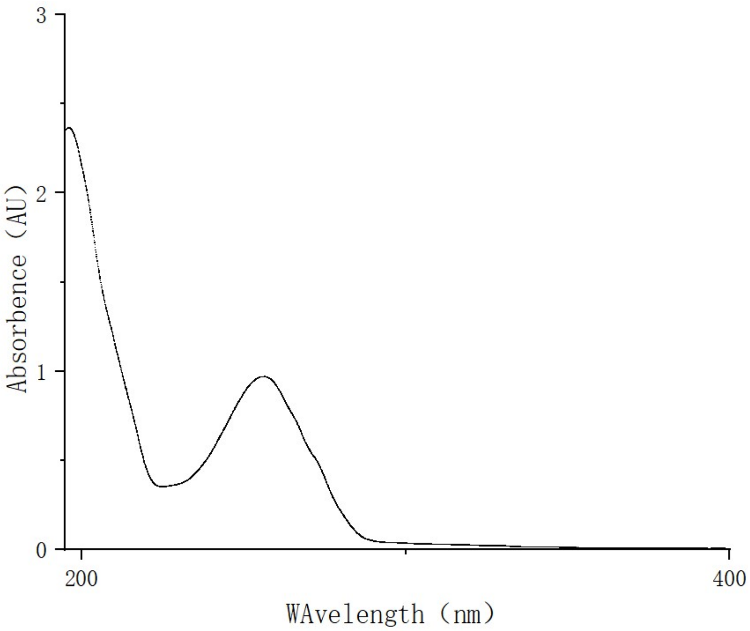

Figure S70. The UV spectrum of compound **7**

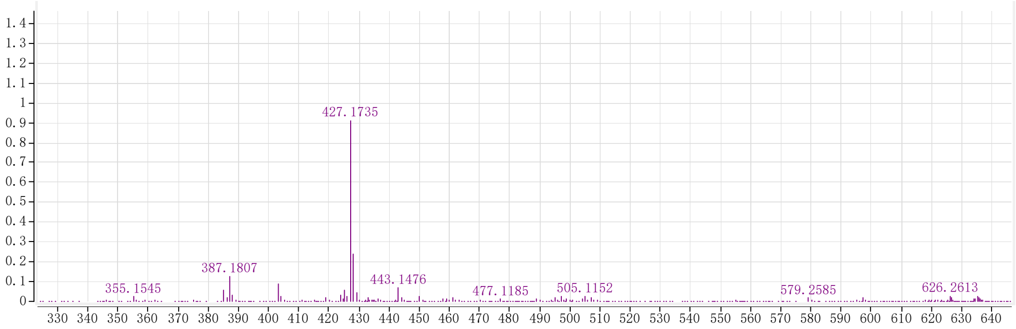

Formula	Score	Mass	Mass (MFG)	m/z (Calc)	Diff (ppm)	Ion Formula	m/z
$C_{2}2H_{28}O_{7}$	98.45	404.1843	404.1835	427.1727	-1.92	$C_{22}H_{28}NaO_{7}$	427.1735

Figure S71. The HRESIMS spectrum of compound **8**

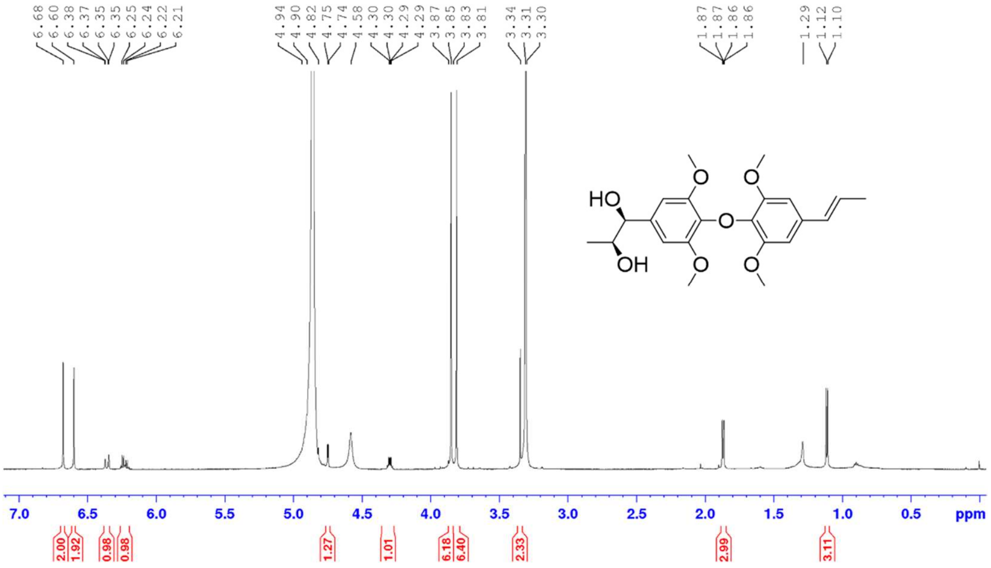

Figure S72. The ^{1}HNMR spectrum of compound **8**

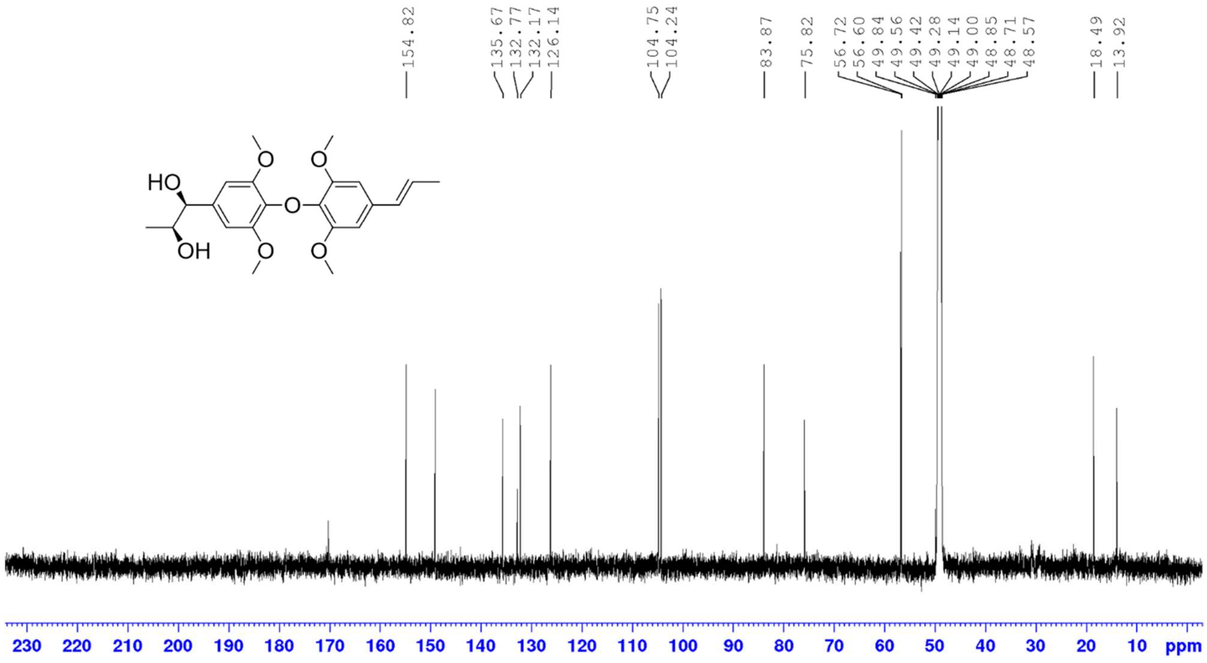

Figure S73. The ^{13}CNMR spectrum of compound **8**

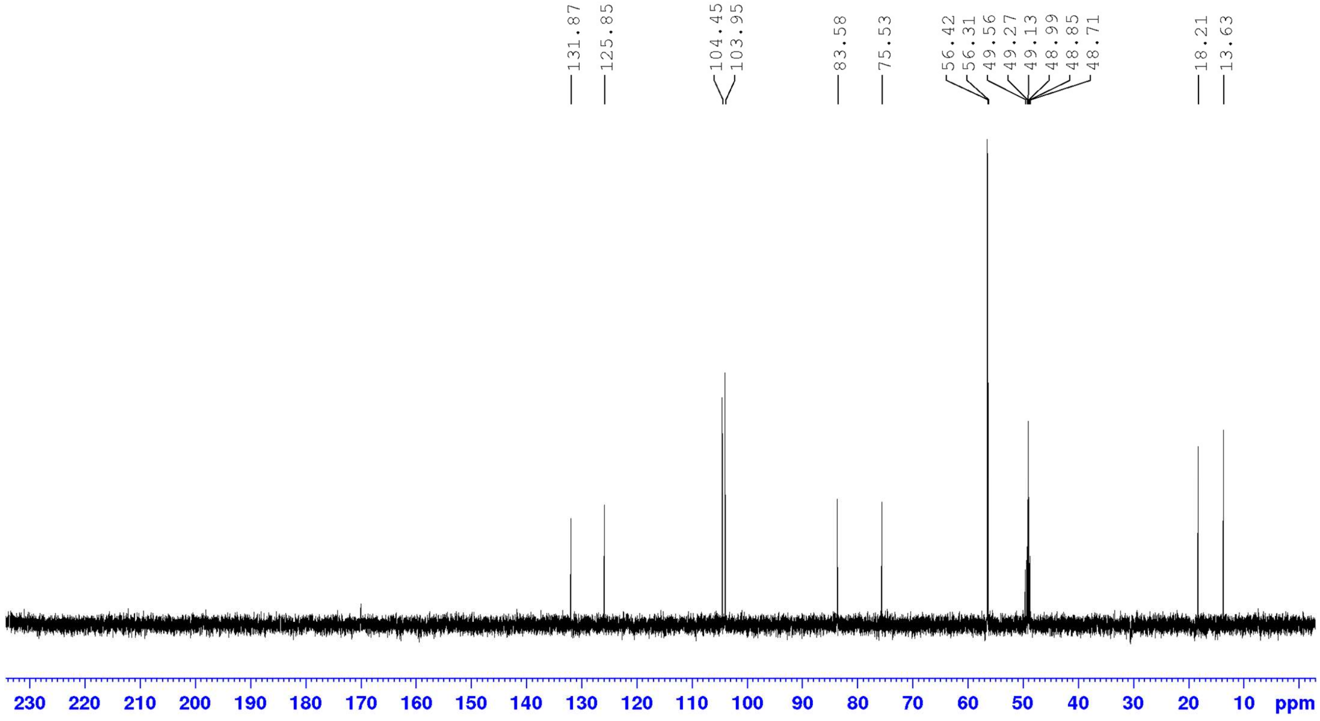

Figure S74. The DEPT135 spectrum of compound **8**

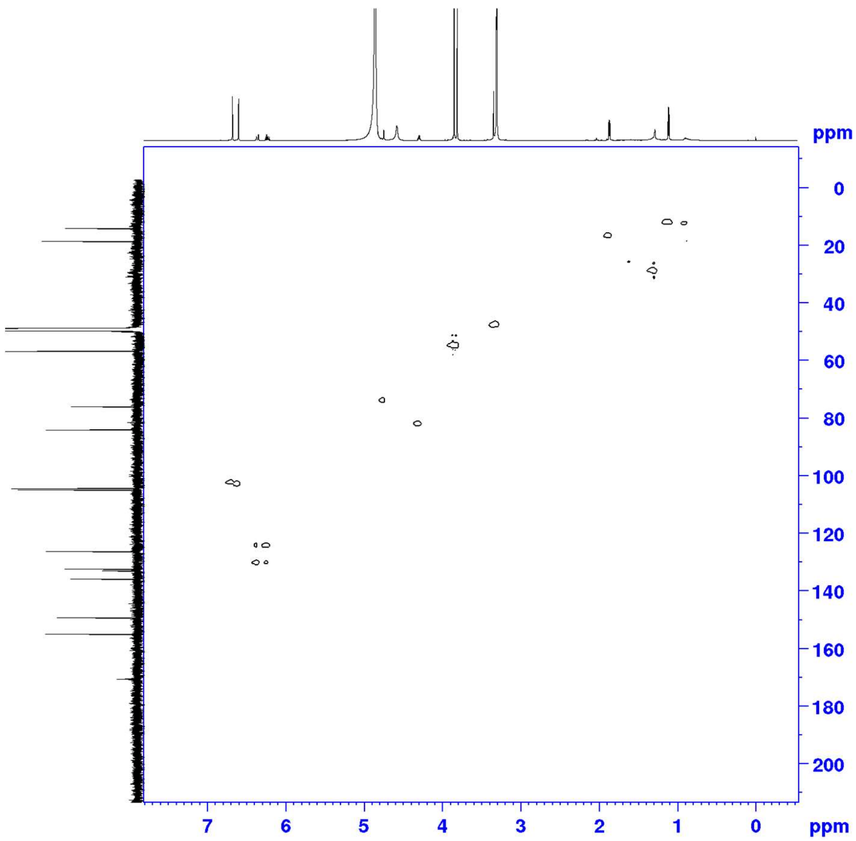

Figure S75. The HSQC spectrum of compound **8**

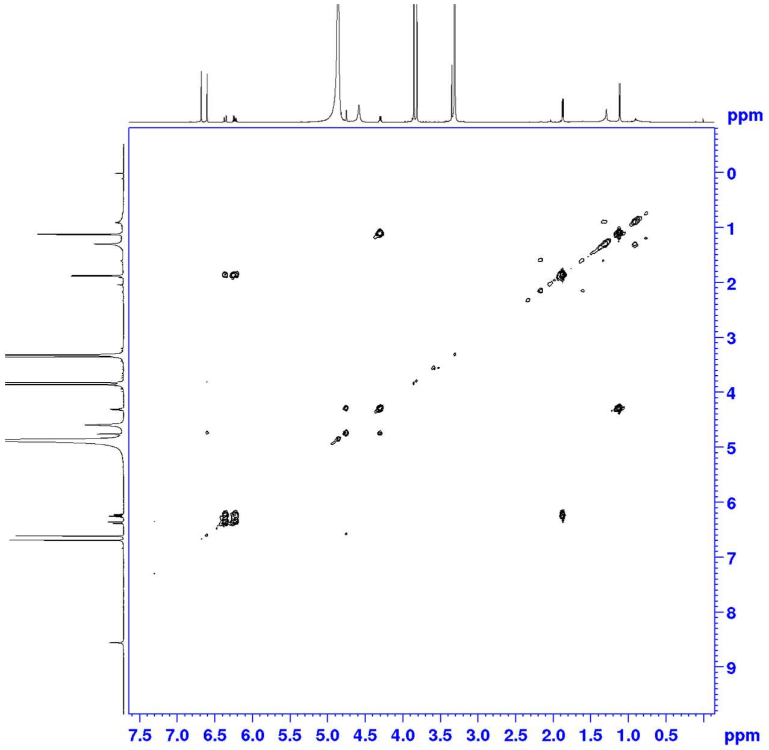

Figure S76. The ^1H-^1H COSY spectrum of compound **8**

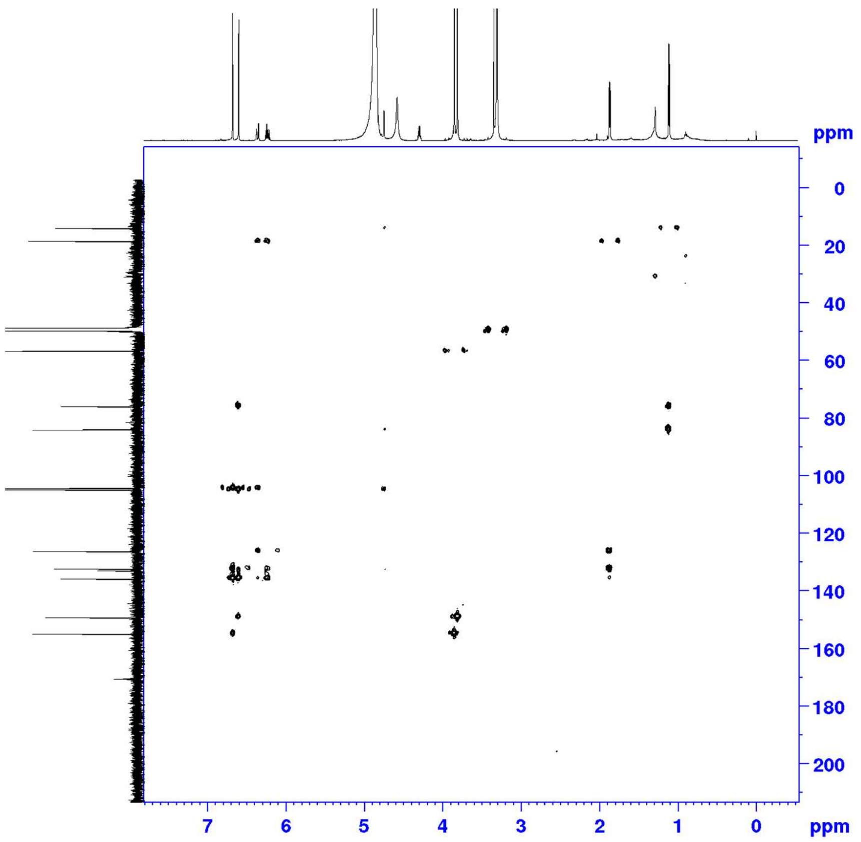

Figure S77. The HMBC spectrum of compound **8**

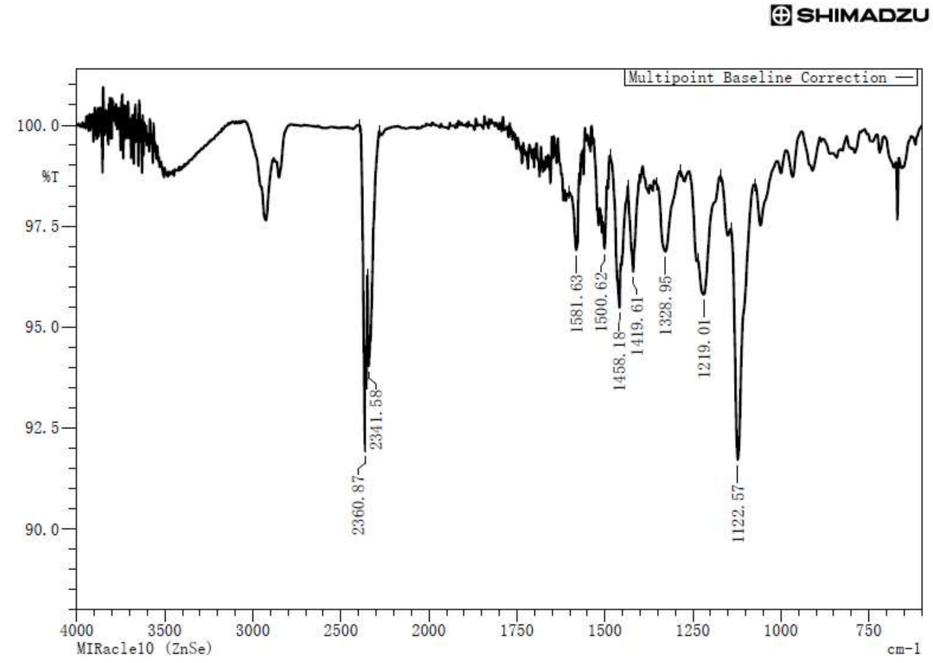

Figure S78. The IR spectrum of compound **8**

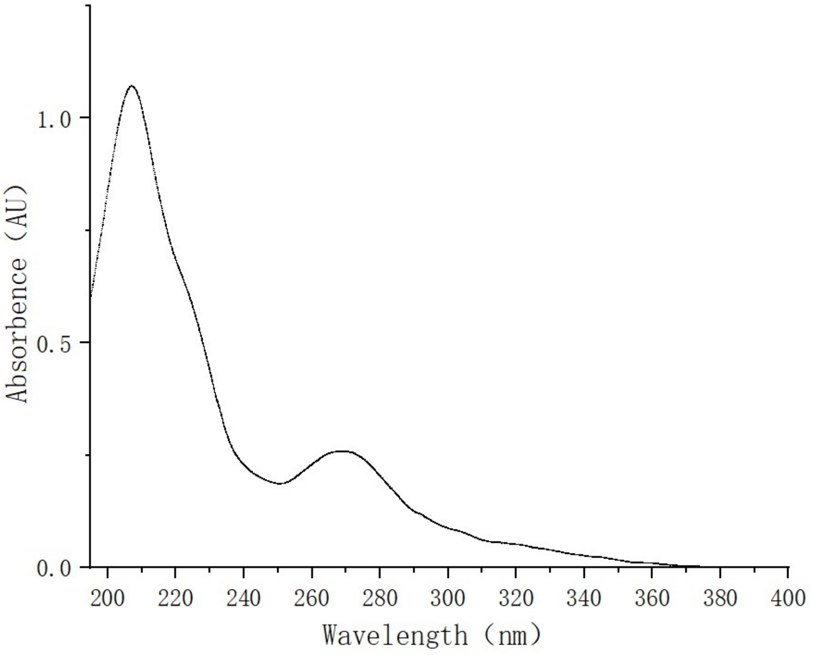

Figure S79. The IR spectrum of compound **8**

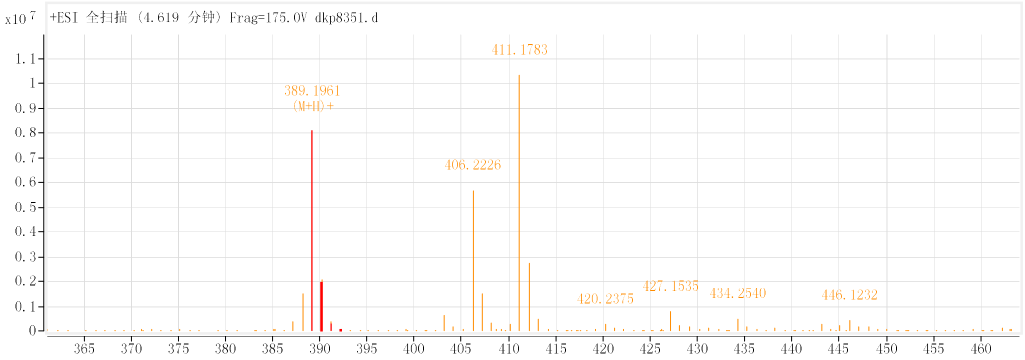

Formula	Score	Mass	Mass (MFG)	m/z (Calc)	Diff (ppm)	Ion Formula	m/z
$C_{22}H_{28}O_6$	99.85	388.1888	388.1886	389.1959	-0.61	$C_{22}H_{29}O_6$	389.1961

Figure S80. The HRESIMS spectrum of compound **9**

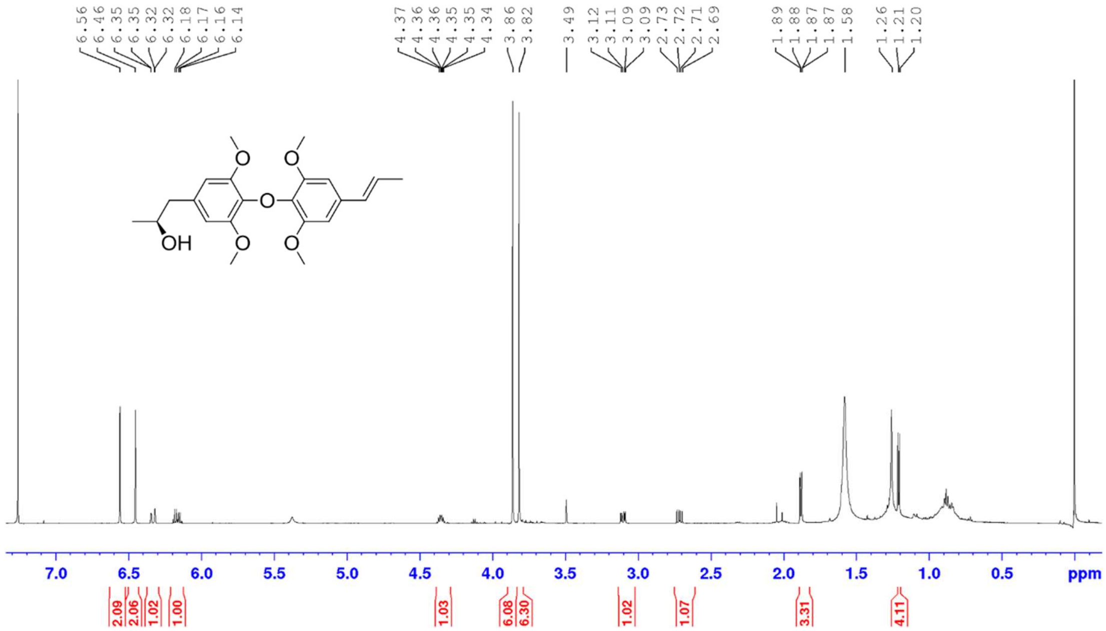

Figure S81. The ^{1}HNMR spectrum of compound **9**

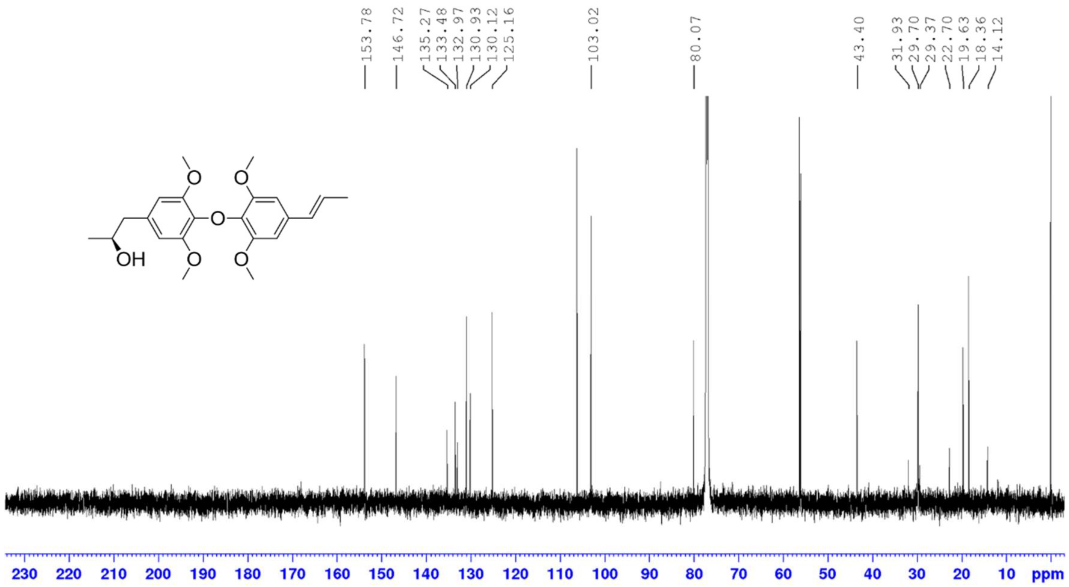

Figure S82. The ^{13}CNMR spectrum of compound **9**

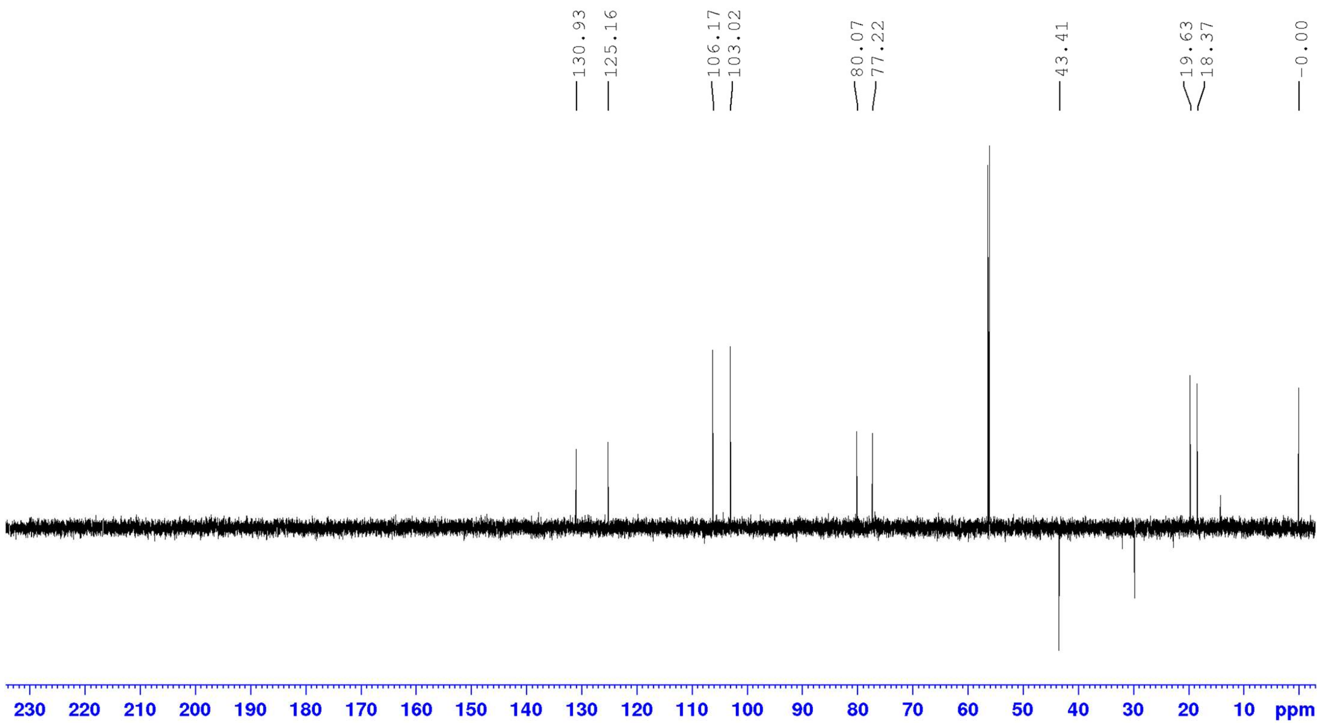

Figure S83. The DEPT135 spectrum of compound **9**

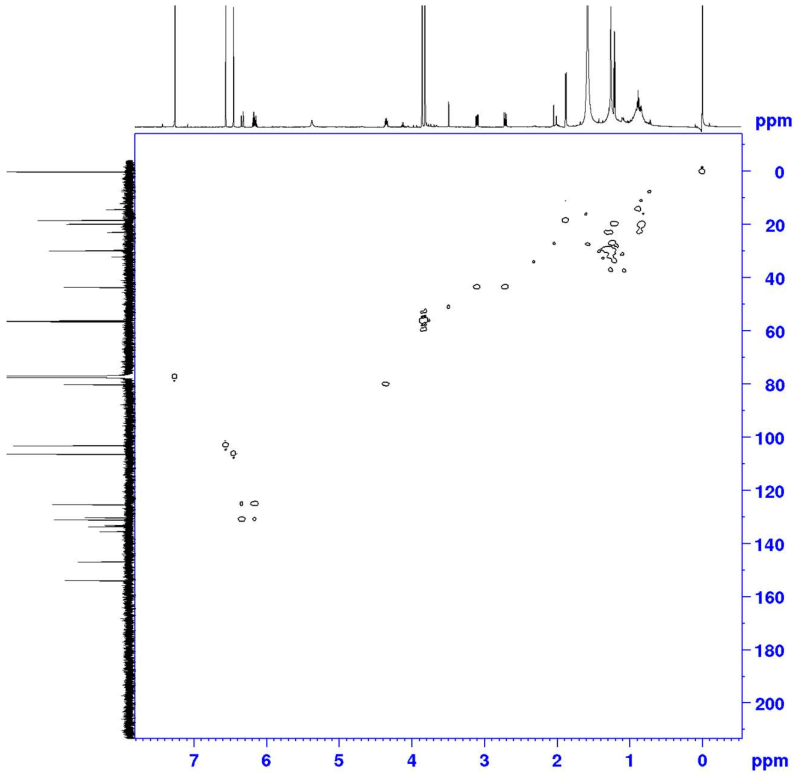

Figure S84. The HSQC spectrum of compound **9**

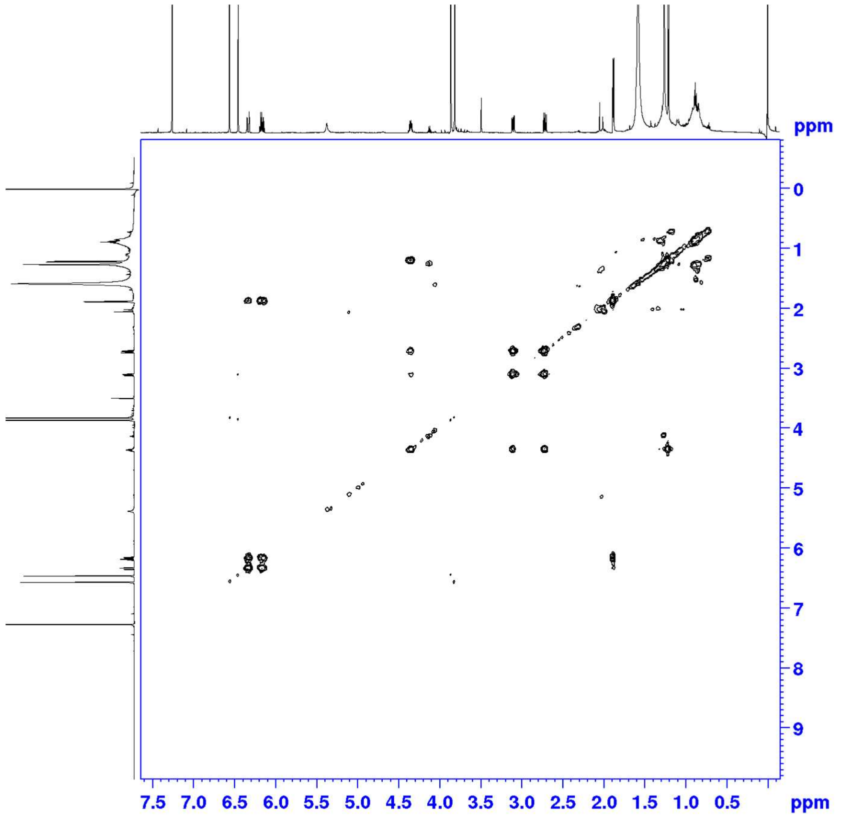

Figure S85. The ^{1}H-^{1}H COSY spectrum of compound **9**

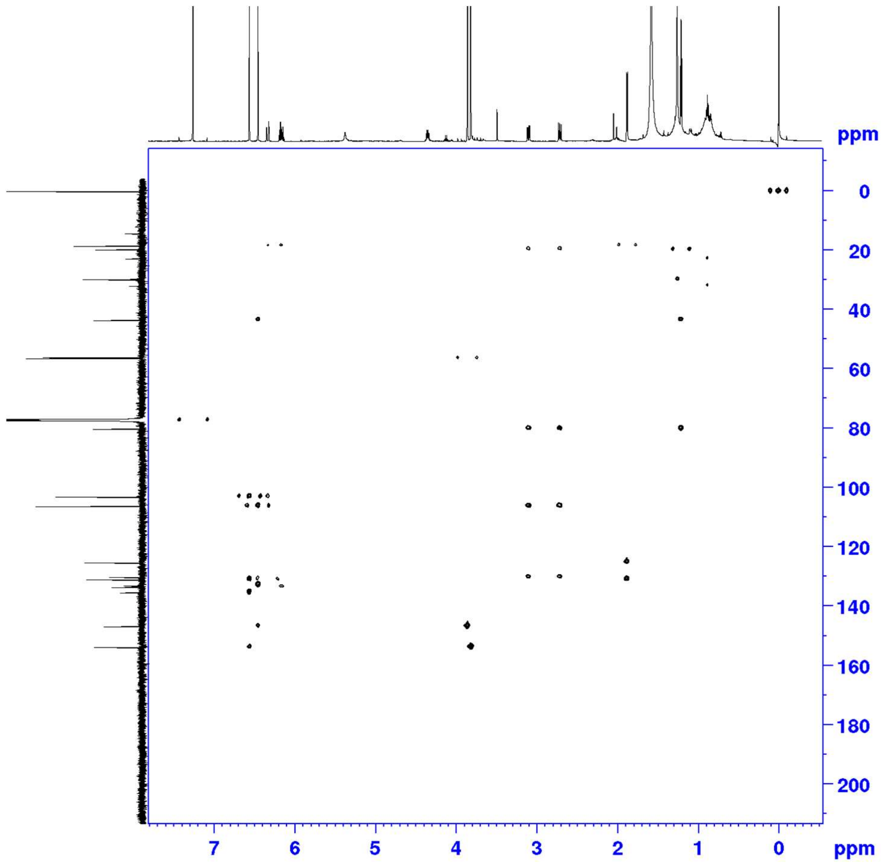

Figure S86. The HMBC spectrum of compound **9**

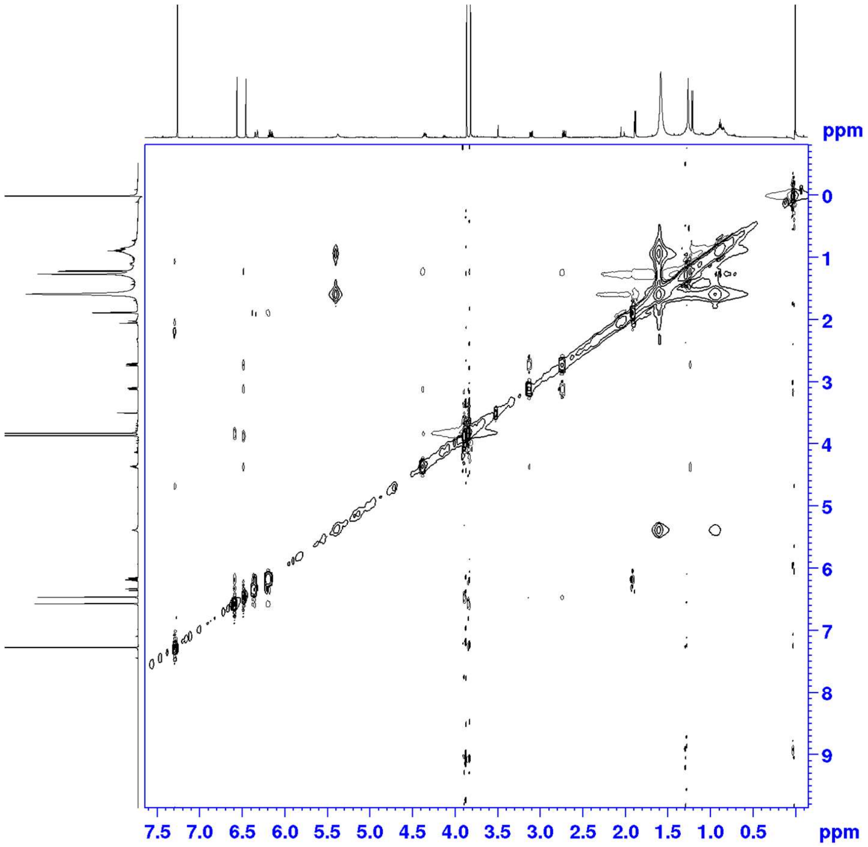

Figure S87. The NOESY spectrum of compound **9**

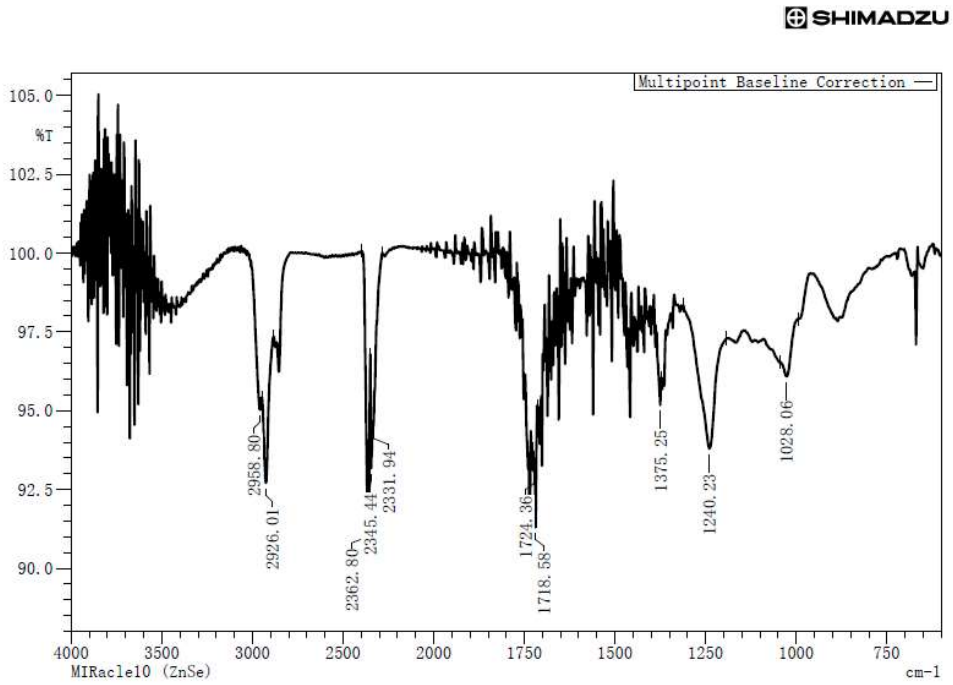

Figure S88. The IR spectrum of compound **9**

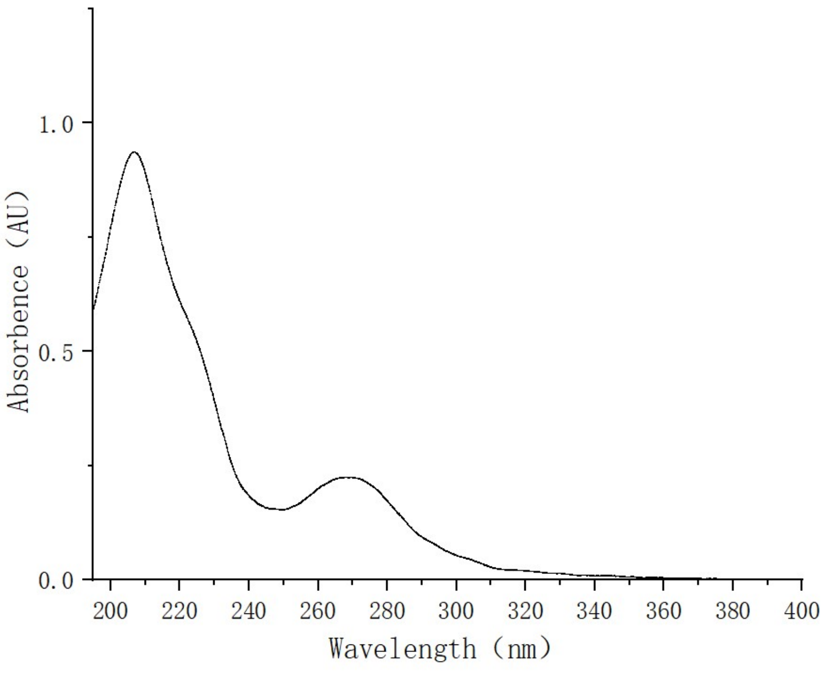

Figure S89. The UV spectrum of compound **9**

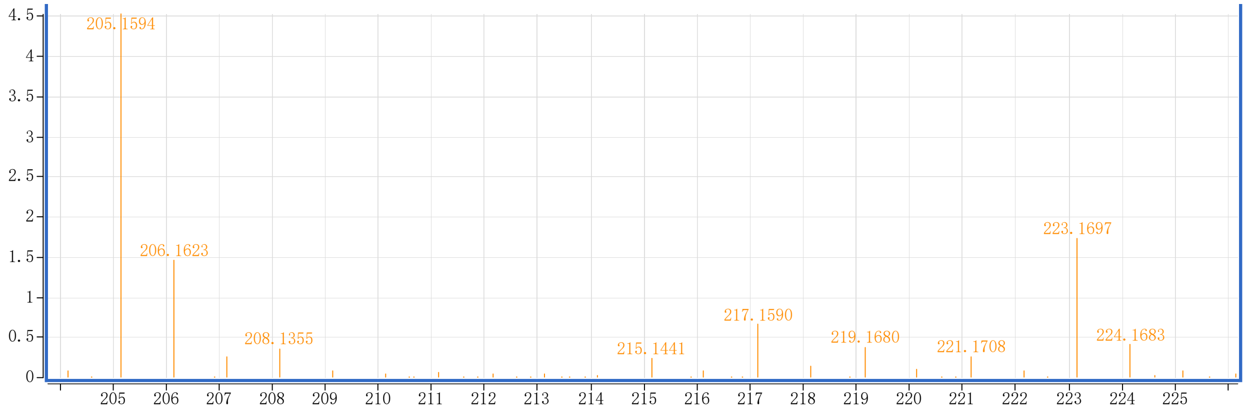

Formula	Score	Mass	Mass (MFG)	m/z (Calc)	Diff (ppm)	Ion Formula	m/z
$C_{14}H_{22}O_2$	99.12	222.1624	222.162	223.1693	-2	$C_{14}H_{23}O_2$	223.1697

Figure S90. The HRESIMS spectrum of compound **10**

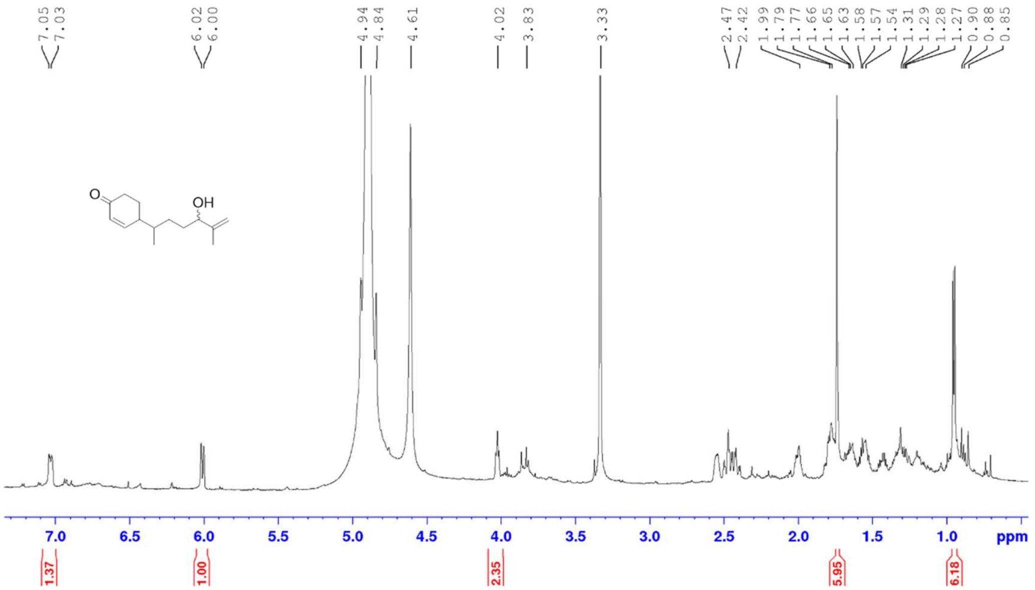

Figure S91. The ^{1}HNMR spectrum of compound **10**

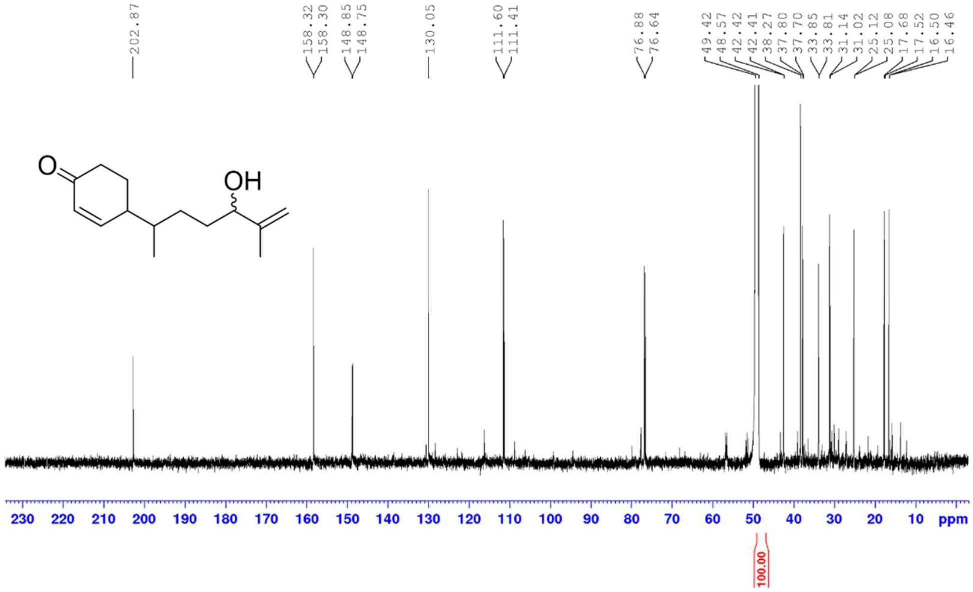

Figure S92. The ^{13}CNMR spectrum of compound **10**

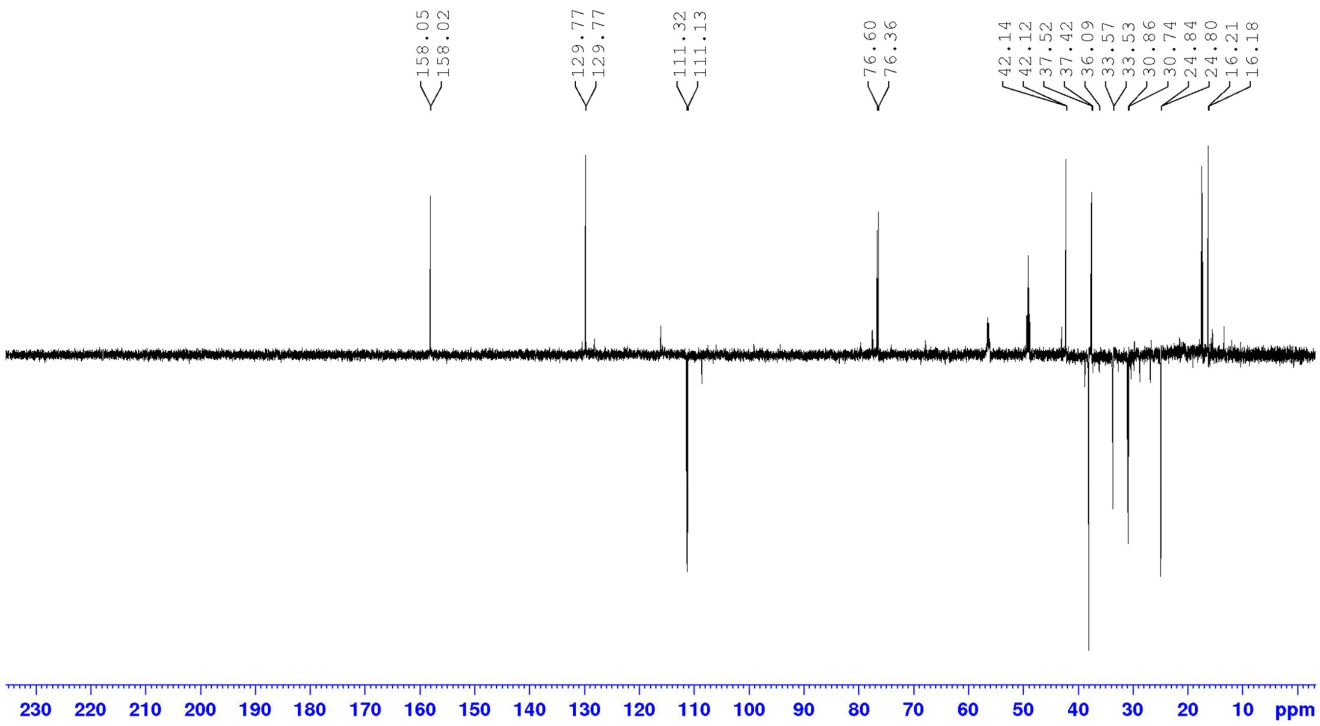

Figure S93. The DEPT135 spectrum of compound **10**

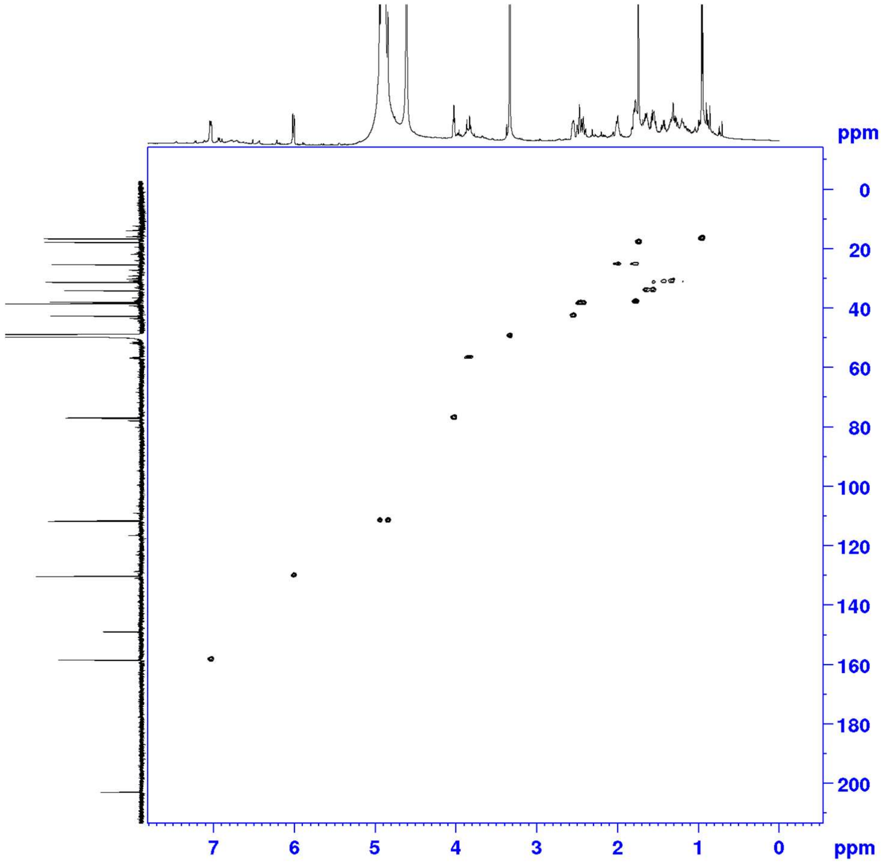

Figure S94. The HSQC spectrum of compound **10**

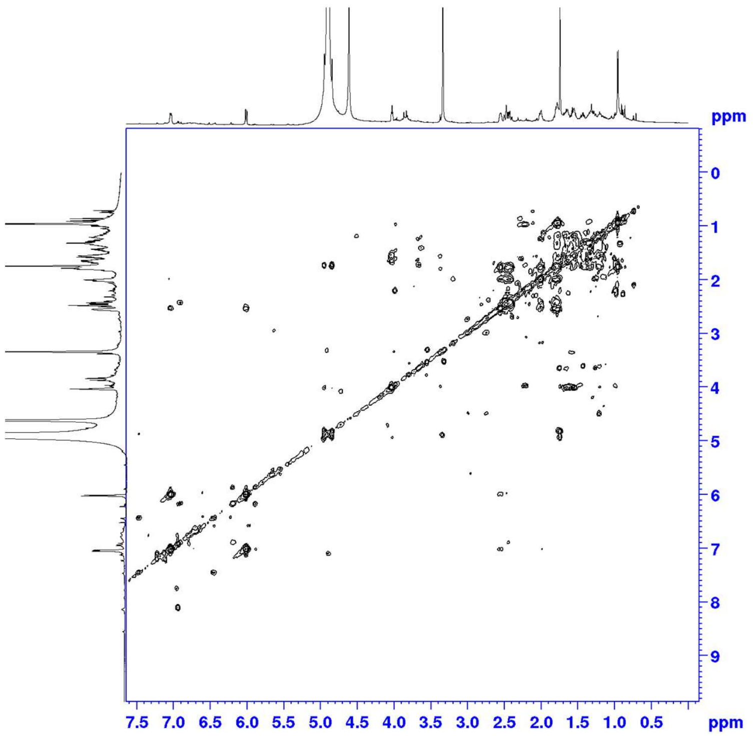

Figure S95. The ^{1}H-^{1}H COSY spectrum of compound **10**

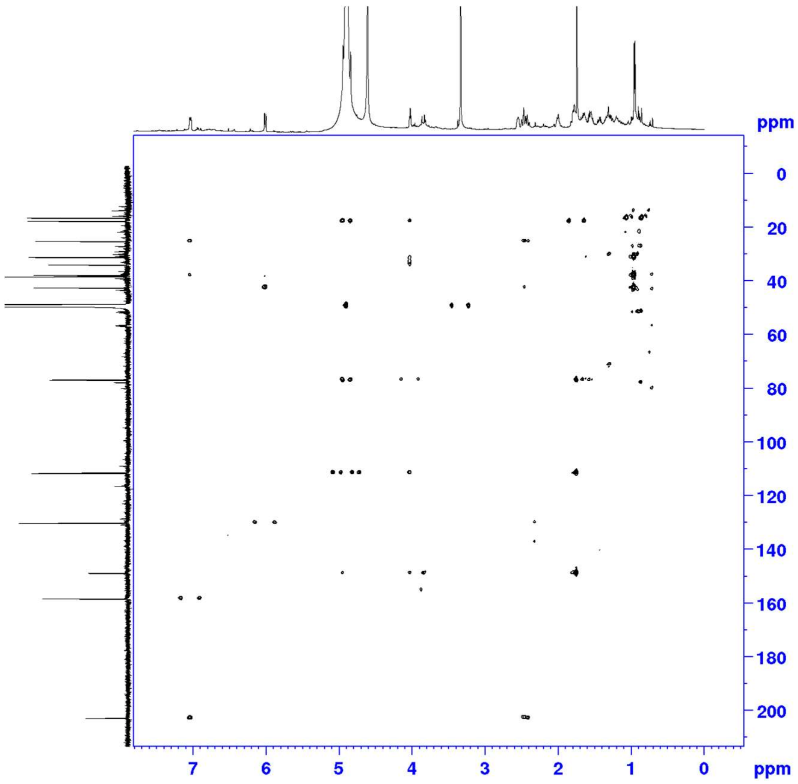

Figure S96. The HMBC spectrum of compound **10**

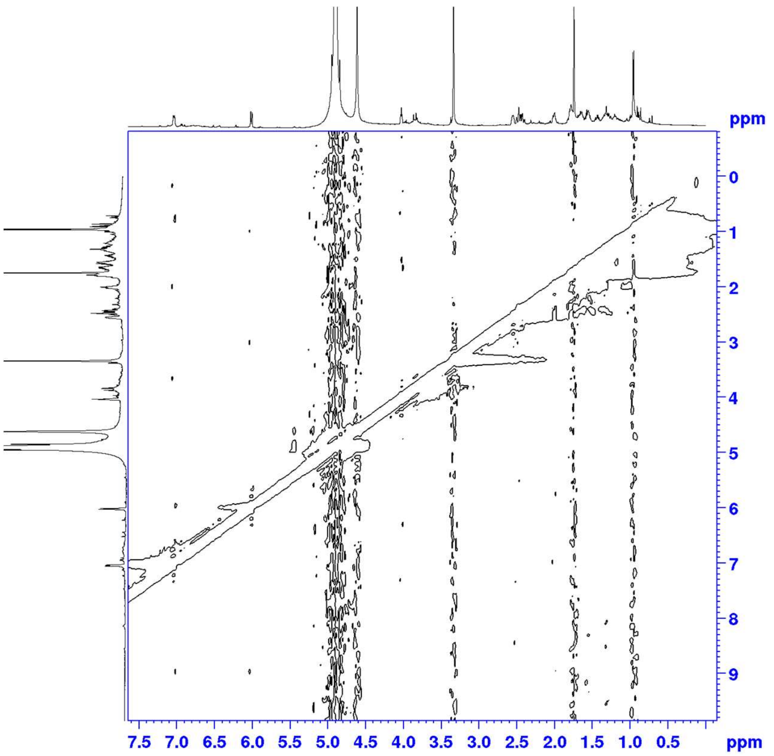

Figure S97. The NOESY spectrum of compound **10**

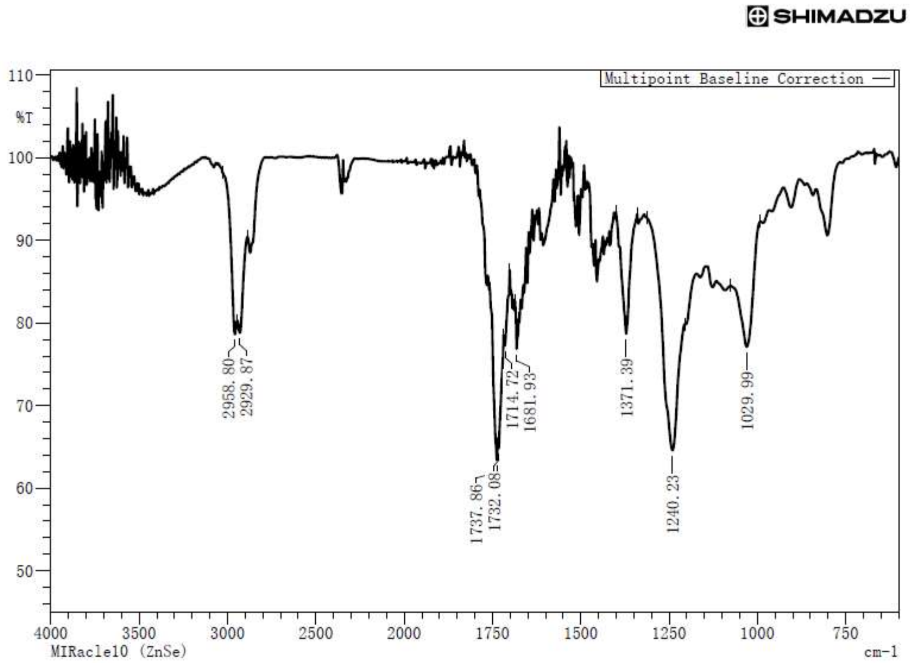

Figure S98. The IR spectrum of compound **10**

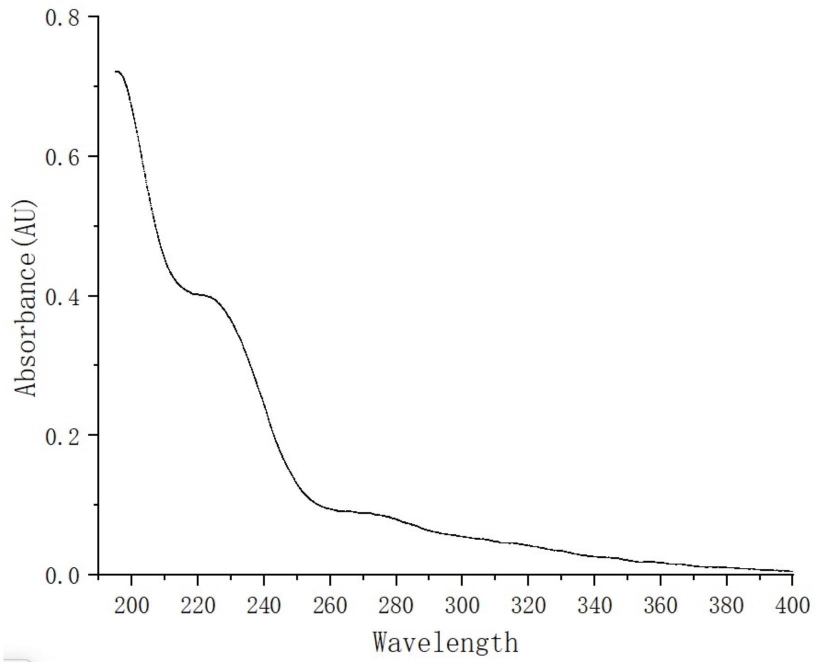

Figure S99. The UV spectrum of compound **10**

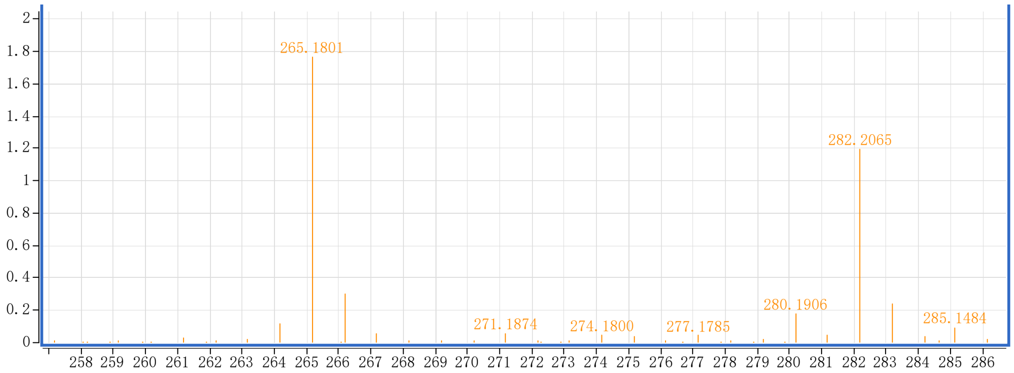

Formula	Score	Mass	Mass (MFG)	m/z (Calc)	Diff (ppm)	Ion Formula	m/z
$C_{14}H_{24}O_{3}$	99.69	364.1728	264.1725	265.1798	-1.06	$C_{14}H_{25}O_{3}$.	265.1801

Figure S100. The HRESIMS spectrum of compound **10a**

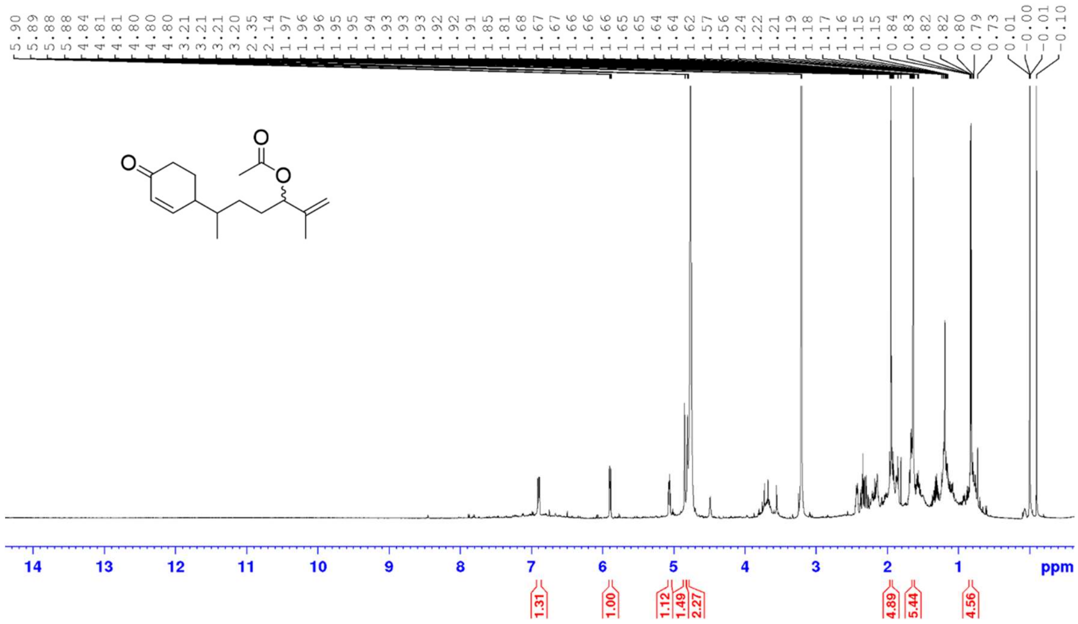

Figure S101. The ^{1}H NMR spectrum of compound **10a**

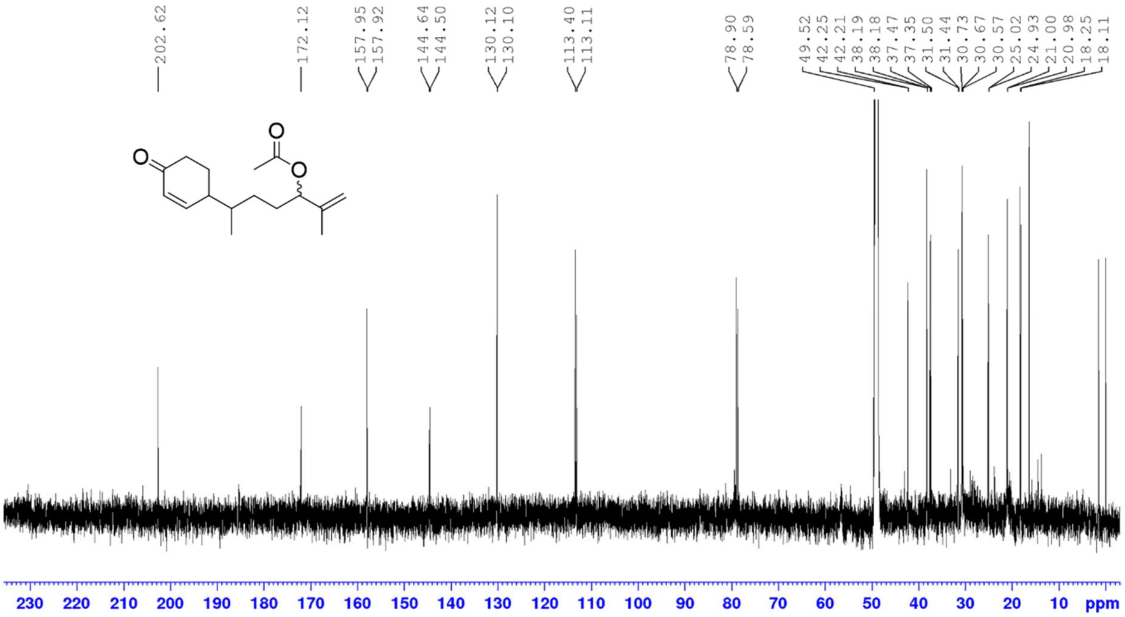

Figure S102. The ^{13}C NMR spectrum of compound **10a**

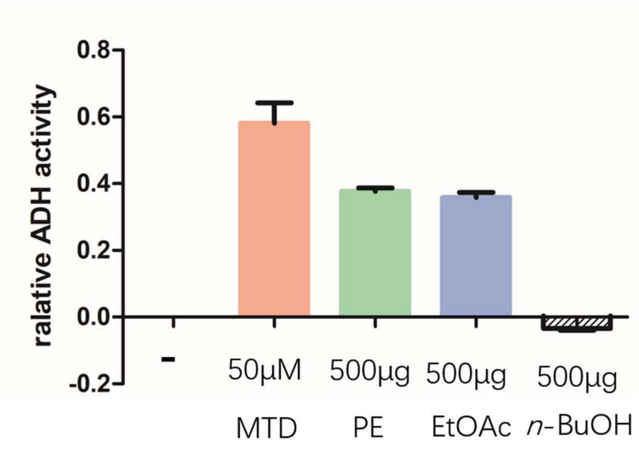

Figure S103. The effects of PE, EtOAc, and n-BuOH extracts on ADH activity.

Table S1. ^{1}H-NMR (600 MHz) and ^{13}C-NMR (150 MHz) data of compound **10a**.

**10a** (MeOD)	
δ_C	δ_H
202.8	
129.9	5.89, dd, 2.9, 10.5
158.1	6.89, brd, 10.5
42.2	2.43, m
25.0 (25.1)	1.84, 1.64, m
38.2	2.31, dd, 5.0, 12.6
	2.36, dt, 4.0, 12.6
37.7 (37.8)	1.67, m
30.5	1.32,1.19, m
31.5 (31.6)	1.65, 1.57, m
79.3 (79.3)	5.06, m
148.7 (148.8)	
18.0 (18.1)	1.63, s
113.4 (113.6)	4.83, brs
	4.94, brs
16.2	0.83, d, 6.9
172.2	
21.0	1.94, s
